# Supplementary material for: Physicochemical properties of stingless bees (Meliponula beccarii) honey in Dandi and Meta Robi districts of West Shewa zone, Ethiopia
Source: PLoS One. 2024 Dec 12;19(12):e0311725. doi: 10.1371/journal.pone.0311725 (PMC11637301; doi:10.1371/journal.pone.0311725)
Supplement: Attachment — Submitted filename: to review PONE-D-24-28380 20 July.pdf [file pone.0311725.s002.pdf]

PLOS ONE

Properties of stingless bees (Meliponula beccarii) honey in Dandi and Meta Robi districts of West Shewa zone, Ethiopia

--Manuscript Draft--

**Manuscript Number:**	PONE-D-24-28380
**Article Type:**	Research Article
**Full Title:**	Properties of stingless bees (Meliponula beccarii) honey in Dandi and Meta Robi districts of West Shewa zone, Ethiopia
**Short Title:**	Physicochemical Profile of Ground-Nesting Stingless Bee Honey in Ethiopia
**Corresponding Author:**	Desalegn Begna, Ph.D<br>Policy Studies Institute, Ethiopia<br>Addis Ababa, ETHIOPIA
**Keywords:**	Stingless bee, Physicochemical, honey, properties, moisture
**Abstract:**	The study was conducted to characterize the Physicochemical properties of honey produced from underground nesting stingless bees (/Meliponula beccarii) in the Dandi and Meta Robi districts of the west Shewa zone, Ethiopia. A total of 24 honey samples with each 0.58–0.69-liter were collected through an excavation of natural nests and analyzed in the laboratory for various Physicochemical properties. The evaluated Physicochemical properties indicated an overall mean of 306.64 ±87.95 acidity mill./kg, 28.05±3.52 moisture content, 1.31±0.44 ms/cm electric conductivity, 3.29±0.16 pH, 0.89±1.14 9 mg/kg HMF, 0.63±0.24% mineral (ash), 9.39±4.26 glucose, 0.24±0.01g/100 sucrose, 10.81±0.4.95g/100g maltose and 16.57±2.55g/100g fructose. The differences between the two districts honey samples are significant (p> 0.05) in glucose value and considerable with acidity, moisture contents and pH values. The distinctive low HMF, high acidity and low pH values of the stingless bee honey of this study may represent freshness and good quality of the honey and indicative medicinal potential.<br>This study evaluated physicho-chemical properties of honey produced by ground nesting stingless bee (Meliponula beccarii) in two districts of West Shewa zone, Ethiopia and demonstrated the honeys have distinctive physicochemical properties from A.mellifera that may backup its uses as traditional medicine. Further detailed study on ground nesting and other stingless species honey medicinal values is recommended to provide scientific evidences.
**Order of Authors:**	Desalegn Begna, Ph.D
	Girma Motuma, Msc
	Shambel Boki, Msc
	Niguse Bekele, PhD
	Tammiru Kuru, Msc
	Achalu Chimdi, PhD
**Additional Information:**	
**Question**	**Response**
**Financial Disclosure**<br><br>Enter a financial disclosure statement that describes the sources of funding for the work included in this submission. Review the submission guidelines for detailed requirements. View published research articles from *PLOS ONE* for specific examples.	The author(s) received no specific funding for this work.

This statement is required for submission and **will appear in the published article** if the submission is accepted. Please make sure it is accurate.<br><br>**Funded studies**<br>Enter a statement with the following details:<br>• Initials of the authors who received each award<br>• Grant numbers awarded to each author<br>• The full name of each funder<br>• URL of each funder website<br>• Did the sponsors or funders play any role in the study design, data collection and analysis, decision to publish, or preparation of the manuscript?<br><br>Did you receive funding for this work?	
**Competing Interests**<br><br>Use the instructions below to enter a competing interest statement for this submission. On behalf of all authors, disclose any competing interests that could be perceived to bias this work—acknowledging all financial support and any other relevant financial or non-financial competing interests.<br><br>This statement is required for submission and **will appear in the published article** if the submission is accepted. Please make sure it is accurate and that any funding sources listed in your Funding Information later in the submission form are also declared in your Financial Disclosure statement.<br><br>View published research articles from *PLOS ONE* for specific examples.	The authors have declared that no competing interests exist

**NO authors have competing interests**<br><br>Enter: *The authors have declared that no competing interests exist.*<br><br>**Authors with competing interests**<br><br>Enter competing interest details beginning with this statement:<br><br>*I have read the journal's policy and the authors of this manuscript have the following competing interests: [insert competing interests here]*<br><br>* typeset	
**Ethics Statement**<br><br>Enter an ethics statement for this submission. This statement is required if the study involved:<br><br>• Human participants<br>• Human specimens or tissue<br>• Vertebrate animals or cephalopods<br>• Vertebrate embryos or tissues<br>• Field research<br><br>Write "N/A" if the submission does not require an ethics statement.<br><br>General guidance is provided below. Consult the submission guidelines for detailed instructions. **Make sure that all information entered here is included in the Methods section of the manuscript.**	N/A

Powered by Editorial Manager® and ProduXion Manager® from Aries Systems Corporation

**Format for specific study types**<br><br>**Human Subject Research (involving human participants and/or tissue)**<br>• Give the name of the institutional review board or ethics committee that approved the study<br>• Include the approval number and/or a statement indicating approval of this research<br>• Indicate the form of consent obtained (written/oral) or the reason that consent was not obtained (e.g. the data were analyzed anonymously)<br><br>**Animal Research (involving vertebrate animals, embryos or tissues)**<br>• Provide the name of the Institutional Animal Care and Use Committee (IACUC) or other relevant ethics board that reviewed the study protocol, and indicate whether they approved this research or granted a formal waiver of ethical approval<br>• Include an approval number if one was obtained<br>• If the study involved *non-human primates*, add *additional details* about animal welfare and steps taken to ameliorate suffering<br>• If anesthesia, euthanasia, or any kind of animal sacrifice is part of the study, include briefly which substances and/or methods were applied<br><br>**Field Research**<br><br>Include the following details if this study involves the collection of plant, animal, or other materials from a natural setting:<br>• Field permit number<br>• Name of the institution or relevant body that granted permission	
**Data Availability**<br><br>Authors are required to make all data underlying the findings described fully available, without restriction, and from the time of publication. PLOS allows rare exceptions to address legal and ethical concerns. See the PLOS Data Policy and FAQ for detailed information.	Yes - all data are fully available without restriction

A Data Availability Statement describing where the data can be found is required at submission. Your answers to this question constitute the Data Availability Statement and **will be published in the article**, if accepted.<br><br>**Important:** Stating 'data available on request from the author' is not sufficient. If your data are only available upon request, select 'No' for the first question and explain your exceptional situation in the text box.<br><br>Do the authors confirm that all data underlying the findings described in their manuscript are fully available without restriction?	
**Describe where the data may be found in full sentences. If you are copying our sample text, replace any instances of XXX with the appropriate details.**<br><br>• If the data are **held or will be held in a public repository**, include URLs, accession numbers or DOIs. If this information will only be available after acceptance, indicate this by ticking the box below. For example: *All XXX files are available from the XXX database (accession number(s) XXX, XXX.)*.<br>• If the data are all contained **within the manuscript and/or Supporting Information files**, enter the following: *All relevant data are within the manuscript and its Supporting Information files.*<br>• If neither of these applies but you are able to provide **details of access elsewhere**, with or without limitations, please do so. For example:<br><br>*Data cannot be shared publicly because of [XXX]. Data are available from the XXX Institutional Data Access / Ethics Committee (contact via XXX) for researchers who meet the criteria for access to confidential data.*<br><br>*The data underlying the results presented in the study are available from (include the name of the third party*	All relevant data are within the manuscript

and contact information or URL).<br>• This text is appropriate if the data are owned by a third party and authors do not have permission to share the data.<br><br>* typeset	
Additional data availability information:	

Manuscript

Click here to access/download;Manuscript;MS of stingless Honey property_PLoS ONE.docx

Title: **Properties of stingless bees (*Meliponula beccarii*) honey in Dandi and Meta Robi districts of West Shewa zone, Ethiopia**

Desalegn Begna[1], Girma Motuma[2], Shambel Boki[2], Niguse Bekele[2], Tamiru kuru[2], Achalu Chimdi[2]

[1]Policy study institute and [2]Ambo university Mamo Mezemir Campus

Correspondence author: Desalegn Begna; apis_1996@live.com

[2]Girma Motuma (girmamoti1@gmail.com), [2]Shambel Boki (dshambelboki@yahoo.com), [2]Niguse Bekele (niguse_21@yahoo.com), [2]Tamiru kuru (tamiru.kuru@gmail.com), [2]Achalu Chimdi (achaluchimdi@yahoo.com)

Abstract

The study was conducted to characterize the Physicochemical properties of honey produced from underground nesting stingless bees (/Meliponula beccarii) in the Dandi and Meta Robi districts of the west Shewa zone, Ethiopia. A total of 24 honey samples with each 0.58–0.69-liter were collected through an excavation of natural nests and analyzed in the laboratory for various Physicochemical properties. The evaluated Physicochemical properties indicated an overall mean of 306.64±87.95 acidity mill./kg, 28.05±3.52 moisture content, 1.31±0.44 ms/cm electric conductivity, 3.29±0.16 pH, 0.89±1.14 9 mg/kg HMF, 0.63±0.24% mineral (ash), 9.39±4.26 glucose, 0.24±0.01g/100 sucrose, 10.81±0.4.95g/100g maltose and 16.57±2.55g/100g fructose. The differences between the two districts honey samples are significant (p> 0.05) in glucose value and considerable with acidity, moisture contents and pH values. The distinctive low HMF, high acidity and low pH values of the stingless bee honey of this study may represent freshness and good quality of the honey and indicative medicinal potential.

This study evaluated physicho-chemical properties of honey produced by ground nesting stingless bee (Meliponula beccarii) in two districts of West Shewa zone, Ethiopia and demonstrated the honeys have distinctive physicochemical properties from A.mellifera that may backup its uses as traditional medicine. Further detailed study on ground nesting and other stingless species honey medicinal values is recommended to provide scientific evidences.

Keywords: *Stingless bee, Physicochemical, honey, properties, moisture*

1. Introduction

Honey is a sweet, complex substance produced by bees through a series of processes before being stored in honeycombs or pots until it ripens (FAO, 2009). It is primarily composed of sugars, mainly monosaccharides (Cantarelli et al., 2008; Gela et al., 2021; Shugaba, 2012). Stingless bees, unlike their honeybee (Apis) counterparts, produce honey from the nectar of flowering plants and store it in pot-shaped structures made of wax cerumen (Kwapong et al., 2010).

Studies have shown that stingless bee honey and Apis honey possess distinct physicochemical properties. Stingless bee honey generally exhibits greater moisture content, higher acidity, lower sugar composition, and lower enzyme activity compared to Apis honey (Nordin et al., 2018). The chemical composition of stingless honey can also vary based on factors such as floral origin (Kahraman, et al. 2010), geographical origin, seasonal and environmental conditions, and handling techniques (Alvarez-Suarez et al., 2010; Silva et al., 2016).

Ethiopia is home to a diverse range of stingless bee species, including the ground-nesting Meliponula beccarii, whose honey, locally known as "damma daamu," is commonly harvested and used as traditional medicine (Mogessie, 1994; Gela et al., 2021; Wondmeneh et.al., 2020). However, the destructive harvesting methods employed may endanger the native stingless bee species and compromise the honey quality (Gela et al., 2021).

The Dandi and Meta Robi districts of Ethiopia are known for their stingless bee honey production and traditional medicinal uses. Despite this, the quality and authenticity of the honey from these regions remain unidentified. Therefore, the primary purpose of this study is to evaluate the physicochemical properties of honey from the native stingless bee (Meliponula beccarii) in these two districts.

2. Material and methods

Dandi and Meta Robi are districts located in the West Shewa Zone of the Oromia Regional State in Ethiopia. These two districts were purposefully selected from the West Shewa Zone for their high potential for stingless bee honey production and ease of access during data collection. According to the 2021/22 Annual Reports from the Agricultural Offices, Dandi district is located at 8°54'0.11"N latitude and 38°25'0.35"E longitude, and experiences minimum and maximum temperatures ranging from 9.3°C to 23.8°C, with annual rainfall between 750-1,300 mm. Meta Robi district is located at 9°19'0.6"N latitude and 38°09'0.6"E longitude and experiences minimum and maximum temperatures ranging from 20°C to 30°C, with annual rainfall between 750-1,300 mm (Figure 1).

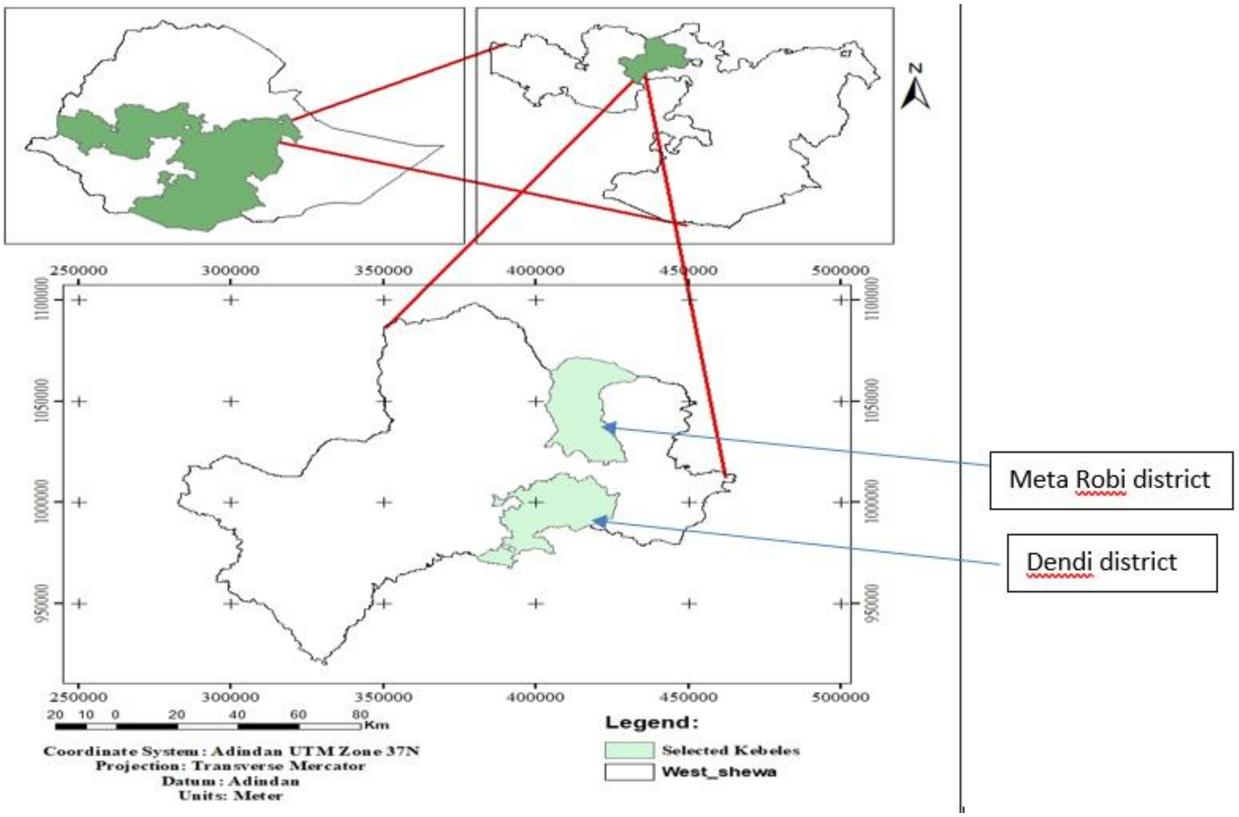

Figure 1: Map of study Areas

Please specify specific cooordinate location.

Careful excavations of 24 stingless bee nests (8 from Dandi district and 16 from Meta Robi district) were conducted. From each nest, fresh honey samples ranging from 0.25 to 0.5 liters were collected

using disposable 50 ml syringes. The collected honey samples were then stored in separate, airtight, and labeled jars (Figure 2) and properly refrigerated at 4°C until the dates of analysis.

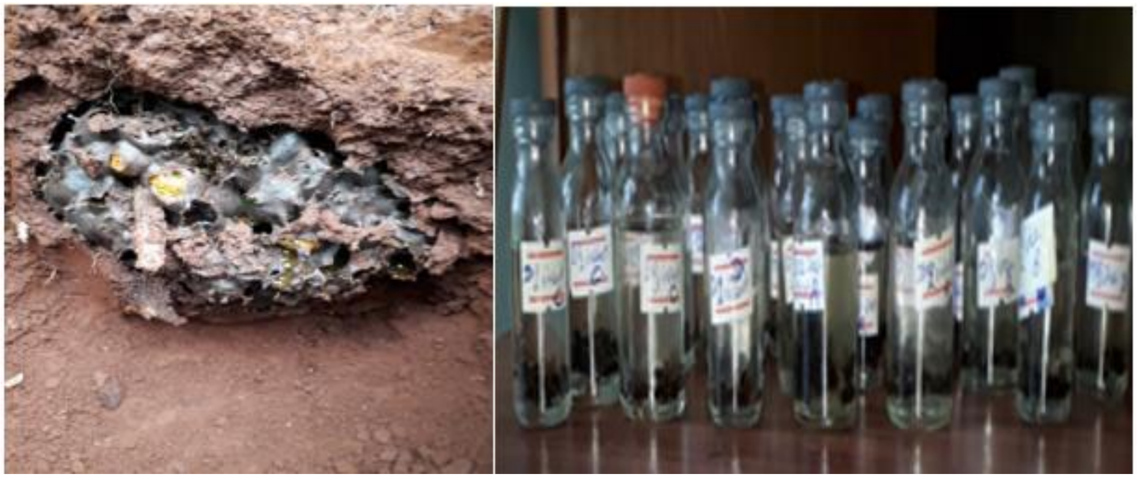

Figure 2: Stingless bee excavated nest and the collected honey

In addition, three honey samples were purchased from market to be used for quality comparison. The analysis of the collected honey samples was done at Ethiopian Conformity Assessment Enterprise (ECAE) in Addis Ababa in accordance of codex Alimentarius commission of the joint FAO/WHO food standards program (FAO/WHO Codex Alimentarius, 2019). The analyzed parameters included sugar content, moisture content, free acidity, pH, electrical conductivity, ash and HMF each with three replications. Determination of sugars was performed with a high-performance liquid chromatography (HPLC) equipped with a differential refractive index (DRI) detector (AOAC, 2000) with the separation using NH_2 column (4.6 x 250mm, ZORBAX NH_2) and a particle size diameter of 5µm with the column was kept at 30°C throughout the analysis. The mobile phase composition was 70% acetonitrile in water. The injection volumes of the samples were10µl, with a flow rate of 1.3ml/min. Retention times were identified using standards of peak. The honey samples were also spiked with standards in order to verify the identity of the chromatographic peaks. Triplicate injections were performed and for the peak quantification

average peak areas were used. Monosaccharaides (glucose, fructose) and disaccharides (sucrose, maltose, turanose) were used as standards to determine the sugar content of the honey samples. A 5g of honey sample was dissolved with 25% methanol in water solution. The solution was transferred to a 100ml volumetric flask. Solution was filtered through 0.45µm syringe and injected to HPLC. A standard curve was used to determine sugar content (fructose, glucose, sucrose, turanose and maltose) and the values were calculated as gram/100gram of honey sample.

A digital refractometer (Abbe refractometer, Leica Mark II Plus) thermo stated at 20 ^{O}C with the refractive index and regularly calibrated with distilled water was used (Bogdanov, 2009) to determine the Moisture content.

The honey samples were homogenized and the surface of the prism was covered evenly with the sample. After 2 minutes, the refractive index was read with refractometer. Each honey samples were measured triplicate and the average value was taken (Bogdanov, 2009). Free acidity and pH measurement according to Bogdanov (2009). A pH meter was used to measure the pH of a 10% (w/v) solution of honey prepared in deionized water. A 10g honey sample was dissolved in 75 ml of carbon dioxide-free water. It was stirred with the magnetic stirrer, then pH electrode immersed in the solution and the volume of titrate was recorded when titrated with 0.1M NaOH to pH 8.30. The reading was recorded and the free acidity was calculated using the following formula.

$$Free\ acidity(\text{milliequvalent}) = mL\ 0.1M\ NaOH * 10$$

Electrical conductivity was measured using conductivity meter (HANNA, HI 2550, EC/TDS/NaCl meter). 20% (w/v) honey solution was suspended in deionized water. Potassium Chloride solution (0.1M) was used for the cell constant. The electrical conductance of this solution was read in mS after the temperature has been equilibrated to 20˚C (International Honey Commission, 2009). 20g anhydrous honey was dissolved in distilled water and quantitatively transferred to a 100ml

volumetric flask. The electrical conductivity of the honey solution was calculated using the following formula:

$$SH = K * G$$

Where:

SH = electrical conductivity of the honey solution in mS.cm^{-1}

K = cell constant in cm^{-1}

G = conductance in mS

For temperatures above 20 °C: subtract 3.2 % of the value per °C

For temperatures below 20 °C: add 3.2 % of the value per °C

Ash content was determined according to the methods of International Honey Commission (2009). Accordingly, 5 g of honey was placed in combustion pots, which required preheating to darkness. Then, the samples incinerated at high temperature (550℃) in a burning muffle (THERMO CONCEPT, KLS 45/13, Germany) for 1 h. After cooling at room temperature, the obtained ash weighed and the proportion of ash (Ac) in g/100g honey was calculated using the following formula: -

$$AC = \frac{(m1 - m2)}{m3} * 100$$

Where:

m_3 = weight of honey

m_1 = weight of dish + ash,

m_2 = weight of dish

Hydroxymethylfurfural was identified as Standard solutions: 5-(hydroxymethyl-) furan-2-carbaldehyde (HMF) 1, 2, 5, 7 and 10 mg /L aqueous solution was prepared. The absorbance A of the prepared standard solution was determined using an UV spectrophotometer (CECIL, CE 7500,

7000 series) at 285 nm in 1 cm quartz cells with water in the blank cell. The concentration of the standard solutions was calculated from the literature values for molar absorptivity, ε=16830 or absorptivity.

$$\text{Concentration } (\frac{\text{mg}}{\text{L}}) = \frac{A}{1 * 133.57} * 1000$$

Where: A is the absorbance of the standard solution

HMF content was determined with HMIHC (International Honey Commission, 2009) with some modification. A high-performance liquid chromatography was used with an absorbance scale 285nm using DAD detector. 10g of prepared honey sample was dissolved with 50ml deionized water. The solution was filtered through a 0.45µm syringe filter and read on a HPLC (Agilent, 1260infinity) using a C18 column (3.0 x 250mm) (ZORBAX SB-C18) kept at 30°C throughout the analysis with a particle size diameter of 5µm. The mobile phase composition was 90% deionized water (1% acetic acid) with methanol. The injection volumes of the samples were 10µl, with a flow rate of 0.7ml/min (International Honey Commission, 2009). The HMF content of the samples was calculated by comparing the corresponding peak areas of the samples and those of the standard solutions taking into account the dilution. The collected data were systematically organized, coded, tabulated and analyzed using the statistical package for social science (SPSS) software version 20. One-way ANOVA was employed to compute the variations between the means of each variable in every studied parameter and 0.05% tests were considered significance.

3. Results and discussion

3.1. Acidity

In this study, the overall mean free acidity values of the stingless bee honey samples were 306.64 ± 87.95 meq/kg, ranging from 200-424.74 meq/kg (mean 338.79 ± 72.46) for the Dandi district, and 188.25-456.30 meq/kg (mean 274.49 ± 93.10) for the Meta Robi district. The mean free acid value for the purchased honey sample was 348.5 ± 12.0 meq/kg, and there was no significant difference ($P>0.05$) between the freshly collected honey samples from the two districts and the purchased sample (Table 1).

The mean acidity values in this study were well above the acceptable limit of international

Name of species and genus must italic

standards for Apis mellifera honey (<50 meq/kg) (Alimentarius, 2001), further confirming the acidic nature of the stingless bee honey (Nordin et al., 2018). The total acidity of honey can vary depending on the nectar pH, mineral (ash) content derived from the soil composition, or the plant species from which the final honey composition is derived (Ericco et al., 2011). Honey acidity is an important quality parameter, as it indicates the presence of organic acids, which contribute to the flavor and aroma of honey (Silva et al., 2016). Higher acidity values suggest a greater abundance of organic acids (Stramm, 2011). Honey acidity is influenced by enzymatic activity and low pH content (Stramm, 2011). Acidity values can also be useful for discriminating honeys of different floral origins, and honey adulterated with sugar syrup typically has very low acidity (<1) (Živkov Baloš et al., 2018).

3.2. Moisture content

Honey moisture content is an important quality parameter due to its impact on fermentation. The overall mean moisture content of the 24 stingless bee honey samples was 23.45-36.75% (mean 28.05±3.52%). The recorded moisture contents ranged from 25-36.75% (mean 29.34±4.02%) for

the Dandi district and 23.45-30.80% (mean 26.77±2.49%) for the Meta Robi district. The purchased honey samples had a moisture content of 34.20-36.90% (mean 35.40±0.79%), and there was no statistical difference ($P > 0.05$) between the honey samples from the two districts (Table 1).

This finding is consistent with previous studies. Gela et al. (2021) reported a moisture content of 25.1 - 35.0% (mean 29.6±1.4%) for honey samples of the same stingless bee species collected from four districts of Ethiopia. Teferi et al. (2022) also reported similar results.

Variations in moisture content among honey samples can be affected by factors such as adulteration, humidity, floral origin, soil type, collection period, and processing aspects (Vit et al., 2004; Norowi et al., 2010; Biluca et al., 2016; Michener, 2013; Sousa et al., 2016). The moisture contents noted in this study are lower than what was reported for stingless bee honeys from Mexico, Guatemala, and Venezuela (31.4% to 38.74%) (Patricia et al., 2016), but higher than the 13.86±1.06% reported for Melipona species in Nigeria (Nweze et al., 2017).

The standard moisture content limit for Apis mellifera honey is 20% or less, as per the Ethiopian (QSAE, 2005) and European (Commission, 2001) standards.

3.2. pH value

The mean pH values ranged from 3.0-3.42 (mean 3.24±0.13) for the Dandi district and 3.0-3.60 (mean 3.35±0.18) for the Meta Robi district. The purchased honey samples had a pH range of 3.27-3.35 (mean 3.31±0.023). The pH parameter showed no statistically significant differences ($p>0.05$) among all the sampled honeys (Table 1).

All honey samples showed low pH values, which is characteristic of stingless bee honeys (Nordin et al., 2018; Kwapong et al., 2010). This finding is consistent with previous studies. Gela et al. (2021) reported an overall pH range of 3.4-3.9 (mean 3.7±0.15) for stingless bee honey from four

districts in Ethiopia. Nweze et al. (2017) reported pH values of 3.75 and 4.21 for stingless bee honey (Melipona species) in Nigeria, and Souza et al. (2021) presented similar findings for the genus Melipona in Brazil.

The standard pH value for Apis mellifera honey is 3.42–6.10 (Tesfaye et al., 2016). The low pH value of Melipona honey has great significance for its extraction and storage, as it inhibits the occurrence and development of microorganisms (Alves et al., 2013; Silva et al., 2013) and reduces some infectious diseases (Mushonga et al., 2019; Oskouei & Najafi, 2013; Meo et al., 2017).

Table 1: Mean physicochemical properties of stingless bee honey samples (N = 27)

Values	**Parameters**										
	Acidity mill./kg	MC	EC ms/cm	HMF Mg/kg	pH	Ash	Glu	suc	Malt	Fruc	Tura
**Dandi district (N=8)**											
Min.	200.00	25.00	0.72	0.07	3.00	0.40	1.20	0.20	6.50	12.00	0.19
Max.	424.74	36.75	1.99	4.20	3.42	1.00	15.80	0.25	16.80	16.80	0.20
Mean	338.79	29.34	1.38	1.14	3.24	0.65	8.84	0.24	10.27	14.50	0.20
Std.D	72.46	4.02	0.48	1.53	0.13	0.25	5.29	0.01	3.98	1.54	0.00
**Meta Robi district(N=16)**											
Min.	188.25	23.45	0.75	0.06	3.00	0.36	5.80	0.22	5.20	15.90	0.19
Max.	456.30	30.80	1.85	1.30	3.60	0.95	13.40	0.26	20.00	20.00	0.20
Mean	274.49	26.77	1.24	0.64	3.35	0.61	9.94	0.24	11.35	18.63	0.20
Std.D	93.10	2.49	0.40	0.47	0.18	0.23	3.05	0.01	5.91	1.38	0.00
**Total**											
Min.	188.25	23.45	0.72	0.06	3.00	0.36	1.20	0.20	5.20	12.00	0.19

Max.	456.30	36.75	1.99	4.20	3.60	1.00	15.80	0.26	20.00	20.00	0.20
Mean	306.64	28.05	1.31	0.89	3.29	0.63	9.39	0.24	10.81	16.57	0.20
Std.D	87.95	3.52	0.44	1.14	0.16	0.24	4.26	0.01	4.95	2.55	0.00
P-Value	0.07	0.07	0.44	0.29	0.08	0.71	0.54	0.89	0.61	0.00	0.56
**Purchased honey (N=3)**											
Mean	348.5	35.40	1.50	0.28	3.31	0.64	9.53	0.25	7.8	13	0.20
Std.D	12.08	0.79	0.14	0.20	0.023	0.09	0.52	0.00	2.0	0.23	0.00
Max.	372.66	36.90	1.79	0.70	3.35	0.83	10.2	0.25	12	13.4	0.20
Min.	336.5	34.20	1.34	0.08	3.27	0.54	8.50	0.25	5.5	12.6	0.20
P-Value	0.078	0.001	0.005	0.01	0.30	0.005	0.20	0.03	0.01	0.01	

N=Number of samples, MC=Moisture content, HMF=hydroxyl methyl furfural, EC=Electric conductivity, pH=pH value, Std.D=Standard deviation, Min=minimum, Max=Maximum,

3.3. Mineral (ash) contents

The mineral (ash) content in honey is an indicator of the nectar botanical origin, location, bee species, and processing and handling methods (Biluca et al., 2016). In this study, the ash contents ranged from 0.40-1.0% (mean 0.65 ± 0.25%) for the Dandi district, 0.36-1.00% (mean 0.63±0.24%) for the Meta Robi district, and 0.54-0.83% (mean 0.64± 0.09%) for the purchased honey samples. There was no significant difference ($P < 0.05$) in the ash content among all the samples (Table 1).

The results of this study are consistent with the findings of Gela et al. (2021), who reported ash contents ranging from 0.21 to 0.57% for stingless bee honey. Although the overall average ash content of stingless bee honey is similar to the purchased honey from the market (0.5 ± 0.00%),

the results of the present study suggest that honey produced from the native stingless bee (M. beccarii) in Ethiopia is richer in mineral content.

This is significant, as the mineral content in honey can have important implications for its medicinal and nutritional value. The higher mineral content of the stingless bee honey from the Dandi and Meta Robi districts indicates that it may be a better choice for both medicinal and nutritional purposes, provided that the honey is properly standardized.

3.4. Sugar contents

According to this study honey samples, the mean sugar concentrations for glucose (9.39 ± 4.26 g/100g), sucrose (0.24 ± 0.01 g/100g), maltose (10.81 ± 4.95 g/100g), fructose (16.57 ± 2.55 g/100g), and turanose (0.20 ± 0.00 g/100g) were registered. The difference between the two districts was significant ($p<0.05$) only for fructose concentration. For the purchased honey samples, the mean concentrations were glucose (9.53 ± 0.52 g/100g), sucrose (0.25 ± 0.00 g/100g), and maltose (7.8 ± 2.0 g/100g). Except for maltose, the sugar concentrations recorded in this study were lower than those reported by Teferi D. et al. (2022) for stingless bee honey (fructose: 36.48 ± 0.54 g/100g, glucose: 27.67 ± 0.43 g/100g, sucrose: 1.24 ± 0.18 g/100g). Previous studies have also found that Melipona honeys contain small quantities of maltose (Bogdanov et al., 1996).

3.5. HMF

The Hydroxymethylfurfural (HMF) is used as an indicator of poor quality due to adulteration or poor handling like inappropriate storage and the lower the HMF, the freshness and good quality of the honey (Marchini et al., 2007). In this study, 0.06 and 4.2.0 values were recorded as minimum and maximum with overall mean of 0.89±1.14 mg/kg (

Table **1**). The highest HMF value was recorded in honey samples collected from Dandi district (4.20 mg/kg), and the difference in HMF value record in honey samples collected from both

districts is not significant (*p*>0.05). This finding disagrees with Gela et al., (2021) that stated high overall HMF range (11.2–22.4mg/kg) and the value reported by Teferi D. *et.al*, (2022) (6.58±0.36mg/kg) for pooled honey samples from 14 districts of Oromia, Ethiopia. Alimentarius, (2001) permits a maximum of 40 mg/kg for the HMF standard. The low HMF content including the purchased honey sample in this study indicates the freshness and good quality of the honeys.

3.6. Electrical conductivity

The overall mean calculated value of electrical conductivity of the 24 honey samples of stingless bees from the two study districts was 1.31±0.44 mS/cm, ranging from 0.72-1.99. The electrical conductivity values ranged from 0.72-1.99 mS/cm (mean 1.38±0.48) for the Dandi district samples, 0.75-1.85mS/cm (mean 1.24±0.40) for the Meta Robi district and 0.14-1.50mS/cm (mean 1.79±1.34) for purchased honey from (Table 2). The electrical conductivity difference of the two-district sampled honey is nto significant (*P*>0.05), suggesting similarities of flora compositions between the districts. The electrical conductivity in the current study is in line with Teferi D. *et.al*, (2022) and Gela et al., (2021) on stingless bee honey in Ethiopia. The conductivity values of the Melipona honeys varies from 0.32-0.44mS/cm (Bogdanov et al., 1996). The electrical conductivity values of the explored honey samples were within the range of allowable value of international standard value (i.e, greater than 0.8 mS/cm) (Alimentarius, 2001). The significance differences in electric conductivity of honey samples between the two locations suggests its usefulness for differentiating honeys of different botanical origin and purity (Živkov Baloš et al., 2018).

4. Conclusion

This study evaluated the physicochemical properties of honey produced by the ground-nesting stingless bee (Meliponula beccarii) in two districts of West Shewa Zone, Ethiopia. The results demonstrate that stingless bee honeys have distinctive physicochemical properties compared to

Apis mellifera honey. Except for glucose content, there were no significant differences in the analyzed physicochemical parameters between the two study districts.

However, the stingless bee honeys exhibited considerable differences in acidity, moisture content, and pH values. The moisture content and free acidity were higher than the standard limits for Apis mellifera honey. The distinctive low pH and high acidity of the stingless bee honey may represent its potential medicinal value. Additionally, the low HMF content indicates the freshness and good quality of the honeys.

Overall, this study highlights the unique physicochemical characteristics of stingless bee honey, which differentiate it from the more widely studied Apis mellifera honey. These findings have important implications for the standardization and utilization of stingless bee honey, particularly for medicinal and nutritional applications.

5. Recommendations

Building on the current study's insights, further comprehensive research is recommended to scientifically validate the traditional medicinal uses of native ground-nesting stingless bee (Meliponula beccarii) honey in Ethiopia. This future work should aim to elucidate the bioactive compounds and therapeutic potential, establish quality standards and testing methods, explore opportunities for local commercialization of medicinal products, and investigate sustainable stingless beekeeping practices. By pursuing this collaborative, multi-pronged approach, the unique medicinal and nutritional value of this specialized honey can be leveraged to benefit traditional and modern healthcare systems, while promoting the conservation of the native bee species.

REFERENCES

1. AOAC. (2000). Analytical Methods. *Toxicology and Industrial Health*, *15*(8), 715–717. https://doi.org/10.1177/074823379901500807
2. FAO (2009). *Bees and their role in forest livelihoods. A guide to the services provided by bees and the sustainable harvesting, processing, and marketing of their products. Food and Agriculture Organization of the United Nations, Rome.* 51–53.
3. Alvarez-Suarez, J. M., Tulipani, S., Díaz, D., Estevez, Y., Romandini, S., Giampieri, F., Damiani, E., Astolfi, P., Bompadre, S., & Battino, M. (2010). Antioxidant and antimicrobial capacity of several monofloral Cuban honeys and their correlation with color, polyphenol content and other chemical compounds. *Food and Chemical Toxicology*, *48*(8), 2490–2499. https://doi.org/https://doi.org/10.1016/j.fct.2010.06.021
4. Bekele Tesfaye, Desalegn Begna and Eshetu, M. (2016). *Evaluation of Physico-Chemical Properties of Honey Produced in Bale Natural Forest , Southeastern*. *2*, 21–27. https://doi.org/10.17352/2455-815X.000010
5. Biluca, F., Braghini, F., Gonzaga, L., Costa, A., & Fett, R. (2016). Physicochemical profiles, minerals and bioactive compounds of stingless bee honey (Meliponinae). *Journal of Food Composition and Analysis*, *50*. https://doi.org/10.1016/j.jfca.2016.05.007
6. Bogdanov, S. (2009). Harmonised Methods of the International IHC. *Bee Product Science*, *5*, 1–62.
7. Bogdanov, S., Vit, P., & Kilchenmann, V. (1996). Sugar profiles and conductivity of stingless bee honeys from Venezuela. *Apidologie*, *27*(6), 445–450. https://doi.org/10.1051/apido:19960602
8. Cantarelli, M. A., Pellerano, R., Marchevsky, E., & Camiña, J. (2008). Quality of honey

from Argentina: Study of chemical composition and trace elements. *The Journal of Argentine Chemical Society*, *96*, 33–41.

9. FAO/WHO Codex Alimentarius. (2019). *Procedural manual, twenty-seventh edition*.
10. Gela, A., Hora, Z. A., Kebebe, D., & Gebresilassie, A. (2021). Physico-chemical characteristics of honey produced by stingless bees (Meliponula beccarii) from West Showa zone of Oromia Region, Ethiopia. *Heliyon*, *7*(1). https://doi.org/10.1016/j.heliyon.2020.e05875
11. hilmi, M., & Martin hilmi. (2005). *The Marketing of bee products*.
12. Kahraman. (2010). Physico-chemical properties in honey from different regions of Turkey. .." *FOOD CHEMISTRY*, *123*(1), 41–44.
13. Kwapong, P., Aidoo, K., Combey, R., & Karikari, A. (2010). Stingless bees: importance, management and utilisation. A training manual for stingless beekeeping. In *Social Insects* (Issue August 2016).
14. Maia-Silva, C., Hrncir, M., Imperatriz-Fonseca, V. L., & Schorkopf, D. L. P. (2016). Stingless bees (Melipona subnitida) adjust brood production rather than foraging activity in response to changes in pollen stores. *Journal of Comparative Physiology A: Neuroethology, Sensory, Neural, and Behavioral Physiology*, *202*(9–10), 723–732. https://doi.org/10.1007/s00359-016-1095-y
15. Michener, C. (2013). The Meliponini. In *Pot-Honey: A Legacy of Stingless Bees* (pp. 3–17). https://doi.org/10.1007/978-1-4614-4960-7_1
16. Mogessie, A. (1994). The in vitro antibacterial activity of 'Tazma mar' honey produced by sting less bee. *Ethiop J Health Dev*, *8*(2), 109–117.
17. Mushonga, B., Hategekimana, L., Habarugira, G., Kandiwa, E., Samkange, A., & Ernest

Segwagwe, B. V. (2019). Characterization of the Beekeeping Value Chain: Challenges, Perceptions, Limitations, and Opportunities for Beekeepers in Kayonza District, Rwanda. *Advances in Agriculture*, *2019*. https://doi.org/10.1155/2019/5982931

18. Nordin, A., Sainik, N. Q. A. V., Chowdhury, S. R., Saim, A. Bin, & Idrus, R. B. H. (2018). Physicochemical properties of stingless bee honey from around the globe: A comprehensive review. *Journal of Food Composition and Analysis*, *73*, 91–102. https://doi.org/https://doi.org/10.1016/j.jfca.2018.06.002
19. Norowi, M., Sajap, A., Fahimie, M., Suri, R., & Jaapar, M. (2010). *Conservation and sustainable utilization of stingless bees for pollination services in agricultural ecosystems in Malaysia*.
20. Nweze, J., Okafor, J., Nweze, E., & Nweze, E. (2017). Evaluation of physicochemical and antioxidant properties of two stingless bee honeys: A comparison with Apis mellifera honey from Nsukka, Nigeria. *BMC Research Notes*, *2017*, 566. https://doi.org/10.1186/s13104-017-2884-2
21. Oskouei, T., Najafi, M. (2013). Traditional and modern uses of natural honey in human diseases: a review. *Iran. J. Basic Med. Sci.*, *16 731*(6), 731.
22. Patricia, RMP, S., Vargas J, C., M, A., & F, M. (2016). Chemical Composition of Ecuadorian Commercial Pot-Honeys: Trigona fuscipennis “Abeja De Tierra”, Melipona mimetica “Bermejo” and Scaptotrigona ederi “Catiana.” *Pharmaceutica Analytica Acta*, *7*(12). https://doi.org/10.4172/2153-2435.1000525
23. Pauly, A. (2013). Belgian Journal of Entomology. *Belgian Journal of Entomology*, *69*(December), 1–19.
24. Quality and Standards Authority of Ethiopia (QSAE). (2005). *Honey Specification:*

Ethiopian Standard, ES 1202. Addis Ababa, Ethiopia.

25. Shugaba, A. (2012). Analysis of Biochemical Composition of Honey Samples from North-East Nigeria. *Biochemistry & Analytical Biochemistry*, *2*(3). https://doi.org/10.4172/2161-1009.1000139
26. Sousa, J. M., de Souza, E. L., Marques, G., Meireles, B., de Magalhães Cordeiro, Â. T., Gullón, B., Pintado, M. M., & Magnani, M. (2016). Polyphenolic profile and antioxidant and antibacterial activities of monofloral honeys produced by Meliponini in the Brazilian semiarid region. *Food Research International*, *84*, 61–68. https://doi.org/https://doi.org/10.1016/j.foodres.2016.03.012
27. Souza, E. C. A., Menezes, C., & Flach, A. (2021). Stingless bee honey (Hymenoptera, Apidae, Meliponini): a review of quality control, chemical profile, and biological potential. *Apidologie*, *52*(1), 113–132. https://doi.org/10.1007/s13592-020-00802-0
28. Stramm, K. M. (2011). *Composition and Quality of Honey Bee Jandaira (Melipona Subnitida), Storage Effects and Comparison with Apis mellifera Honey.*
29. Teferi Damto, D. K. and M. (2022). *Physico-chemical and microbiological characteristics of honey produced by stingless bees*.
30. Vit, P., Medina, M., & Eunice Enríquez, M. (2004). Quality standards for medicinal uses of Meliponinae honey in Guatemala, Mexico and Venezuela. *Bee World*, *85*(1), 2–5. https://doi.org/10.1080/0005772X.2004.11099603
31. Wondmeneh Jemberie, Worku Negash, Kassahun Alemu, Alemu Tarekegn, M. B. & N. R. (2020). Stingless bee Meliponula Cockerell (Hymenoptera: Apidae: Meliponini) ground nest architecture and traditional knowledge on the use of honey in the Amhara Region, Northwest Ethiopia. *Israel Journal of Entomology*, *50*(March 2021), 14–17.

https://doi.org/10.5281/zenodo.4588315

32. Živkov Baloš, M., Popov, N., Vidaković, S., Ljubojević Pelić, D., Pelić, M., Mihaljev, Ž., & Jakšić, S. (2018). Electrical conductivity and acidity of honey. *Archives of Veterinary Medicine*, *11*(1), 91–101. https://doi.org/10.46784/e-avm.v11i1.20
