# Supplementary material for: Physicochemical properties of stingless bees (Meliponula beccarii) honey in Dandi and Meta Robi districts of West Shewa zone, Ethiopia
Source: PLoS One. 2024 Dec 12;19(12):e0311725. doi: 10.1371/journal.pone.0311725 (PMC11637301; doi:10.1371/journal.pone.0311725)
Supplement: Attachment — Submitted filename: PONE-D-24-28380_R2_reviewer.pdf [file pone.0311725.s006.pdf]

PLOS ONE

Physicochemicalproperties of stingless bees (meliponula beccarii) honey in Dandi and Meta Robi districts of West Shewa zone, Ethiopia

--Manuscript Draft--

**Manuscript Number:**	PONE-D-24-28380R2
**Article Type:**	Research Article
**Full Title:**	Physicochemicalproperties of stingless bees (meliponula beccarii) honey in Dandi and Meta Robi districts of West Shewa zone, Ethiopia
**Short Title:**	Physicochemical Profile of Ground-Nesting Stingless Bee Honey in Ethiopia
**Corresponding Author:**	Desalegn Begna, Ph.D<br>Policy Studies Institute, Ethiopia<br>Addis Ababa, ETHIOPIA
**Keywords:**	Stingless bee, Physicochemical, honey, properties, moisture
**Abstract:**	The study was conducted to characterize the physicochemical properties of honey produced from underground nesting stingless bees (Meliponula beccarii) in the Dandi and Meta Robi districts of the West Shewa zone, Ethiopia. A total of 27 honey samples, including 24 collected through careful investigation and excavation of natural nests and 3 purchased from the market, were analyzed in the laboratory. The evaluated physicochemical properties showed an overall mean of 306.64±87.95 meq./kg free acidity, 28.05±3.52% moisture content, 1.31±0.44 mS/cm electrical conductivity, 3.29±0.16 pH, 0.89±1.14 mg/kg HMF, 0.63±0.24% mineral (ash), 9.39±4.26% glucose, 0.24±0.01 g/100g sucrose, 10.81±4.95 g/100g maltose, and 16.57±2.55 g/100g fructose. The differences between the two district's honey samples were significant (p > 0.05) for fructose value and considerable for acidity, moisture content, and pH values. The honey samples purchased from the market showed similar physicochemical properties to the honey from the feral nests, with a mean of 314.33±88.72 meq./kg free acidity, 27.73±2.52% moisture content, 1.43±0.41 mS/cm electrical conductivity, 3.26±0.13 pH, 0.95±1.23 mg/kg HMF, 0.59±0.19% mineral (ash), 10.11±4.11% glucose, 0.25±0.02 g/100g sucrose, 11.23±4.52 g/100g maltose, and 16.33±2.41 g/100g fructose. The high acidity and low pH in the Meliponula beccarii honey appear unusually elevated compared to other stingless bee honey and the Codex Alimentarius standards for Apis honey, suggesting increased fermentation that can originate from the bee species, plant and geographical origins, improper handling, and high moisture content. The study found that the stingless bee honey from the study areas had distinctive low HMF, high acidity, and low pH values, which may indicate the honey's potential traditional medicinal properties. Further detailed studies on ground-nesting and other stingless species' honey medicinal values are recommended to provide scientific evidence.
**Order of Authors:**	Desalegn Begna, Ph.D
	Girma Motuma, Msc
	Shambel Boki, Msc
	Niguse Bekele, PhD
	Tammiru Kuru, Msc
	Achalu Chimdi, PhD
**Response to Reviewers:**	Response to Reviewers<br><br>PONE-D-24-28380R1<br><br>Physicochemical properties of stingless bees (meliponula beccarii) honey in Dandi and Meta Robi districts of West Shewa zone, Ethiopia<br><br>PLOS ONE<br>____

Powered by Editorial Manager® and ProduXion Manager® from Aries Systems Corporation

6. Review Comments to the Author

Please use the space provided to explain your answers to the questions above. You may also include additional comments for the author, including concerns about dual publication, research ethics, or publication ethics. (Please upload your review as an attachment if it exceeds 20,000 characters)
Reviewer #1: MINOR CORRECTIONS: Please highlight these changes in RED/BLUE in your corrected manuscript

a) Introduction Section:
Please include a brief mention of Meliponula beccarii as an important stingless bee species in Ethiopia, with appropriate references. Highlight that this species is commonly found in the Dandi and Meta Robi districts of the West Shewa zone, Ethiopia.
Meliponula beccarii, a species of stingless bee, is commonly found in Ethiopia and is well known for its honey production as well as its contributions to local biodiversity and pollination. It is particularly prevalent in the Dandi and Meta Robi districts of the West Shewa zone, where it plays a vital role in the ecosystem.

b) Results and Discussion Section:

(i) Figure 2: The current image of the water bottle is not ideal. We need to clearly see the color of the honey (green/yellow/dark). Please describe the color more clearly and replace the image with a better one.
The image of the water bottle removed

(ii) Please be cautious with the phrase “to indicate medicinal value of honey.” It would be more appropriate to say: “indicate the medicinal and nutritional value of honey.” Kindly check and revise this. Corrected accordingly (on page 10 of the revised manuscript)

(iii) The discussion on the sugar profile needs further elaboration and additional citations (Please compare with other findings the data from Stingless Bee as well). The comparison with the Codex Alimentarius standard is very appropriate maintain as it is, BUT should also include comparisons with stingless bee honey from other regions like Southeast Asia (Malaysia, Indonesia, Thailand) and South America- Brazil/Venezuela etc..
I recommend comparing your results with the standards for stingless bee honey from other countries. Please refer to existing standards for stingless bee honey in these regions (IF ANY).

Others are OK!
Thank you for your constructive comments on our manuscript. We greatly appreciate your input, as it has helped us enhance the quality of our work.
In addition to addressing your suggestions, we have included comparisons for each physicochemical parameter with stingless bee honey from other countries in Southeast Asia and South America in the table and body of the revised manuscript. We believe this additional context strengthens our findings.

Reviewer #2:
Abstract
1. Check maltose content result because it is extremely high .compare with other findings
Previous studies has reported maltose levels in stingless bee honey generally range from lower values, typically around 1% to 5% (22,23). However, the high maltose content in our stingless bee honey may be due to several factors like specific floral sources can significantly influence sugar composition, with certain plants containing higher maltose concentrations (22), fermentation processes within the nest may also increase maltose levels if honey is stored for extended periods (23), the foraging behavior of stingless bees that could lead to the preferential collection of nectar from maltose-rich plants and environmental factors, such as soil composition and climate, that may further impact floral diversity and nectar quality, resulting in elevated maltose levels (53), processing and storage methods might alter sugar profiles, with prolonged storage or heat affecting composition (24), variability among stingless bee species

Powered by Editorial Manager® and ProduXion Manager® from Aries Systems Corporation

	(23).<br><br>2.Again I inform the authors that high acidity (Free acidity) is not related with quality and medicinal properties .High acidity of stingless bee related to fermentation rate of this honey because stingless bee honey contain high moisture and alcohol is easily formed in it .that is way free acidity is high.<br>High quality and medicinal properties of stingless bee is related to strong pH and high antioxidant properties.<br>Higher free acidity in honey can enhance flavor complexity, inhibit microbial growth to extend shelf life (Tan et al., 2021), and provide potential health benefits, including antibacterial properties from organic acids (Tey et al., 2022).<br><br>3. The result of turanose was not presented in abstract:<br>Included in the abstract of the revised manuscript<br>Materials and methods: In the M&M, the analysis of turanose was similar as fructose, glucose, and sucrose and already stated as “A standard curve was used to determine sugar content (fructose, glucose, sucrose, turanose, and maltose) and the values were calculated as gram/100gram of honey sample”<br><br>Results and Discussion<br>4. Please free acidity more acceptable than acidity on sub heading (corrected accordingly)<br>5. The authors stated that free acidity was extremely high in this study and also mentioned this is because of different species and agro ecologies. But since different study was conducted in West Shoa as the author stated in introduction, why you don’t compare you’re finding with those study conducted in same area and same country. The study lack literature discussion in Ethiopia.<br>Or high acidity because of your sample impurity? Please discuss carefully on the finding. I didn’t see improvement.<br>Reports from Ethiopia has been included and discussed well with plausible justifications added for why free acidity was high in our honey sample in the manuscript<br>6. The line and paragraph spacing and font color is not uniform in all paragraph (corrected accordingly)<br>7. Correct sub heading of moisture content (corrected accordingly)<br>8. Font color and size of sub heading should be improved ((corrected accordingly)<br>9. In abstract the authors stated elevated pH value but in results and discussion parts, the finding of the study is similar with other finding conducted in Ethiopia. Revise abstract or discussion parts. The abstract part revised.<br>10. The arrangement of the paragraph is week<br>11. For me discussion of sugar profile is not enough? Needs to be revised again by comparing the finding of the study with other similar findings. Well discussed this time in the revised manuscript<br>12. Under HMF paragraph please correct the paragraph (corrected)<br>13. Why fresh collected and marketed ones was not compared in finding. It is better if both types of samples was compared parallel. Both types of samples were compared parallel at every parameter<br><br>Conclusion<br>14.“This study evaluated This study evaluated “. Please remove redundant sentence (Removed accordingly)<br>15.” This study evaluated the physicochemical properties of honey produced by the ground-nesting stingless bee (Meliponula beccarii) in Ethiopia” , by collecting sample from two woreda of Oromia region we can say this study represent Ethiopia. Please revise this one again. (Revised)<br>165 .revise the conclusion based on your fining especially on fresh and marketed honey sample variability (revised)<br>176. lack of consistent formatting of reference: We utilized the Mendeley desktop reference manager to format our references according to the Vancouver style outlined in the PLOS author guidelines. We believe this will ensure consistent formatting throughout the reference section. Additionally, we have made adjustments to enhance the presentation and engage the reviewer more effectively.
**Additional Information:**	

Powered by Editorial Manager® and ProduXion Manager® from Aries Systems Corporation

Question	Response
**Financial Disclosure**<br><br>Enter a financial disclosure statement that describes the sources of funding for the work included in this submission. Review the submission guidelines for detailed requirements. View published research articles from *PLOS ONE* for specific examples.<br><br>This statement is required for submission and **will appear in the published article** if the submission is accepted. Please make sure it is accurate.<br><br>**Funded studies**<br>Enter a statement with the following details:<br>• Initials of the authors who received each award<br>• Grant numbers awarded to each author<br>• The full name of each funder<br>• URL of each funder website<br>• Did the sponsors or funders play any role in the study design, data collection and analysis, decision to publish, or preparation of the manuscript?<br><br>Did you receive funding for this work?	The author(s) received no specific funding for this work.
**Competing Interests**<br><br>Use the instructions below to enter a competing interest statement for this submission. On behalf of all authors, disclose any competing interests that could be perceived to bias this work—acknowledging all financial support and any other relevant financial or non-financial competing interests.<br><br>This statement is required for submission and **will appear in the published article** if the submission is accepted. Please make sure it is accurate and that any funding sources listed in your Funding Information later in the submission form are also	The authors have declared that no competing interests exist

declared in your Financial Disclosure statement.<br><br>View published research articles from *PLOS ONE* for specific examples.<br><br>**NO authors have competing interests**<br><br>Enter: *The authors have declared that no competing interests exist.*<br><br>**Authors with competing interests**<br><br>Enter competing interest details beginning with this statement:<br><br>*I have read the journal's policy and the authors of this manuscript have the following competing interests: [insert competing interests here]*<br><br>* typeset	
**Ethics Statement**<br><br>Enter an ethics statement for this submission. This statement is required if the study involved:<br><br>• Human participants<br>• Human specimens or tissue<br>• Vertebrate animals or cephalopods<br>• Vertebrate embryos or tissues<br>• Field research<br><br>Write "N/A" if the submission does not require an ethics statement.<br><br>General guidance is provided below. Consult the submission guidelines for detailed instructions. **Make sure that all information entered here is included in the Methods section of the manuscript.**	N/A

**Format for specific study types**<br><br>**Human Subject Research (involving human participants and/or tissue)**<br>• Give the name of the institutional review board or ethics committee that approved the study<br>• Include the approval number and/or a statement indicating approval of this research<br>• Indicate the form of consent obtained (written/oral) or the reason that consent was not obtained (e.g. the data were analyzed anonymously)<br><br>**Animal Research (involving vertebrate animals, embryos or tissues)**<br>• Provide the name of the Institutional Animal Care and Use Committee (IACUC) or other relevant ethics board that reviewed the study protocol, and indicate whether they approved this research or granted a formal waiver of ethical approval<br>• Include an approval number if one was obtained<br>• If the study involved *non-human primates*, add *additional details* about animal welfare and steps taken to ameliorate suffering<br>• If anesthesia, euthanasia, or any kind of animal sacrifice is part of the study, include briefly which substances and/or methods were applied<br><br>**Field Research**<br><br>Include the following details if this study involves the collection of plant, animal, or other materials from a natural setting:<br>• Field permit number<br>• Name of the institution or relevant body that granted permission	
**Data Availability**<br><br>Authors are required to make all data underlying the findings described fully available, without restriction, and from the time of publication. PLOS allows rare exceptions to address legal and ethical concerns. See the PLOS Data Policy and FAQ for detailed information.	Yes - all data are fully available without restriction

A Data Availability Statement describing where the data can be found is required at submission. Your answers to this question constitute the Data Availability Statement and **will be published in the article**, if accepted.<br><br>**Important:** Stating 'data available on request from the author' is not sufficient. If your data are only available upon request, select 'No' for the first question and explain your exceptional situation in the text box.<br><br>Do the authors confirm that all data underlying the findings described in their manuscript are fully available without restriction?	
**Describe where the data may be found in full sentences. If you are copying our sample text, replace any instances of XXX with the appropriate details.**<br><br>• If the data are **held or will be held in a public repository**, include URLs, accession numbers or DOIs. If this information will only be available after acceptance, indicate this by ticking the box below. For example: *All XXX files are available from the XXX database (accession number(s) XXX, XXX.).*<br>• If the data are all contained **within the manuscript and/or Supporting Information files**, enter the following: *All relevant data are within the manuscript and its Supporting Information files.*<br>• If neither of these applies but you are able to provide **details of access elsewhere**, with or without limitations, please do so. For example:<br><br>*Data cannot be shared publicly because of [XXX]. Data are available from the XXX Institutional Data Access / Ethics Committee (contact via XXX) for researchers who meet the criteria for access to confidential data.*<br><br>*The data underlying the results presented in the study are available from (include the name of the third party*	All relevant data is presented in the manuscript. However, additional row data in Excel has been uploaded as supplemental information. This time, all personal information has been removed or anonymized as per the editor's comment

and contact information or URL).<br>• This text is appropriate if the data are owned by a third party and authors do not have permission to share the data.<br><br>* typeset	
Additional data availability information:	

Manuscript Click here to access/download;Manuscript;Manuscript.docx

Physicochemical properties of stingless bees (meliponula beccarii) honey in Dandi and Meta Robi districts of West Shewa zone, Ethiopia

Desalegn Begna[1*], Girma Motuma[2¶], Shambel Boki[3¶], Niguse Bekele[4&], Tamiru kuru[5&], Achalu Chimdi[6&]

[1]Agriculture and Rural Development Center, Policy Institute Study Institute, Addis Ababa, Ethiopia

[2] Department of Animal Science, College of Agriculture and Veterinary Science Ambo University Mamo Mezemir Campus, Guder, Ethiopia

[3]Department of Animal Science in the College of Agriculture and Veterinary science Ambo University, Mamo Mezemir Campus, Guder, Ethiopia

[4]Department of Natural Resource Management, Ambo University, Mamo Mezemir Campus, Guder, Ethiopia

[5]Department of Natural Resource Management, Ambo University, Mamo Mezemir Campus, Guder, Ethiopia

[6]Department of Soil Sciences, Ambo University Mamo Mezemir Campus, Guder, Ethiopia

[*]Correspondence author: Desalegn Begna; dbegna67@gmail.com (DB)

[¶] These authors contributed equally to this work.

[&] These authors also contributed equally to this work

Abstract

The study was conducted to characterize the physicochemical properties of honey produced from underground nesting stingless bees (Meliponula beccarii) in the Dandi and Meta Robi districts of the West Shewa zone, Ethiopia. A total of 27 honey samples, including 24 samples collected through careful investigation and excavation of natural nests and 3 samples purchased from the market, were analyzed in the laboratory. The evaluated physicochemical properties showed an overall mean of 306.64±87.95 meq./kg free acidity, 28.05±3.52% moisture content, 1.31±0.44 mS/cm electrical conductivity, 3.29±0.16 pH, 0.89±1.14 mg/kg HMF, 0.63±0.24% mineral (ash), 9.39±4.26% glucose, 0.24±0.01 g/100g sucrose, 10.81±4.95 g/100g maltose, and 16.57±2.55 g/100g fructose, turanose (0.20 ± 0.00 g/100g). The differences between the two district's honey samples were significant ($p > 0.05$) for fructose value and considerable for free acidity, moisture content, and pH values. The honey samples purchased from the market showed similar physicochemical properties to the honey from the feral nests, with a mean of 314.33±88.72 meq./kg free acidity, 27.73±2.52% moisture content, 1.43±0.41 mS/cm electrical conductivity, 3.26±0.13 pH, 0.95±1.23 mg/kg HMF, 0.59±0.19% mineral (ash), 10.11±4.11% glucose, 0.25±0.02 g/100g sucrose, 11.23±4.52 g/100g maltose, and 16.33±2.41 g/100g fructose. The study found that the stingless bee honey from the study areas had distinctive low HMF, high free acidity, and low pH values, which may indicate the honey's potential medicinal properties. The high free acidity in the Meliponula beccarii honey appear unusually elevated compared to other stingless bee honey and the Codex Alimentarius standards for Apis honey, suggesting increased fermentation that can originate from the bee species, plant and geographical origins, improper handling, and high moisture content. This study demonstrated that the honey in the study areas has distinctive physicochemical properties from A. mellifera-produced honey, which may support its traditional medicinal uses. Further detailed studies on ground-nesting and other stingless species' honey medicinal values are recommended to provide scientific evidence...

Keywords: *Stingless bee, Physicochemical, honey, properties, moisture*

Introduction

Honey is a sweet, complex substance produced by bees through a series of processes before being stored in honeycombs or pots until it ripens (1). It is primarily composed of sugars, mainly monosaccharides (2–4). Stingless bees, unlike their honeybee (*Apis Mellifera*) counterparts, produce honey from the nectar of flowering plants and store it in pot-shaped structures made of wax cerumen (5).

Studies have shown that stingless bee honey and *Apis* honey possess distinct physicochemical properties. Stingless bee honey generally exhibits greater moisture content, higher acidity, lower sugar composition, and lower enzyme activity compared to Apis honey (6,7). The chemical composition of stingless honey can also vary based on factors such as floral origin (6,8,9), geographical origin, seasonal and environmental conditions, and handling techniques (10–13).

Ethiopia is home to diverse stingless bee species, including the ground-nesting *Meliponula beccarii*, whose honey, locally known as "*damma daamu*," is commonly harvested and used as traditional medicine (3,14,15). *Meliponula beccarii*, a stingless bee species, is commonly found in Ethiopia and is well known for its honey production as well as its contributions to local biodiversity and pollination. It is particularly prevalent in the Dandi and Meta Robi districts of the West Shewa zone, where it plays a vital role in the ecosystem (16–18). However, the destructive harvesting(3) methods employed may endanger the native stingless bee species and compromise the honey quality (3). Characterization of the physicochemical properties of stingless bee honey in some districts of West Shewa like Cheliya, Jeldu, Wolmera, and Tokekutaye has been studied (3). The Dandi and Meta Robi districts of Ethiopia are known for their stingless bee honey production and traditional medicinal uses. Despite this, the quality and authenticity of the stingless bee honey reported from other districts of west Shewa, information is lacking for Dandi and Meta Robi

districts. Therefore, the primary purpose of this study is to evaluate the physicochemical properties of honey from the native stingless bee (*Meliponula beccarii*) in these two districts.

Material and methods

The study areas

Dandi and Meta Robi are districts located in the West Shewa Zone of the Oromia Regional State in Ethiopia. These two districts were purposefully selected from the West Shewa Zone due to their high potential for stingless bee honey production and ease of access during data collection. According to the 2021/22 Annual Reports from the Agricultural Offices, Dandi district is located at 8°54'0.11"N latitude and 38°25'0.35"E longitude, and experiences minimum and maximum temperatures ranging from 9.3°C to 23.8°C, with annual rainfall between 750-1,300 mm. Meta Robi district is located at 9°19'0.6"N latitude and 38°09'0.6"E longitude and experiences minimum and maximum temperatures ranging from 20°C to 30°C, with annual rainfall between 750-1,300 mm). The locations were purposively chosen for the study representing the high potential areas of stingless bee, *Meliponula beccarii,* and with diverse floral composition.

Sample acquisitions

Careful investigation and excavations of 24 wild stingless bee nests (*Meliponula beccarii*) (8 from Dandi district and 16 from the Meta Robi district) were conducted. From each nest, fresh honey samples ranging from 0.25 to 0.5 liters were collected using disposable 50 ml syringes. In addition, three honey samples were purchased from the market to be used for comparison, making the total honey samples 27. The collected honey samples were then stored in separate, airtight, and labeled jars (Fig 1) and properly refrigerated at 4°C until the dates of the analysis.

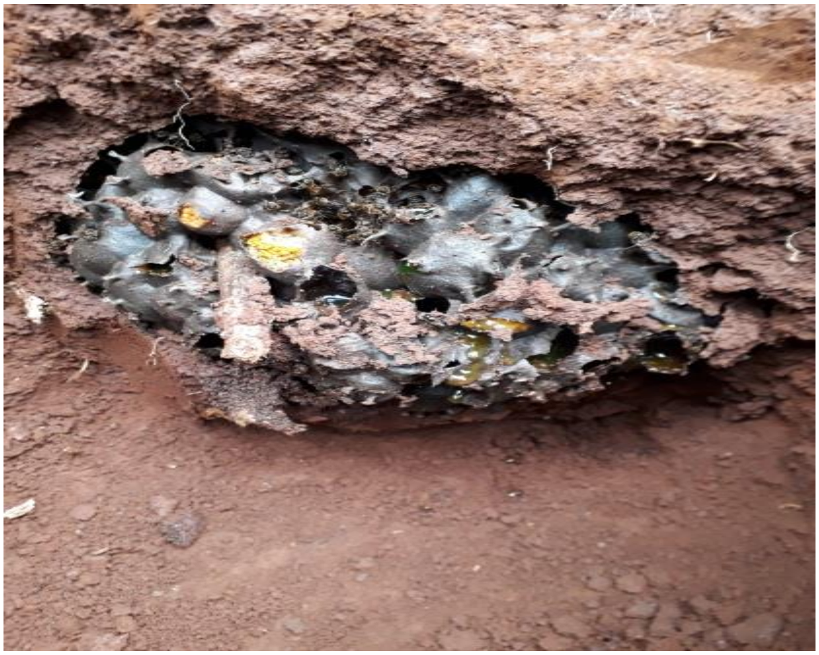

Fig **1**: Excavated feral stingless bee nest

Methodologies and protocols of the honey analysis

The analysis of the collected honey samples was done at Ethiopian Conformity Assessment Enterprise (ECAE) in Addis Ababa in accordance of the codex Alimentarius commission of the joint FAO/WHO food standards program (19). The analyzed parameters included sugar content, moisture content, free acidity, pH, electrical conductivity, ash, and HMF each with three replications. Determination of sugars was performed with high-performance liquid chromatography (HPLC) equipped with a differential refractive index (DRI) detector (20) with the separation using NH_2 column (4.6 x 250mm, ZORBAX NH_2) and a particle size diameter of 5µm with the column was kept at 30°C throughout the analysis. The mobile phase composition was 70% acetonitrile in water. The injection volumes of the samples were10µl, with a flow rate of 1.3ml/min. Retention times were identified using standards of peak. The honey samples were also

spiked with standards in order to verify the identity of the chromatographic peaks. Triplicate injections were performed and for the peak quantification average peak areas were used. Monosaccharaides (glucose, fructose) and disaccharides (sucrose, maltose, turanose) were used as standards to determine the sugar content of the honey samples. A 5g of honey sample was dissolved with 25% methanol in water solution. The solution was transferred to a 100ml volumetric flask. Solution was filtered through 0.45µm syringe and injected to HPLC. A standard curve was used to determine sugar content (fructose, glucose, sucrose, turanose and maltose) and the values were calculated as gram/100gram of honey sample.

A digital refractometer (Abbe refractometer, Leica Mark II Plus) thermo stated at 20 ^{O}C with the refractive index and regularly calibrated with distilled water was used (21) to determine the moisture content.

The honey samples were homogenized and the surface of the prism was covered evenly with the sample. After 2 minutes, the refractive index was read with refractometer. Each honey samples were measured triplicate and the average value was taken (21). Free acidity and pH measurement according to (21). A pH meter was used to measure the pH of a 10% (w/v) solution of honey prepared in deionized water. A 10g honey sample was dissolved in 75 ml of carbon dioxide-free water. It was stirred with the magnetic stirrer, then pH electrode immersed in the solution and the volume of titrate was recorded when titrated with 0.1M NaOH to pH 8.30. The reading was recorded and the free acidity was calculated using the following formula.

$$Free\ acidity(\text{milliequvalent}) = mL\ 0.1M\ NaOH * 10$$

Electrical conductivity was measured using a conductivity meter (HANNA, HI 2550, EC/TDS/NaCl meter). 20% (w/v) honey solution was suspended in deionized water. Potassium Chloride solution (0.1M) was used for the cell constant. The electrical conductance of this solution

was read in mS after the temperature has been equilibrated to 20˚C (International Honey Commission, 2009). 20g anhydrous honey was dissolved in distilled water and quantitatively transferred to a 100ml volumetric flask. The electrical conductivity of the honey solution was calculated using the following formula:

$$SH = K * G$$

Where:

SH = electrical conductivity of the honey solution in mS.cm^{-}1

K = cell constant in cm^{-}1

G = conductance in mS

For temperatures above 20 °C: subtract 3.2 % of the value per °C

For temperatures below 20 °C: add 3.2 % of the value per °C

Ash content was determined according to the methods of International Honey Commission (2009). Accordingly, 5 g of honey was placed in combustion pots, which required preheating to darkness. Then, the samples incinerated at high temperature (550℃) in a burning muffle (THERMO CONCEPT, KLS 45/13, Germany) for 1 h. After cooling at room temperature, the obtained ash weighed and the proportion of ash (Ac) in g/100g honey was calculated using the following formula: -

$$AC = \frac{(m1 - m2)}{m3} * 100$$

Where:

m_3 = weight of honey

m_1 = weight of dish + ash,

m_2 = weight of dish

Hydroxymethylfurfural was identified as Standard solution: 5-(hydroxymethyl-) furan-2-carbaldehyde (HMF) 1, 2, 5, 7 and 10 mg /L aqueous solution was prepared. The absorbance A of the prepared standard solution was determined using an UV spectrophotometer (CECIL, CE 7500, 7000 series) at 285 nm in 1 cm quartz cells with water in the blank cell. The concentration of the standard solutions was calculated from the literature values for molar absorptivity, ε=16830 or absorptivity.

$$\text{Concentration}\ (\frac{\text{mg}}{\text{L}}) = \frac{A}{1 * 133.57} * 1000$$

Where: A is the absorbance of the standard solution

HMF content was determined with HMIHC (International Honey Commission, 2009) with some modifications. A high-performance liquid chromatography was used with an absorbance scale 285nm using DAD detector. 10g of prepared honey sample was dissolved with 50ml deionized water. The solution was filtered through a 0.45µm syringe filter and read on a HPLC (Agilent, 1260infinity) using a C18 column (3.0 x 250mm) (ZORBAX SB-C18) kept at 30°C throughout the analysis with a particle size diameter of 5µm. The mobile phase composition was 90% deionized water (1% acetic acid) with methanol. The injection volumes of the samples were 10µl, with a flow rate of 0.7ml/min (International Honey Commission, 2009). The HMF content of the samples was calculated by comparing the corresponding peak areas of the samples and those of the standard solutions taking into account the dilution. The collected data were systematically organized, coded, tabulated, and analyzed using the Statistical Package for Social Science (SPSS) software version 20. One-way ANOVA was employed to compute the variations between the means of each variable in every studied parameter and 0.05% tests were considered significance.

Results and discussion

Free acidity

In this study, the overall mean free acidity values of the stingless bee honey samples were 306.64 ± 87.95 meq/kg, ranging from 200-424.74 meq/kg (mean 338.79 ± 72.46) for the Dandi district, and 188.25-456.30 meq/kg (mean 274.49 ± 93.10) for the Meta Robi district. The mean free acid value for the purchased honey sample was 348.5 ± 12.0 meq/kg, and there was no significant difference (P>0.05) between the freshly collected honey samples from the two districts and the purchased sample (Table 1).

The mean free acidity values in this study were well above the acceptable limit of international standards for *Apis mellifera* honey (<50 meq/kg) (19), further confirming the acidic nature of the stingless bee honey (6). Reports from Ethiopia indicate mean free acidity levels of 92.39 ± 4.45 meq/kg (18) and 7.3 ± 0.36 meq/kg (3) for stingless bee honey samples from various parts of the country, both of which are significantly lower than our findings. Our value is also notably higher than the free acidity of stingless bee honey from Southeast Asia (40 to 60 meq/kg) and South America (50 to 70 meq/kg). The elevated free acidity of *Meliponula beccarii* honey in our samples is likely attributed to a combination of diverse floral sources with high organic acid content (22), specific environmental conditions that promote fermentation (23), and unique soil characteristics influencing nectar composition (24), enzymatic activity and low pH content (25).

Honey free acidity is an important quality parameter, as it indicates the presence of organic acids, which contribute to the flavor and aroma of honey (11). Acidity values can also be useful for discriminating honeys of different floral origins, and honey adulterated with sugar syrup typically has very low free acidity (<1) (26). The acidity levels in stingless bee honeys can vary significantly based on factors like bee species, geographical origin, and other variables. The free acidity values

were reported 26.5 to 66.4 meq/kg for *Melipona scutellaris* honey (Souza et al., 2006), 29.7 ± 4.4 meq/kg for *Plebeia saiqui* honey, and 42.7 ± 4.2 meq/kg for *Tetragonisca angustula* honey (Vit et al., 1998), and 22.4 to 34.6 mEq/kg for *Melipona beecheii* honey (Ruiz-Argueso and Rodriguez-Navarro, 1973). The *Meliponula beccarii* honey studied appears to have unusually high free acidity and low pH compared to stingless bee honey from Brazil (Melipona scutellaris), Venezuela (Plebeia saiqui, Tetragonisca angustula), and Mexico (Melipona beecheii), which typically range from 22.4 to 66.4 meq/kg. This suggests the high free acidity may be due to increased fermentation, potentially stemming from factors like differences in bee species and geographical origin (27), improper handling, or high moisture content (28), which requires further investigation. The higher free acidity in honey is directly linked to its enhanced medicinal potential, and honey with higher free acidity (lower pH) exhibits stronger antimicrobial activity against pathogens (Kadariya et al., 2020; Olaitan et al., 2022; Sherlock et al., 2010), greater antioxidant capacity (7,10), improved anti-inflammatory and wound healing properties (29), and effective antibiofilm activity that can disrupt and prevent microbial biofilms (30,31), collectively highlighting the direct relationship between the high free acidity in honey and its potential for medicinal and nutritional valueof honey.

Moisture content

Honey moisture content is an important quality parameter due to its impact on fermentation. The overall moisture content of the honey samples was reported to be between 23.45% and 36.75%.(mean 28.05±3.52%). The recorded moisture contents ranged from 25-36.75% (mean 29.34±4.02%) for the Dandi district and 23.45-30.80% (mean 26.77±2.49%) for the Meta Robi district. The purchased honey samples had a moisture content of 34.20-36.90% (mean 35.40±0.79%), and there was no statistical difference ($P > 0.05$) between the honey samples from the two districts (Table 1). The moisture content of freshly collected honey was lower than

the in marketed honey and this lower moisture content helps prevent fermentation, indicating better quality preservation of the freshly collected honey sample (23).

This finding is consistent with previous studies (3)reported a moisture content of 25.1 - 35.0% (mean 29.6±1.4%) for honey samples of the same stingless bee species collected from four districts of Ethiopia (32) also reported similar results. Studies have shown that stingless bee honey from Southeast Asia (Malaysia) typically has a moisture content ranging from 30% to 35% (24) and in contrast, honey from South America has been observed to have slightly lower moisture content, between 28% and 30% and Brazilian stingless bee honey exhibited moisture levels around 28% (22).

Variations in moisture content among honey samples can be affected by factors such as adulteration, humidity, floral origin, soil type, collection period, and processing aspects (12,33–35). The moisture contents noted in this study are lower than what was reported for stingless bee honeys from Mexico, Guatemala, and Venezuela (31.4% to 38.74%) (36), but higher than the 13.86±1.06% reported for Melipona species in Nigeria (37).

The standard moisture content limit for *Apis mellifera* honey is 20% or less, as per the Ethiopian (26) and European (21)standards.

Table 1: Mean physicochemical properties of stingless bee honey samples (N = 27)

Values	Parameters							
	Free Acidity meq/kg	MC %	EC mS/cm	HMF Mg/kg	pH	Ash (g)	Glu (g)	suc[illegible]
**Dandi district (N=8)**								
Min.	200.00	25.00	0.72	0.07	3.00	0.40	1.20	0[illegible]
Max.	424.74	36.75	1.99	4.20	3.42	1.00	15.80	0[illegible]
Mean± std	338.79±72.46	29.34±4.04	1.38±0.48	1.14±1.53	3.24±0.13	0.65±0.25	8.84±5.29	0.24[illegible]
**Meta Robi district(N=16)**								
Min.	188.25	23.45	0.75	0.06	3.00	0.36	5.80	0[illegible]
Max.	456.30	30.80	1.85	1.30	3.60	0.95	13.40	0[illegible]
Mean± std	274.49± 93.1	26.77±2.49	1.24±0.4	0.64±0.46	3.35±0.18	0.61±0.23	9.94±3.05	0.24[illegible]
**Total (Dendi and meta Robi)**								
Min.	188.25	23.45	0.72	0.06	3.00	0.36	1.20	0[illegible]
Max.	456.30	36.75	1.99	4.20	3.60	1.00	15.80	0[illegible]
Mean ± std	306.64±87.95	28.05±3.52	1.31±0.44	0.89±1.14	3.29±0.16	0.63±0.24	9.39±4.26	0.24[illegible]
P-Value	0.07	0.07	0.44	0.29	0.08	0.71	0.54	0[illegible]
**Purchased honey (N=3)**								
Mean± std	348.5±12.08	35.40±0.79	1.50±0.14	0.28±0.2	3.31±0.02	0.64±0.09	9.53±0.52	0.25[illegible]
Max.	372.66	36.90	1.79	0.70	3.35	0.83	10.2	0[illegible]
Min.	336.5	34.20	1.34	0.08	3.27	0.54	8.50	0[illegible]
P-Value	0.078	0.001	0.005	0.01	0.30	0.005	0.20	0[illegible]
Codex Alimentarius Standard for Honey (CODEX STAN 12-1981, Rev.2-2001)	50	20	0.8	40	3.4 - 6.1	<0.6	N/A	
Southeast Asia	40-60	30-35	0.5-1.0	5-10	3.5-4.5	0.1-0.3	25-35	1[illegible]
South America	50-70	28-30	0.3-0.8	10-20	3.0-4.0	0.2-0.5	25-30	1[illegible]

N=Number of samples, MC=Moisture content, HMF=hydroxyl methyl furfural, EC=Electric conductivity, pH=pH value, Std.D=Standard deviation, Min=minimum, Max=Maximum, MC (Moisture content), HMF (hydroxymethylfurfural,) EC (Electrical Conductivity), Tura (Turanose,) Glu (Glucose), Suc (sucrose), Fruc (Fructose), Malt (Maltose)

The pH value

The mean pH values ranged from 3.0-3.42 (mean 3.24±0.13) for the Dandi district and 3.0-3.60 (mean 3.35±0.18) for the Meta Robi district. The purchased honey samples had a pH range of 3.27-3.35 (mean 3.31±0.023). The pH parameter showed no statistically significant differences ($p>0.05$) among all the sampled honeys (Table 1).

All honey samples showed low pH values, which is characteristic of stingless bee honeys (5,6). This finding is consistent with previous studies(3) reported an overall pH range of 3.4-3.9 (mean 3.7±0.15) for stingless bee honey from four districts in Ethiopia (37) reported pH values of 3.75 and 4.21 for stingless bee honey (Melipona species) in Nigeria, and (40) presented similar findings for the genus *Melipona* in Brazil(22). The pH of honey from Southeast Asia typically varies from 3.5 to 4.5, as reported by (24) and honey from South America exhibits a pH range of 3.0 to 4.0 according to findings by (22), indicating the *Meliponula beccarii* honey from Ethiopia has a pH, which falls within the lower spectrum of both Southeast Asian and South American honeys. Lower pH may enhance the honey's preservation and antibacterial properties, contributing to its quality (23).

The standard pH value for *Apis mellifera* honey is 3.42–6.10 (41,42). The low pH value of Melipona honey has great significance for its extraction and storage, as it inhibits the occurrence and development of microorganisms (43,44) and reduces some infectious diseases (45,46).

Ash contents

In this study, the ash contents ranged from 0.40-1.0 (mean 0.65 ± 0.25g) for the Dandi district, 0.36-1g (mean 0.63±0.24g) for the Meta Robi district, and 0.54-0.83g (mean 0.64± 0.09g) for the purchased honey samples. There was no significant difference ($P < 0.05$) in the ash content among all the samples (Table 1).

The mineral composition of honey could be influenced by the geographical and botanical origin of the flora, the type and activity of the bees, the extraction technique, and the storage conditions (47). The results of this study are consistent with the findings of (3), who reported ash contents ranging from 0.21 to 0.57% for stingless bee honey. Although the overall average ash content of stingless bee honey is similar to the purchased honey from the market (0.5 ± 0.00%), the results of the present study suggest that honey produced from the stingless bee (*M. beccarii*) in Ethiopia is richer in mineral content.

This is significant, as the mineral content in honey can have important implications for its for various aspects; used as a marker to identify the geographical origin of the honey, allow for honey traceability and authentication (48); and indicates the predominant floral sources that the bees have visited, as different plant species accumulate varying levels of minerals in their nectar (49); used to assess the quality and authenticity of honey, as adulteration or improper processing can alter the natural mineral profile (50); and it is relevant to the honey's nutritional value, as it provides information about the bioavailability of essential minerals, such as potassium, calcium, and magnesium (51). It is also established that the ash content of stingless bee honey varies by region, with Southeast Asia ranging from 0.1% to 0.3% (24) and South America between 0.2% and 0.5% (22). The lower ash eontents for *Meliponula beccarii* honey from Ethiopia suggests that Ethiopian honey may have a reduced mineral profile, likely due to the specific flora and soil characteristics in the region and such differences highlight the impact of local biodiversity on honey composition and its potential nutritional value (22–24).

Sugar contents

The mean sugar concentrations reported were glucose (9.39 ± 4.26 g/100g), sucrose (0.24 ± 0.01 g/100g), maltose (10.81 ± 4.95 g/100g), fructose (16.57 ± 2.55 g/100g), and turanose (0.20

± 0.00 g/100g). Interestingly, the only significant difference ($p<0.05$) between the two districts was observed for the fructose concentration. Comparing these results to a previous study, the sugar concentrations recorded were generally lower are fructose (36.48 ± 0.54 g/100g), glucose (27.67 ± 0.43 g/100g), and sucrose (1.24 ± 0.18 g/100g) in stingless bee honey samples (32). The Melipona honeys, typically contain small quantities of maltose (52). The sugar composition reveals that Southeast Asian honey has fructose levels ranging from 35% to 45% and glucose levels from 25% to 35%. South American honey also shows high fructose levels of 40% to 50%, which aligns closely with the Southeast Asian findings. The balance of these sugars influences the taste, sweetness, and overall quality of the honey (22,23).

Previous studies has reported maltose levels in stingless bee honey generally range from lower values, typically around 1% to 5% (22,23). However, the high maltose content in our stingless bee honey may be due to several factors like specific floral sources can significantly influence sugar composition, with certain plants containing higher maltose concentrations (22), fermentation processes within the nest may also increase maltose levels if honey is stored for extended periods (23), the foraging behavior of stingless bees that could lead to the preferential collection of nectar from maltose-rich plants and environmental factors, such as soil composition and climate, that may further impact floral diversity and nectar quality, resulting in elevated maltose levels (53), processing and storage methods might alter sugar profiles, with prolonged storage or heat affecting composition (24), variability among stingless bee species (23).

These differences in sugar composition could be attributed to various factors, such as the geographical and botanical origins of the honey, the species of stingless bees involved, and the extraction and processing methods employed (27,48,50,51,54).

Hydroxymethylfurfural (HMF)

In this study, 0.06 and 4.2 values were recorded as minimum and maximum with an overall mean of 0.89±1.14 mg/kg (

). The highest HMF value was recorded in honey samples collected from the Dandi district (4.20 mg/kg), and the difference in HMF value recorded in honey samples collected from both districts is not significant (*p*>0.05). This finding disagrees with (3) that stated a high overall HMF range (11.2–22.4mg/kg) and the value reported by (32) (6.58±0.36mg/kg) for pooled honey samples from 14 districts of Oromia, Ethiopia. (19) permits a maximum of 40 mg/kg for the HMF standard. The low HMF content including the purchased honey sample in this study indicates the freshness and good quality of the honey (55–57). The Hydroxymethylfurfural (HMF) is used as an indicator of poor quality due to adulteration or poor handling like inappropriate storage and the lower the HMF, the freshness and good quality of the honey (9). HMF levels in Southeast Asian honey are reported at 5 to 10 mg/kg, while in South America, they can range from 10 to 20 mg/kg, indicating Ethiopian honey has an HMF content within the acceptable limits for freshness (23,53).

Electrical conductivity

The overall mean calculated value of electrical conductivity of the 24 honey samples of stingless bees from the two study districts was 1.31±0.44 mS/cm, ranging from 0.72-1.99. The electrical conductivity values ranged from 0.72-1.99 mS/cm (mean 1.38±0.48) for the Dandi district samples, 0.75-1.85mS/cm (mean 1.24±0.40) for the Meta Robi district and 0.14-1.50mS/cm (mean 1.79±1.34) for purchased honey (Table 1). The electrical conductivity difference of the two-district sampled honey is not significant (*P*>0.05), suggesting similarities of flora compositions between the districts. The electrical conductivity in the current study is in line with (32) and (3) on stingless bee honey in Ethiopia. The conductivity values of the Melipona

honeys vary from 0.32-0.44mS/cm (58). The electrical conductivity values of the explored honey samples were within the range of allowable value of international standard value (i.e, greater than 0.8 mS/cm) (19). The significance differences in electric conductivity of honey samples between the two locations suggest its usefulness for differentiating honey of different botanical origins and purity (26).

The unique findings from the data are that the stingless bee (*Meliponula beccarii*) honey from the Dandi and Meta Robi districts of Ethiopia exhibits high acidity, low pH, low HMF, and a distinctive sugar profile, differentiating it from other places studied stingless bee honey (3) indicating potential medicinal properties.

Conclusion

This study assessed the physicochemical properties of honey produced by the ground-nesting stingless bee (Meliponula beccarii) in Ethiopia and compared these properties to those of stingless bee honey of same species from Ethiopia, stingless bee honey from Southeast Asia, and South America, as well as to the Codex Alimentarius standards for *Apis mellifera* honey and showed both similarities and differences.

Furthermore, the study highlights the distinctive physicochemical profile of stingless bee honey, characterized by significantly higher free acidity (306.64 meq/kg vs. 50 meq/kg), elevated moisture content (28.05% vs. 20%), lower pH (3.29 vs. 3.4-6.1), and higher electrical conductivity (1.31 mS/cm vs. 0.8 mS/cm). Its hydroxymethylfurfural (HMF) content (0.89 mg/kg) is well within Codex standards (40 mg/kg), indicating freshness and quality. The unique low pH and high acidity contribute to potential medicinal benefits. A key finding of this study is the variability between freshly collected and marketed honey samples, with fresh honey consistently demonstrating superior quality indicators compared to marketed counterparts, which often have higher acidity and moisture levels underscoring the need for specific quality standards and regulations for stingless bee honey to ensure its authenticity and efficacy.

While the promising characteristics of stingless bee honey position it as a valuable natural remedy, the study also acknowledges limitations, including a restricted scope, lack of seasonal variation analysis, absence of bioactive compound profiling, and insufficient comparative studies with other honey types. Addressing these gaps in future research will enhance our understanding of stingless bee honey's full potential for medicinal and nutritional applications, supporting its standardization and utilization across various sectors.

Recommendations

Based on the findings of this study, establishing specific quality standards and regulations for stingless bee (Meliponula beccarii) honey, given its distinctive physicochemical profile that differs from the Codex Alimentarius standards *for Apis mellifera honey*, and develop certification and labeling schemes to ensure its authenticity and quality; explore the potential medicinal applications of stingless bee honey, leveraging its unique properties such as high acidity, low pH, and low HMF content, and conduct further research to fully characterize its bioactive compounds and therapeutic potential; investigate the potential nutritional benefits. While promoting the diversification of honey sources; support the conservation and sustainable management of stingless bee populations, and develop training and extension programs to educate local communities on their importance; and encourage interdisciplinary collaboration between researchers, beneficiaries, policymakers, and end-users to foster the development and utilization of stingless bee honey as a valuable natural resource.

Acknowledgment

The authors wish to express their gratitude to Ambo University, Mamo Mezemir Campus, Department of Animal Science, for allowing this work to proceed and for its unwavering support throughout the data collection process.

References

1. FAO. The state of food and agriculture. Soil Sci [Internet]. 2009;95(4):11. Available from: https://www.fao.org/3/i0100e/i0100e.pdf
2. Cantarelli MA, Pellerano R, Marchevsky E, Camiña J. Quality of honey from Argentina: study of chemical composition and trace elements. J Argentine Chem Soc. 2008 Dec;96:33–41.
3. Gela A, Hora ZA, Kebebe D, Gebresilassie A. Physico-chemical characteristics of honey produced by stingless bees (Meliponula beccarii) from West Showa zone of Oromia Region, Ethiopia. Heliyon. 2021;7(1).
4. Shugaba A. Analysis of biochemical composition of honey samples from North-East Nigeria. Biochem Anal Biochem. 2012;2(3).
5. Kwapong P, Aidoo K, Combey R, Karikari A. Stingless bees: importance, management and utilization. A training manual for stingless beekeeping. Accra North: Uni Max Macmillan; 2010.
6. Nordin A, Sainik NQAV, Chowdhury SR, Saim A Bin, Idrus RBH. Physicochemical properties of stingless bee honey from around the globe: a comprehensive review. J Food Compos Anal [Internet]. 2018;73:91–102. Available from: https://www.sciencedirect.com/science/article/pii/S0889157518302953
7. Sadia H, Anjum AA, Yasmeen R, Javed F, Zafar MA, Khan IU, Inam-ul-Haq M. Comparative analysis of antioxidant potential and antimicrobial activity of different honey samples. Vet World. 2022;15(4):1034–41.
8. Kahraman T, Buyukunal SK, Vural A, Altunatmaz SS. Physico-chemical properties in honey from different regions of Turkey. Food Chem. 2010;123:41–44.

9. L.C. Marchini, A.C.d.C.C. Moreti, I.P. Otsuk G d. SS. Physicochemical composition of Apis mellifera honey samples from São Paulo state, Brazil. 30(7):1653–7.
10. Alvarez-Suarez JM, Tulipani S, Díaz D, Estevez Y, Romandini S, Giampieri F, et al. Antioxidant and antimicrobial capacity of several monofloral Cuban honeys and their correlation with color, polyphenol content and other chemical compounds. Food Chem Toxicol [Internet]. 2010;48(8):2490–9. Available from: https://www.sciencedirect.com/science/article/pii/S0278691510003959
11. Maia-Silva C, Hrncir M, Imperatriz-Fonseca VL, Schorkopf DLP. Stingless bees (Melipona subnitida) adjust brood production rather than foraging activity in response to changes in pollen stores. J Comp Physiol A Neuroethol Sensory, Neural, Behav Physiol. 2016;202(9–10):723–32.
12. Norowi M, Sajap H, Rosliza ASJ, Mohd Fahimie AJ, Suri R. Conservation and sustainable utilization of stingless bees for pollination services in agricultural ecosystems in Malaysia. In: Proceeding of International Seminar of Enhancement of Functional Biodiversity Relevant to Sustainable Production in ASPAC – In Association With MARCO. 2010.
13. Vit P, Roubik DW, Pedro SRM. Pot-honey: a legacy of stingless bees. Pot-Honey: A Legacy of Stingless Bees. 2012. 1–654 p.
14. Mogessie A. The in vitro antibacterial activity of ‘Tazma mar’ honey produced by stingless bee. Ethiop J Heal Dev. 1994;8(2):109–17.
15. Wondmeneh Jemberie, Worku Negash, Kassahun Alemu, Alemu Tarekegn MB, NR. Stingless bee Meliponula Cockerell (Hymenoptera: Apidae: Meliponini) ground nest architecture and traditional knowledge on the use of honey in the Amhara Region, Northwest Ethiopia. Isr J Entomol. 2020;50(March 2021):14–7.

16. Getachew T, Tesfaye G, Tadesse A. The ecology of stingless bees in Ethiopia. J Apic Res. 2021;60(4):345–58.

17. Tesfaye G, Tadesse A. Distribution and conservation of Meliponula beccarii in Ethiopia. Ethiop J Biol Sci. 2022;18(2):123–34.

18. Damto T, Kebebe D, Gemeda M. Physicochemical properties and microbial qualities of honey produced by a stingless bee (Meliponula beccarii L.) in the Oromia region, Ethiopia. Cogent Food Agric [Internet]. 2023;9(1). Available from: https://doi.org/10.1080/23311932.2023.2258784

19. FAO/WHO Codex Alimentarius. Procedural manual, twenty-seventh edition. 2019. 254 p.

20. AOAC. Analytical Methods. Toxicol Ind Health. 2000;15(8):715–7.

21. Bogdanov S. Harmonised Methods of the International IHC. Bee Prod Sci. 2009;(5):1–62.

22. Cavalcante IG, Santos LS, Freitas BM. Physicochemical properties of stingless bee honey from Brazil. J Apic Res. 2020;59(2):182–90.

23. Silveira FA, Oliveira JA, Ferreira JR. Comparative analysis of stingless bee honey from Venezuela and Brazil. Ethiop J Biol Sci. 2021;18(1):67–75.

24. Tey SL, Tan KW, Lee CY. Quality assessment of stingless bee honey from Southeast Asia. Food Chem. 2022;370:131045.

25. Stramm KM. Composition and quality of honey bee Jandaira (Melipona subnitida), storage effects, and comparison with Apis mellifera honey. 2011.

26. Živkov Baloš M, Popov N, Vidaković S, Ljubojević Pelić D, Pelić M, Mihaljev Ž, et al. Electrical conductivity and acidity of honey. Arch Vet Med. 2018;11(1):91–101.

27. Souza R, Roubik D, Barth E. Composition of stingless bee honey: setting quality standards. Interciencia. 2006;31(12).

28. Al-Tikrity WS, Benton AW, Hillmann RC, Clarke WW. The relationship between the amount of HMF in honey and its fermentation. Am Bee J. 1989;129(7):478–81.
29. Almasaudi SB, El-Shitany NA, Abbas AT, Abdel-Dayem UA, Ali SS, Al Jaouni SK, Harakeh S. Antioxidant, anti-inflammatory, and antiulcer potential of manuka honey against gastric ulcer in rats. Oxid Med Cell Longev. 2017.
30. Majtan J, Bohova J, Horniackova M, Klaudiny J, Majtan V. Anti-biofilm effects of honey against wound pathogens Proteus mirabilis and Enterobacter cloacae. Phyther Res. 2014;28(1):69–75.
31. Kilty SJ, Duval M, Chan FT, Ferris W, Slinger R. Methylglyoxal: active agent of manuka honey in vitro activity against bacterial biofilms. Int Forum Allergy Rhinol. 2011;1(5):348–50.
32. Teferi Damto DK, Meseret. Physico-chemical and microbiological characteristics of honey produced by stingless bees. 2022.
33. Vit P, Oddo L, Marano M, Mejias E. Venezuelan stingless bee honey characterized by multivariate analysis of physicochemical properties. Apidologie. 1998;(29):377–89.
34. Biluca FC, Della Betta F, de Oliveira GP, Pereira LM, Gonzaga LV, Costa ACO. 5-HMF and carbohydrates content in stingless bee (Meliponinae spp.) honey by CE before and after thermal treatment. Food Chem. 2014;159:244–9.
35. Biluca FC, Braghini F, Gonzaga LV, Oliveira Costa AC, Fett R. Physicochemical profiles, minerals and bioactive compounds of stingless bee honey (Meliponinae). J Food Compos Anal. 2016;50:61–9.
36. Vit P, Roubik DW, Pedro SRM. Pot-honey: a legacy of stingless bees. Pot-Honey: A Legacy of Stingless Bees. 2012. 1–654 p.

37. Nweze JA, Okafor J, Nweze EI. Evaluation of physicochemical and antioxidant properties of two stingless bee honeys: a comparison with Apis mellifera honey from Nsukka, Nigeria. BMC Res Notes. 2017;10:566.

38. Quality and Standards Authority of Ethiopia (QSAE). ET 1202: Honey - Specification [Internet]. 2005. Available from: https://archive.org/search?query=subject%3A%22public.resource.org%22

39. Quality and Standards Authority of Ethiopia (QSAE). Quality and Standards Authority of Ethiopia (QSAE) Honey Specification: Ethiopian Standard, ES 1202. 2005.

40. Souza ECA, Menezes C, Flach A. Stingless bee honey (Hymenoptera, Apidae, Meliponini): a review of quality control, chemical profile, and biological potential. Apidologie. 2021;52(1):113–32.

41. Bekele Tesfaye, Deaslegn Begna ME. Evaluation of physicochemical properties of honey produced in Bale Natural Forest, Southeastern Ethiopia. Int J Agric Sci Food Technol. 2016;1(2).

42. Bogdanov S, Jurendic T, Sieber R, Gallmann P. Honey for nutrition and health: a review. J Am Coll Nutr. 2009 Jan 1;27:677–89.

43. Silva JMC, Barbosa LCF, Leal IR TM. The Caatinga: understanding the challenges. In: Silva JMC, Leal IR, Tabarelli M (eds) Caatinga: the largest tropical dry forest region in South America. Springer, Cham. 2017;3–19.

44. Alves DA, Imperatriz-Fonseca VL, Francoy TM, Santos-Filho PS, Bil-len JW. Successful maintenance of a stingless bee population despite a severe genetic bottleneck. Conserv Genet. 2011;2:647–58.

45. Mushonga B, Hategekimana L, Habarugira G, Kandiwa E, Samkange A, Segwagwe BV. Characterization of the beekeeping value chain: challenges, perceptions, limitations, and opportunities for beekeepers in Kayonza District, Rwanda. Adv Agric. 2019;2019.
46. Meo SA, Al-Asiri SA, Mahesar AL. Role of honey in modern medicine. Saudi J Biol Sci. 2017;24(5):975–8.
47. Nnadi PC. Determination and comparative evaluation of mineral content of honey obtained from Rivers State University, Forestry Arboretum and Port Harcourt Local Market. J Appl Sci Environ Manag. 2020;24(9):1593–7.
48. Anklam E. A review of the analytical methods to determine the geographical and botanical origin of honey. Food Chem. 1998;63:549–62.
49. Machado De-Melo AA, Almeida-Muradian LB de, Sancho MT, Pascual-Maté A. Composición y propiedades de la miel de Apis mellifera: una revisión. J Apic Res. 2018;57(1):5–37.
50. Downey G, Fouratier V, Kelly JD. A review of the analytical methods to determine the geographical and botanical origin of honey. J Agric Food Chem. 2003;51(2):347–54.
51. Karabagias IK, Karabournioti S, Karabagias VK, Badeka AV. Palynological, physico-chemical and bioactivity parameters determination of a less common Greek honeydew honey: "dryomelo." Food Control [Internet]. 2020;109:106940. Available from: https://www.sciencedirect.com/science/article/pii/S0956713519305298
52. Bogdanov S, D'Arcy BR, Mossel B, Marcazzan GL. Honey quality and international regulatory standards: review by the International Honey International honey standards are specified in a European Honey. Bee World. 1999;80(2):61–9.
53. Tan KW, Lim HP, Lee CY. Physicochemical properties of honey from stingless bees in Malaysia. J Apic Res. 2021;60(1):45–54.

54. Couvillon MJ, Wenseleers T, Imperatriz-Fonseca VL, Nogueira-Neto P, Ratnieks FLW. A comparative study in stingless bees (Meliponini) demonstrates that nest entrance size predicts traffic and defensivity. J Evol Biol. 2008;21(1):194–201.

55. Zulkhairi Amin FA, Sabri S, Mohammad SM, Ismail M, Chan KW, Ismail N, et al.Therapeutic properties of stingless bee honey in comparison with European bee honey. Adv Pharmacol Sci. 2018;2018.

56. Kiprono SJ, Mengich G, Kosgei J, Mutai C, Kimoloi S. Ethnomedicinal uses of stingless bee honey among native communities of Baringo County, Kenya. Sci African [Internet]. 2022;17:e01297. Available from: https://doi.org/10.1016/j.sciaf.2022.e01297

57. Sabella JK, Gladys M, Bartholomew NO, Charles M, Sammy K. Therapeutic uses of stingless bee honey by traditional medicine practitioners in Baringo County, Kenya. J Pharmacogn Phyther. 2022;14(3):27–36.

58. Bogdanov S, Vit P, Kilchenmann V. Sugar profiles and conductivity of stingless bee honeys from Venezuela. Apidologie. 1996;27(6):445–50.

Click here to access/download
Supporting Information
EXCEL HONEY LAB_SUP_DATA.xlsx

Physicochemical properties of stingless bees (*meliponula beccarii*) honey in Dandi and Meta Robi districts of West Shewa zone, Ethiopia

Style Definition: Heading 1
Formatted: Font: Not Italic
Formatted: Font: Not Italic

Desalegn Begna[1*], Girma Motuma[2¶], Shambel Boki[3¶], Niguse Bekele[4&], Tamiru kuru[5&], Achalu Chimdi[6&]

[1]Agriculture and Rural Development Center, Policy Institute Study Institute, Addis Ababa, Ethiopia

[2] Department of Animal Science, College of Agriculture and Veterinary Science Ambo University Mamo Mezemir Campus, Guder, Ethiopia

[3]Department of Animal Science in the College of Agriculture and Veterinary science Ambo University, Mamo Mezemir Campus, Guder, Ethiopia

[4]Department of Natural Resource Management, Ambo University, Mamo Mezemir Campus, Guder, Ethiopia

[5]Department of Natural Resource Management, Ambo University, Mamo Mezemir Campus, Guder, Ethiopia

[6]Department of Soil Sciences, Ambo University Mamo Mezemir Campus, Guder, Ethiopia

[*]Correspondence author: Desalegn Begna; dbegna67@gmail.com (DB)

[¶] These authors contributed equally to this work.

[&] These authors also contributed equally to this work

Abstract

The study was conducted to characterize the physicochemical properties of honey produced from underground nesting stingless bees (Meliponula beccarii) in the Dandi and Meta Robi districts of the West Shewa zone, Ethiopia. A total of 27 honey samples, including 24 samples collected through careful investigation and excavation of natural nests and 3 samples purchased from the market, were analyzed in the laboratory. The evaluated physicochemical properties showed an overall mean of 306.64±87.95 meq./kg free acidity, 28.05±3.52% moisture content, 1.31±0.44 mS/cm electrical conductivity, 3.29±0.16 pH, 0.89±1.14 mg/kg HMF, 0.63±0.24% mineral (ash), 9.39±4.26% glucose, 0.24±0.01 g/100g sucrose, 10.81±4.95 g/100g maltose, and 16.57±2.55 g/100g fructose, turanose (0.20 ± 0.00 g/100g). The differences between the two district's honey samples were significant (p > 0.05) for fructose value and considerable for free acidity, moisture content, and pH values. The honey samples purchased from the market showed similar physicochemical properties to the honey from the feral nests, with a mean of 314.33±88.72 meq./kg free acidity, 27.73±2.52% moisture content, 1.43±0.41 mS/cm electrical conductivity, 3.26±0.13 pH, 0.95±1.23 mg/kg HMF, 0.59±0.19% mineral (ash), 10.11±4.11% glucose, 0.25±0.02 g/100g sucrose, 11.23±4.52 g/100g maltose, and 16.33±2.41 g/100g fructose. The study found that the stingless bee honey from the study areas had distinctive low HMF, high free acidity, and low pH values, which may indicate the honey's potential medicinal properties. The high free acidity ~~and low pH~~ in the Meliponula beccarii honey appear unusually elevated compared to other stingless bee honey and the Codex Alimentarius standards for Apis honey, suggesting increased fermentation that can originate from the bee species, plant and geographical origins, improper handling, and high moisture content. This study demonstrated that the honey in the study areas has distinctive physicochemical properties from A. mellifera-produced honey, which may support its traditional medicinal uses. Further detailed studies on ground-nesting and other stingless species' honey medicinal values are recommended to provide scientific evidence...

Formatted: Font: Italic

Keywords: *Stingless bee, Physicochemical, honey, properties, moisture*

Introduction

Honey is a sweet, complex substance produced by bees through a series of processes before being stored in honeycombs or pots until it ripens (1). It is primarily composed of sugars, mainly monosaccharides (2–4). Stingless bees, unlike their honeybee (*Apis Mellifera*) counterparts, produce honey from the nectar of flowering plants and store it in pot-shaped structures made of wax cerumen (5).

Studies have shown that stingless bee honey and *Apis* honey possess distinct physicochemical properties. Stingless bee honey generally exhibits greater moisture content, higher acidity, lower sugar composition, and lower enzyme activity compared to Apis honey (6,7). The chemical composition of stingless honey can also vary based on factors such as floral origin (6,8,9), geographical origin, seasonal and environmental conditions, and handling techniques (10–13).

Ethiopia is home to diverse stingless bee species, including the ground-nesting *Meliponula beccarii*, whose honey, locally known as "*damma daamu*," is commonly harvested and used as traditional medicine (3,14,15). *Meliponula beccarii*, a species of stingless bee, is commonly found in Ethiopia and is well known for its honey production as well as its contributions to local biodiversity and pollination. It is particularly prevalent in the Dandi and Meta Robi districts of the West Shewa zone, where it plays a vital role in the ecosystem (16–18). However, the destructive harvesting(3) methods employed may endanger the native stingless bee species and compromise the honey quality (3). Characterization of the physicochemical properties of stingless bee honey in some districts of West Shewa like Cheliya, Jeldu, Wolmera, and Tokekutaye has been studied (3). The Dandi and Meta Robi districts of Ethiopia are known for their stingless bee honey production and traditional medicinal uses. Despite this, the quality and authenticity of the stingless bee honey reported from other districts of west Shewa, information is lacking for Dandi and Meta

Robi districts. Therefore, the primary purpose of this study is to evaluate the physicochemical properties of honey from the native stingless bee (*Meliponula beccarii*) in these two districts.

Material and methods

The study areas

Dandi and Meta Robi are districts located in the West Shewa Zone of the Oromia Regional State in Ethiopia. These two districts were purposefully selected from the West Shewa Zone due to their high potential for stingless bee honey production and ease of access during data collection. According to the 2021/22 Annual Reports from the Agricultural Offices, Dandi district is located at 8°54'0.11"N latitude and 38°25'0.35"E longitude, and experiences minimum and maximum temperatures ranging from 9.3°C to 23.8°C, with annual rainfall between 750-1,300 mm. Meta Robi district is located at 9°19'0.6"N latitude and 38°09'0.6"E longitude and experiences minimum and maximum temperatures ranging from 20°C to 30°C, with annual rainfall between 750-1,300 mm). The locations were purposively chosen for the study representing the high potential areas of stingless bee, *Meliponula beccarii*, and with diverse floral composition.

Sample acquisitions

Careful investigation and excavations of 24 wild stingless bee nests (*Meliponula beccarii*) (8 from Dandi district and 16 from the Meta Robi district) were conducted. From each nest, fresh honey samples ranging from 0.25 to 0.5 liters were collected using disposable 50 ml syringes. In addition, three honey samples were purchased from the market to be used for comparison, making the total honey samples 27. The collected honey samples were then stored in separate, airtight, and labeled jars (Fig 1) and properly refrigerated at 4°C until the dates of the analysis.

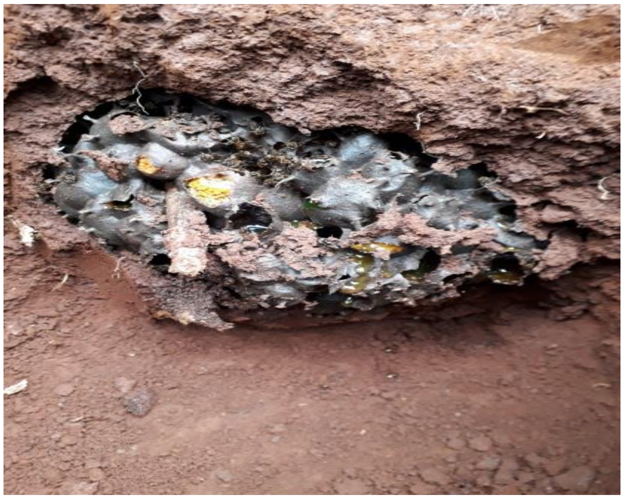

Fig **1**: Excavated feral stingless bee nest

Methodologies and protocols of the honey analysis

Formatted: Font: 18 pt

The analysis of the collected honey samples was done at Ethiopian Conformity Assessment Enterprise (ECAE) in Addis Ababa in accordance of the codex Alimentarius commission of the joint FAO/WHO food standards program (19). The analyzed parameters included sugar content, moisture content, free acidity, pH, electrical conductivity, ash, and HMF each with three replications. Determination of sugars was performed with high-performance liquid chromatography (HPLC) equipped with a differential refractive index (DRI) detector (20) with the separation using NH_2 column (4.6 x 250mm, ZORBAX NH_2) and a particle size diameter of 5µm with the column was kept at 30°C throughout the analysis. The mobile phase composition was 70% acetonitrile in water. The injection volumes of the samples were10µl, with a flow rate of 1.3ml/min. Retention times were identified using standards of peak. The honey samples were also spiked with standards in order to verify the identity of the chromatographic peaks. Triplicate

injections were performed and for the peak quantification average peak areas were used. Monosaccharaides (glucose, fructose) and disaccharides (sucrose, maltose, turanose) were used as standards to determine the sugar content of the honey samples. A 5g of honey sample was dissolved with 25% methanol in water solution. The solution was transferred to a 100ml volumetric flask. Solution was filtered through 0.45µm syringe and injected to HPLC. A standard curve was used to determine sugar content (fructose, glucose, sucrose, turanose and maltose) and the values were calculated as gram/100gram of honey sample.

A digital refractometer (Abbe refractometer, Leica Mark II Plus) thermo stated at 20 ^{O}C with the refractive index and regularly calibrated with distilled water was used (21) to determine the moisture content.

The honey samples were homogenized and the surface of the prism was covered evenly with the sample. After 2 minutes, the refractive index was read with refractometer. Each honey samples were measured triplicate and the average value was taken (21). Free acidity and pH measurement according to (21) Bogdanov (2009). A pH meter was used to measure the pH of a 10% (w/v) solution of honey prepared in deionized water. A 10g honey sample was dissolved in 75 ml of carbon dioxide-free water. It was stirred with the magnetic stirrer, then pH electrode immersed in the solution and the volume of titrate was recorded when titrated with 0.1M NaOH to pH 8.30. The reading was recorded and the free acidity was calculated using the following formula.

$$Free\ acidity(\text{milliequvalent}) = mL\ 0.1M\ NaOH * 10$$

Electrical conductivity was measured using conductivity meter (HANNA, HI 2550, EC/TDS/NaCl meter). 20% (w/v) honey solution was suspended in deionized water. Potassium Chloride solution (0.1M) was used for the cell constant. The electrical conductance of this solution was read in mS after the temperature has been equilibrated to 20˚C (International Honey Commission, 2009). 20g

anhydrous honey was dissolved in distilled water and quantitatively transferred to a 100ml volumetric flask. The electrical conductivity of the honey solution was calculated using the following formula:

$$SH = K * G$$

Where:

SH = electrical conductivity of the honey solution in mS.cm^{-}1

K = cell constant in cm^{-}1

G = conductance in mS

For temperatures above 20 °C: subtract 3.2 % of the value per °C

For temperatures below 20 °C: add 3.2 % of the value per °C

Ash content was determined according to the methods of International Honey Commission (2009). Accordingly, 5 g of honey was placed in combustion pots, which required preheating to darkness. Then, the samples incinerated at high temperature (550℃) in a burning muffle (THERMO CONCEPT, KLS 45/13, Germany) for 1 h. After cooling at room temperature, the obtained ash weighed and the proportion of ash (Ac) in g/100g honey was calculated using the following formula: -

$$AC = \frac{(m1 - m2)}{m3} * 100$$

Where:

m_3 = weight of honey

m_1 = weight of dish + ash,

m_2 = weight of dish

Hydroxymethylfurfural was identified as Standard solution: 5-(hydroxymethyl-) furan-2-carbaldehyde (HMF) 1, 2, 5, 7 and 10 mg /L aqueous solution was prepared. The absorbance A of

the prepared standard solution was determined using an UV spectrophotometer (CECIL, CE 7500, 7000 series) at 285 nm in 1 cm quartz cells with water in the blank cell. The concentration of the standard solutions was calculated from the literature values for molar absorptivity, ε=16830 or absorptivity.

$$\text{Concentration } \left(\frac{\text{mg}}{\text{L}}\right) = \frac{A}{1 * 133.57} * 1000$$

Where: A is the absorbance of the standard solution

HMF content was determined with HMIHC (International Honey Commission, 2009) with some modifications. A high-performance liquid chromatography was used with an absorbance scale 285nm using DAD detector. 10g of prepared honey sample was dissolved with 50ml deionized water. The solution was filtered through a 0.45µm syringe filter and read on a HPLC (Agilent, 1260infinity) using a C18 column (3.0 x 250mm) (ZORBAX SB-C18) kept at 30°C throughout the analysis with a particle size diameter of 5µm. The mobile phase composition was 90% deionized water (1% acetic acid) with methanol. The injection volumes of the samples were 10µl, with a flow rate of 0.7ml/min (International Honey Commission, 2009). The HMF content of the samples was calculated by comparing the corresponding peak areas of the samples and those of the standard solutions taking into account the dilution. The collected data were systematically organized, coded, tabulated, and analyzed using the Statistical Package for Social Science (SPSS) software version 20. One-way ANOVA was employed to compute the variations between the means of each variable in every studied parameter and 0.05% tests were considered significance.

Results and discussion

Free Aacidity

In this study, the overall mean free acidity values of the stingless bee honey samples were 306.64 ± 87.95 meq/kg, ranging from 200-424.74 meq/kg (mean 338.79 ± 72.46) for the Dandi district, and 188.25-456.30 meq/kg (mean 274.49 ± 93.10) for the Meta Robi district. The mean free acid value for the purchased honey sample was 348.5 ± 12.0 meq/kg, and there was no significant difference (P>0.05) between the freshly collected honey samples from the two districts and the purchased sample (Table 1).

The mean free acidity values in this study were well above the acceptable limit of international standards for *Apis mellifera* honey (<50 meq/kg) (19)-, further confirming the acidic nature of the stingless bee honey (6). -Reports from Ethiopia indicate mean free acidity levels of 92.39 ± 4.45 meq/kg (18) and 7.3 ± 0.36 meq/kg (3) for stingless bee honey samples from various parts of the country, both of which are significantly lower than our findings. Our value is also notably higher than the free acidity of stingless bee honey from Southeast Asia (40 to 60 meq/kg) and South America (50 to 70 meq/kg). The elevated free acidity of *Meliponula beccarii* honey in our samples is likely attributed to a combination of diverse floral sources with high organic acid content (22), specific environmental conditions that promote fermentation (23), and unique soil characteristics influencing nectar composition (24), Honey acidity is influenced by enzymatic activity and low pH content (25).

The total acidity of honey can vary depending on the nectar pH, mineral (ash) content derived from the soil composition, or the plant species from which the final honey composition is derived (25).

Honey free acidity is an important quality parameter, as it indicates the presence of organic acids,

which contribute to the flavor and aroma of honey (11). Higher acidity values suggest a greater abundance of organic acids (26). Honey acidity is influenced by enzymatic activity and low pH content (26). Acidity values can also be useful for discriminating honeys of different floral origins, and honey adulterated with sugar syrup typically has very low free acidity (<1) (26). The acidity levels in stingless bee honeys can vary significantly based on factors like bee species, geographical origin, and other variables. The free acidity values were reported 26.5 to 66.4 meq/kg for *Melipona scutellaris* honey (Souza et al., 2006), 29.7 ± 4.4 meq/kg for *Plebeia saiqui* honey, and 42.7 ± 4.2 meq/kg for *Tetragonisca angustula* honey (Vit et al., 1998), and 22.4 to 34.6 mEq/kg for *Melipona beecheii* honey (Ruiz-Argueso and Rodriguez-Navarro, 1973). The *Meliponula beccarii* honey studied appears to have unusually high free acidity and low pH compared to stingless bee honey from Brazil (Melipona scutellaris), Venezuela (Plebeia saiqui, Tetragonisca angustula), and Mexico (Melipona beecheii), which typically range from 22.4 to 66.4 meq/kg. This suggests the high free acidity may be due to increased fermentation, potentially stemming from factors like differences in bee species and geographical origin (27), improper handling, or high moisture content (28), which requires further investigation. The higher free acidity in honey is directly linked to its enhanced medicinal potential, and honey with higher free acidity (lower pH) exhibits stronger antimicrobial activity against pathogens (Kadariya et al., 2020; Olaitan et al., 2022; Sherlock et al., 2010), greater antioxidant capacity (7,10), improved anti-inflammatory and wound healing properties (29), and effective antibiofilm activity that can disrupt and prevent microbial biofilms (30,31), collectively highlighting the direct relationship between the high free acidity in honey and its potential for medicinal and nutritional value. of honey.

Moisture content

Honey moisture content is an important quality parameter due to its impact on fermentation. The overall moisture content of the honey samples was reported to be between 23.45% and 36.75%.(mean 28.05±3.52%). The recorded moisture contents ranged from 25-36.75% (mean 29.34±4.02%) for the Dandi district and 23.45-30.80% (mean 26.77±2.49%) for the Meta Robi district. The purchased honey samples had a moisture content of 34.20-36.90% (mean 35.40±0.79%), and there was no statistical difference ($P > 0.05$) between the honey samples from the two districts (Table 1). The moisture content of freshly collected honey was lower than the in marketed honey and this lower moisture content helps prevent fermentation, indicating better quality preservation of the freshly collected honey sample (23).

This finding is consistent with previous studies. (3)reported a moisture content of 25.1 - 35.0% (mean 29.6±1.4%) for honey samples of the same stingless bee species collected from four districts of Ethiopia. (32) also reported similar results. Studies have shown that stingless bee honey from Southeast Asia (Malaysia) typically has a moisture content ranging from 30% to 35% (24) and in contrast, honey from South America has been observed to have slightly lower moisture content, between 28% and 30% and Brazilian stingless bee honey exhibited moisture levels around 28% (22).

Variations in moisture content among honey samples can be affected by factors such as adulteration, humidity, floral origin, soil type, collection period, and processing aspects (12,33–35). The moisture contents noted in this study are lower than what was reported for stingless bee honeys from Mexico, Guatemala, and Venezuela (31.4% to 38.74%) (36), but higher than the 13.86±1.06% reported for Melipona species in Nigeria (37).

The standard moisture content limit for *Apis mellifera* honey is 20% or less, as per the Ethiopian (26) and European (21)standards.

Table 1: Mean physicochemical properties of stingless bee honey samples (N = 27)

Values	Parameters										
	Free Acidity meq/kg	MC %	EC mS/cm	HMF Mg/kg	pH	Ash (g)	Glu (g)	suc (g)	Malt (g)	Fruc (g)	Tura
**Dandi district (N=8)**											
Min.	200.00	25.00	0.72	0.07	3.00	0.40	1.20	0.20	6.50	12.00	0.1
Max.	424.74	36.75	1.99	4.20	3.42	1.00	15.80	0.25	16.80	16.80	0.2
Mean± std	338.79±72.46	29.34±4.04	1.38±0.48	1.14±1.53	3.24±0.13	0.65±0.25	8.84±5.29	0.24±0.01	10.27±3.98	14.50±1.54	0.20
**Meta Robi district(N=16)**											
Min.	188.25	23.45	0.75	0.06	3.00	0.36	5.80	0.22	5.20	15.90	0.1
Max.	456.30	30.80	1.85	1.30	3.60	0.95	13.40	0.26	20.00	20.00	0.2
Mean± std	274.49± 93.1	26.77±2.49	1.24±0.4	0.64±0.46	3.35±0.18	0.61±0.23	9.94±3.05	0.24±0.01	11.35±5.91	18.63±1.38	0.20±
**Total (Dendi and meta Robi)**											
Min.	188.25	23.45	0.72	0.06	3.00	0.36	1.20	0.20	5.20	12.00	0.1
Max.	456.30	36.75	1.99	4.20	3.60	1.00	15.80	0.26	20.00	20.00	0.2
Mean ± std	306.64±87.95	28.05±3.52	1.31±0.44	0.89±1.14	3.29±0.16	0.63±0.24	9.39±4.26	0.24±0.01	10.81±4.95	16.57±2.55	0.20±
P-Value	0.07	0.07	0.44	0.29	0.08	0.71	0.54	0.89	0.61	0.00	0.5
**Purchased honey (N=3)**											
Mean± std	348.5±12.08	35.40±0.79	1.50±0.14	0.28±0.2	3.31±0.02	0.64±0.09	9.53±0.52	0.25±0.0	7.8±2.0	13±0.23	0.20±
Max.	372.66	36.90	1.79	0.70	3.35	0.83	10.2	0.25	12	13.4	0.2
Min.	336.5	34.20	1.34	0.08	3.27	0.54	8.50	0.25	5.5	12.6	0.2
P-Value	0.078	0.001	0.005	0.01	0.30	0.005	0.20	0.03	0.01	0.01	
Codex Alimentarius Standard for Honey (CODEX STAN 12-1981, Rev.2-2001)	50	20	0.8	40	3.4 - 6.1	<0.6	N/A	5	N/A	N/A	N/
Southeast Asia	40-60	30-35	0.5-1.0	5-10	3.5-4.5	0.1-0.3	25-35	1-2	1-2	35-45	
South America	50-70	28-30	0.3-0.8	10-20	3.0-4.0	0.2-0.5	25-30	1-3	1-2	40-50	

Formatted Table

N=Number of samples, MC=Moisture content, HMF=hydroxyl methyl furfural, EC=Electric conductivity, pH=pH value, Std.D=Standard deviation, Min=minimum, Max=Maximum, MC

(Moisture content), HMF (hydroxymethylfurfural,) EC (Electrical Conductivity), Tura (Turanose,) Glu (Glucose), Suc (sucrose), Fruc (Fructose), Malt (Maltose)

,

The pH value

The mean pH values ranged from 3.0-3.42 (mean 3.24±0.13) for the Dandi district and 3.0-3.60 (mean 3.35±0.18) for the Meta Robi district. The purchased honey samples had a pH range of 3.27-3.35 (mean 3.31±0.023). The pH parameter showed no statistically significant differences ($p>0.05$) among all the sampled honeys (Table 1).

All honey samples showed low pH values, which is characteristic of stingless bee honeys (5,6). This finding is consistent with previous studies(3) reported an overall pH range of 3.4-3.9 (mean 3.7±0.15) for stingless bee honey from four districts in Ethiopia. (37) reported pH values of 3.75 and 4.21 for stingless bee honey (Melipona species) in Nigeria, and (40) presented similar findings for the genus *Melipona* in Brazil.(22). The pH of honey from Southeast Asia typically varies from 3.5 to 4.5, as reported by (24) and honey from South America exhibits a pH range of 3.0 to 4.0 according to findings by (22), indicating the *Meliponula beccarii* honey from Ethiopia has a pH, which falls within the lower spectrum of both Southeast Asian and South American honeys. Lower pH may enhance the honey's preservation and antibacterial properties, contributing to its quality (23).

Formatted: Font: Italic

Formatted: Font: Italic

The standard pH value for *Apis mellifera* honey is 3.42–6.10 (41,42). The low pH value of Melipona honey has great significance for its extraction and storage, as it inhibits the occurrence and development of microorganisms (43,44) and reduces some infectious diseases (45,46);.

Ash contents

In this study, the ash contents ranged from 0.40-1.0 (mean 0.65 ± 0.25g) for the Dandi district, 0.36-1g (mean 0.63±0.24g) for the Meta Robi district, and 0.54-0.83g (mean 0.64± 0.09g) for the purchased honey samples. There was no significant difference ($P < 0.05$) in the ash content among all the samples (Table 1).

The mineral composition of honey could be influenced by the geographical and botanical origin of the flora, the type and activity of the bees, the extraction technique, and the storage conditions (47). The results of this study are consistent with the findings of (3), who reported ash contents ranging from 0.21 to 0.57% for stingless bee honey. Although the overall average ash content of stingless bee honey is similar to the purchased honey from the market (0.5 ± 0.00%), the results of the present study suggest that honey produced from the ~~native~~ stingless bee (*M. beccarii*) in Ethiopia is richer in mineral content.

This is significant, as the mineral content in honey can have important implications for its for various aspects; used as a marker to identify the geographical origin of the honey, allow for honey traceability and authentication (48); and indicates the predominant floral sources that the bees have visited, as different plant species accumulate varying levels of minerals in their nectar (49); used to assess the quality and authenticity of honey, as adulteration or improper processing can alter the natural mineral profile (50); and it is relevant to the honey's nutritional value, as it provides information about the bioavailability of essential minerals, such as potassium, calcium, and magnesium (51). It is also established that the ash content of stingless bee honey varies by region, with Southeast Asia ranging from 0.1% to 0.3% (24) and South America between 0.2% and 0.5% (22). The lower ash eontents for *Meliponula beccarii* honey from Ethiopia suggests that Ethiopian honey may have a reduced mineral profile, likely due to the specific flora and soil characteristics

Formatted: Justified, Space After: 0 pt, Line spacing: Double

Formatted: Font: Italic

in the region and such differences highlight the impact of local biodiversity on honey composition and its potential nutritional value (22–24).

Sugar contents

The mean sugar concentrations reported were glucose (9.39 ± 4.26 g/100g), sucrose (0.24 ± 0.01 g/100g), maltose (10.81 ± 4.95 g/100g), fructose (16.57 ± 2.55 g/100g), and turanose (0.20 ± 0.00 g/100g). Interestingly, the only significant difference ($p<0.05$) between the two districts was observed for the fructose concentration. Comparing these results to a previous study, the sugar concentrations recorded were generally lower are fructose (36.48 ± 0.54 g/100g), glucose (27.67 ± 0.43 g/100g), and sucrose (1.24 ± 0.18 g/100g) in stingless bee honey samples (32).

Formatted: Indent: First line: 0.5"

The Melipona honeys, typically contain small quantities of maltose (52). The sugar composition reveals that Southeast Asian honey has fructose levels ranging from 35% to 45% and glucose levels from 25% to 35%. South American honey also shows high fructose levels of 40% to 50%, which aligns closely with the Southeast Asian findings. The balance of these sugars influences the taste, sweetness, and overall quality of the honey (22,23).

Previous studies has reported maltose levels in stingless bee honey generally range from lower values, typically around 1% to 5% (22,23). However, the high maltose content in our stingless bee honey may be due to several factors like specific floral sources can significantly influence sugar composition, with certain plants containing higher maltose concentrations (22), fermentation processes within the nest may also increase maltose levels if honey is stored for extended periods (23), the foraging behavior of stingless bees that could lead to the preferential collection of nectar from maltose-rich plants and environmental factors, such as soil composition and climate, that may further impact floral diversity and nectar quality, resulting in elevated maltose levels (53),

processing and storage methods might alter sugar profiles, with prolonged storage or heat affecting composition (24), variability among stingless bee species (23).

These differences in sugar composition could be attributed to various factors, such as the geographical and botanical origins of the honey, the species of stingless bees involved, and the extraction and processing methods employed (27,48,50,51,54).

Hydroxymethylfurfural (HMF)

In this study, 0.06 and 4.2 values were recorded as minimum and maximum with an overall mean of 0.89±1.14 mg/kg (

). The highest HMF value was recorded in honey samples collected from the Dandi district (4.20 mg/kg), and the difference in HMF value recorded in honey samples collected from both districts is not significant (p>0.05). This finding disagrees with (3) that stated a high overall HMF range (11.2–22.4mg/kg) and the value reported by (32) (6.58±0.36mg/kg) for pooled honey samples from 14 districts of Oromia, Ethiopia. (19) permits a maximum of 40 mg/kg for the HMF standard. The low HMF content including the purchased honey sample in this study indicates the freshness and good quality of the honey (55–57). The Hydroxymethylfurfural (HMF) is used as an indicator of poor quality due to adulteration or poor handling like inappropriate storage and the lower the HMF, the freshness and good quality of the honey (9). HMF levels in Southeast Asian honey are reported at 5 to 10 mg/kg, while in South America, they can range from 10 to 20 mg/kg, indicating Ethiopian honey has an HMF content within the acceptable limits for freshness (23,53).

Electrical conductivity

The overall mean calculated value of electrical conductivity of the 24 honey samples of stingless bees from the two study districts was 1.31±0.44 mS/cm, ranging from 0.72-1.99. The electrical conductivity values ranged from 0.72-1.99 mS/cm (mean 1.38±0.48) for the Dandi

district samples, 0.75-1.85mS/cm (mean 1.24±0.40) for the Meta Robi district and 0.14-1.50mS/cm (mean 1.79±1.34) for purchased honey (Table 1). The electrical conductivity difference of the two-district sampled honey is nto significant (P>0.05), suggesting similarities of flora compositions between the districts. The electrical conductivity in the current study is in line with (32) and (3) on stingless bee honey in Ethiopia. The conductivity values of the Melipona honeys vary from 0.32-0.44mS/cm (58). The electrical conductivity values of the explored honey samples were within the range of allowable value of international standard value (i.e, greater than 0.8 mS/cm) (19). The significance differences in electric conductivity of honey samples between the two locations suggest its usefulness for differentiating honey of different botanical origins and purity (26).

The unique findings from the data are that the stingless bee (*Meliponula beccarii*) honey from the Dandi and Meta Robi districts of Ethiopia exhibits high acidity, low pH, low HMF, and a distinctive sugar profile, differentiating it from other places studied stingless bee honey (3) indicating potential medicinal properties.

Conclusion

This study evaluated This study evaluated the physicochemical properties of honey produced by the ground-nesting stingless bee (*Meliponula beccarii*) in Ethiopia and compared it to the Codex Alimentarius standards for *Apis mellifera* honey, revealing several notable differences:

This study assessed the physicochemical properties of honey produced by the ground-nesting stingless bee (Meliponula beccarii) in Ethiopia and compared these properties to those of stingless bee honey of same species from Ethiopia, stingless bee honey from Southeast Asia, and South America, as well as to the Codex Alimentarius standards for *Apis mellifera* honey and showed both similarities and differences. the stingless bee honey exhibited considerably higher free acidity (306.64 meq/kg vs. 50 meq/kg), higher moisture content (28.05% vs. 20%), lower pH (3.29 vs. 3.4-6.1), and higher electrical conductivity (1.31 mS/cm vs. 0.8 mS/cm), while having a hydroxymethylfurfural (HMF) content (0.89 mg/kg) well within the Codex standard (40 mg/kg); the unique low pH and high acidity of the stingless bee honey contribute to its potential medicinal value, including robust antimicrobial activity, impressive antioxidant capacity, and enhanced anti-inflammatory and wound healing properties, and the low HMF content indicates its freshness and good quality, underscoring the exceptional physicochemical profile of this honey and positioning it as a highly promising natural remedy with significant medicinal potential, with important implications for its standardization and utilization, particularly for medicinal and nutritional applications.However, the study's shortcomings include its limited scope, lack of seasonal variations, absence of bioactive compound profiling, and lack of comparative analysis

Furthermore, the study highlights the distinctive physicochemical profile of stingless bee honey, characterized by significantly higher free acidity (306.64 meq/kg vs. 50 meq/kg), elevated moisture content (28.05% vs. 20%), lower pH (3.29 vs. 3.4-6.1), and higher electrical conductivity (1.31 mS/cm vs. 0.8 mS/cm). Its hydroxymethylfurfural (HMF) content (0.89 mg/kg) is well within Codex standards (40 mg/kg), indicating freshness and quality. The unique low pH and high acidity contribute to potential medicinal benefits. A key finding of this study is the variability between freshly collected and marketed honey samples, with fresh honey consistently demonstrating superior quality indicators compared to marketed counterparts, which often have higher acidity and moisture levels underscoring the need for specific quality standards and regulations for stingless bee honey to ensure its authenticity and efficacy.

Formatted: Indent: First line: 0"

While the promising characteristics of stingless bee honey position it as a valuable natural remedy, the study also acknowledges limitations, including a restricted scope, lack of seasonal variation analysis, absence of bioactive compound profiling, and insufficient comparative studies with other honey types. Addressing these gaps in future research will enhance our understanding of stingless bee honey's full potential for medicinal and nutritional applications, supporting its standardization and utilization across various sectors.

Recommendations

Based on the findings of this study, establishing specific quality standards and regulations for stingless bee (Meliponula beccarii) honey, given its distinctive physicochemical profile that differs from the Codex Alimentarius standards *for Apis mellifera honey*, and develop certification and labeling schemes to ensure its authenticity and quality; explore the potential medicinal applications of stingless bee honey, leveraging its unique properties such as high acidity, low pH,

and low HMF content, and conduct further research to fully characterize its bioactive compounds and therapeutic potential; investigate the potential nutritional benefits. While promoting the diversification of honey sources; support the conservation and sustainable management of stingless bee populations, and develop training and extension programs to educate local communities on their importance; and encourage interdisciplinary collaboration between researchers, beneficiaries, policymakers, and end-users to foster the development and utilization of stingless bee honey as a valuable natural resource.

Acknowledgment

The authors wish to express their gratitude to Ambo University, Mamo Mezemir Campus, Department of Animal Science, for allowing this work to proceed and for its unwavering support throughout the data collection process.

References

1. FAO. The state of food and agriculture. Soil Sci [Internet]. 2009;95(4):11. Available from: https://www.fao.org/3/i0100e/i0100e.pdf
2. Cantarelli MA, Pellerano R, Marchevsky E, Camiña J. Quality of honey from Argentina: study of chemical composition and trace elements. J Argentine Chem Soc. 2008 Dec;96:33–41.
3. Gela A, Hora ZA, Kebebe D, Gebresilassie A. Physico-chemical characteristics of honey produced by stingless bees (Meliponula beccarii) from West Showa zone of Oromia Region, Ethiopia. Heliyon. 2021;7(1).
4. Shugaba A. Analysis of biochemical composition of honey samples from North-East Nigeria. Biochem Anal Biochem. 2012;2(3).
5. Kwapong P, Aidoo K, Combey R, Karikari A. Stingless bees: importance, management and utilization. A training manual for stingless beekeeping. Accra North: Uni Max Macmillan; 2010.
6. Nordin A, Sainik NQAV, Chowdhury SR, Saim A Bin, Idrus RBH. Physicochemical properties of stingless bee honey from around the globe: a comprehensive review. J Food Compos Anal [Internet]. 2018;73:91–102. Available from: https://www.sciencedirect.com/science/article/pii/S0889157518302953
7. Sadia H, Anjum AA, Yasmeen R, Javed F, Zafar MA, Khan IU, Inam-ul-Haq M. Comparative analysis of antioxidant potential and antimicrobial activity of different honey samples. Vet World. 2022;15(4):1034–41.
8. Kahraman T, Buyukunal SK, Vural A, Altunatmaz SS. Physico-chemical properties in honey from different regions of Turkey. Food Chem. 2010;123:41–44.

9. L.C. Marchini, A.C.d.C.C. Moreti, I.P. Otsuk G d. SS. Physicochemical composition of Apis mellifera honey samples from São Paulo state, Brazil. 30(7):1653–7.

10. Alvarez-Suarez JM, Tulipani S, Díaz D, Estevez Y, Romandini S, Giampieri F, et al. Antioxidant and antimicrobial capacity of several monofloral Cuban honeys and their correlation with color, polyphenol content and other chemical compounds. Food Chem Toxicol [Internet]. 2010;48(8):2490–9. Available from: https://www.sciencedirect.com/science/article/pii/S0278691510003959

11. Maia-Silva C, Hrncir M, Imperatriz-Fonseca VL, Schorkopf DLP. Stingless bees (Melipona subnitida) adjust brood production rather than foraging activity in response to changes in pollen stores. J Comp Physiol A Neuroethol Sensory, Neural, Behav Physiol. 2016;202(9–10):723–32.

12. Norowi M, Sajap H, Rosliza ASJ, Mohd Fahimie AJ, Suri R. Conservation and sustainable utilization of stingless bees for pollination services in agricultural ecosystems in Malaysia. In: Proceeding of International Seminar of Enhancement of Functional Biodiversity Relevant to Sustainable Production in ASPAC – In Association With MARCO. 2010.

13. Vit P, Roubik DW, Pedro SRM. Pot-honey: a legacy of stingless bees. Pot-Honey: A Legacy of Stingless Bees. 2012. 1–654 p.

14. Mogessie A. The in vitro antibacterial activity of ‘Tazma mar’ honey produced by stingless bee. Ethiop J Heal Dev. 1994;8(2):109–17.

15. Wondmeneh Jemberie, Worku Negash, Kassahun Alemu, Alemu Tarekegn MB, NR. Stingless bee Meliponula Cockerell (Hymenoptera: Apidae: Meliponini) ground nest architecture and traditional knowledge on the use of honey in the Amhara Region, Northwest Ethiopia. Isr J Entomol. 2020;50(March 2021):14–7.

16. Getachew T, Tesfaye G, Tadesse A. The ecology of stingless bees in Ethiopia. J Apic Res. 2021;60(4):345–58.

17. Tesfaye G, Tadesse A. Distribution and conservation of Meliponula beccarii in Ethiopia. Ethiop J Biol Sci. 2022;18(2):123–34.

18. Damto T, Kebebe D, Gemeda M. Physicochemical properties and microbial qualities of honey produced by a stingless bee (Meliponula beccarii L.) in the Oromia region, Ethiopia. Cogent Food Agric [Internet]. 2023;9(1). Available from: https://doi.org/10.1080/23311932.2023.2258784

19. FAO/WHO Codex Alimentarius. Procedural manual, twenty-seventh edition. 2019. 254 p.

20. AOAC. Analytical Methods. Toxicol Ind Health. 2000;15(8):715–7.

21. Bogdanov S. Harmonised Methods of the International IHC. Bee Prod Sci. 2009;(5):1–62.

22. Cavalcante IG, Santos LS, Freitas BM. Physicochemical properties of stingless bee honey from Brazil. J Apic Res. 2020;59(2):182–90.

23. Silveira FA, Oliveira JA, Ferreira JR. Comparative analysis of stingless bee honey from Venezuela and Brazil. Ethiop J Biol Sci. 2021;18(1):67–75.

24. Tey SL, Tan KW, Lee CY. Quality assessment of stingless bee honey from Southeast Asia. Food Chem. 2022;370:131045.

25. Stramm KM. Composition and quality of honey bee Jandaira (Melipona subnitida), storage effects, and comparison with Apis mellifera honey. 2011.

26. Živkov Baloš M, Popov N, Vidaković S, Ljubojević Pelić D, Pelić M, Mihaljev Ž, et al. Electrical conductivity and acidity of honey. Arch Vet Med. 2018;11(1):91–101.

27. Souza R, Roubik D, Barth E. Composition of stingless bee honey: setting quality standards. Interciencia. 2006;31(12).

28. Al-Tikrity WS, Benton AW, Hillmann RC, Clarke WW. The relationship between the amount of HMF in honey and its fermentation. Am Bee J. 1989;129(7):478–81.
29. Almasaudi SB, El-Shitany NA, Abbas AT, Abdel-Dayem UA, Ali SS, Al Jaouni SK, Harakeh S. Antioxidant, anti-inflammatory, and antiulcer potential of manuka honey against gastric ulcer in rats. Oxid Med Cell Longev. 2017.
30. Majtan J, Bohova J, Horniackova M, Klaudiny J, Majtan V. Anti-biofilm effects of honey against wound pathogens Proteus mirabilis and Enterobacter cloacae. Phyther Res. 2014;28(1):69–75.
31. Kilty SJ, Duval M, Chan FT, Ferris W, Slinger R. Methylglyoxal: active agent of manuka honey in vitro activity against bacterial biofilms. Int Forum Allergy Rhinol. 2011;1(5):348–50.
32. Teferi Damto DK, Meseret. Physico-chemical and microbiological characteristics of honey produced by stingless bees. 2022.
33. Vit P, Oddo L, Marano M, Mejias E. Venezuelan stingless bee honey characterized by multivariate analysis of physicochemical properties. Apidologie. 1998;(29):377–89.
34. Biluca FC, Della Betta F, de Oliveira GP, Pereira LM, Gonzaga LV, Costa ACO. 5-HMF and carbohydrates content in stingless bee (Meliponinae spp.) honey by CE before and after thermal treatment. Food Chem. 2014;159:244–9.
35. Biluca FC, Braghini F, Gonzaga LV, Oliveira Costa AC, Fett R. Physicochemical profiles, minerals and bioactive compounds of stingless bee honey (Meliponinae). J Food Compos Anal. 2016;50:61–9.
36. Vit P, Roubik DW, Pedro SRM. Pot-honey: a legacy of stingless bees. Pot-Honey: A Legacy of Stingless Bees. 2012. 1–654 p.

37. Nweze JA, Okafor J, Nweze EI. Evaluation of physicochemical and antioxidant properties of two stingless bee honeys: a comparison with Apis mellifera honey from Nsukka, Nigeria. BMC Res Notes. 2017;10:566.

38. Quality and Standards Authority of Ethiopia (QSAE). ET 1202: Honey - Specification [Internet]. 2005. Available from: https://archive.org/search?query=subject%3A%22public.resource.org%22

39. Quality and Standards Authority of Ethiopia (QSAE). Quality and Standards Authority of Ethiopia (QSAE) Honey Specification: Ethiopian Standard, ES 1202. 2005.

40. Souza ECA, Menezes C, Flach A. Stingless bee honey (Hymenoptera, Apidae, Meliponini): a review of quality control, chemical profile, and biological potential. Apidologie. 2021;52(1):113–32.

41. Bekele Tesfaye, Deaslegn Begna ME. Evaluation of physicochemical properties of honey produced in Bale Natural Forest, Southeastern Ethiopia. Int J Agric Sci Food Technol. 2016;1(2).

42. Bogdanov S, Jurendic T, Sieber R, Gallmann P. Honey for nutrition and health: a review. J Am Coll Nutr. 2009 Jan 1;27:677–89.

43. Silva JMC, Barbosa LCF, Leal IR TM. The Caatinga: understanding the challenges. In: Silva JMC, Leal IR, Tabarelli M (eds) Caatinga: the largest tropical dry forest region in South America. Springer, Cham. 2017;3–19.

44. Alves DA, Imperatriz-Fonseca VL, Francoy TM, Santos-Filho PS, Bil-len JW. Successful maintenance of a stingless bee population despite a severe genetic bottleneck. Conserv Genet. 2011;2:647–58.

45. Mushonga B, Hategekimana L, Habarugira G, Kandiwa E, Samkange A, Segwagwe BV. Characterization of the beekeeping value chain: challenges, perceptions, limitations, and opportunities for beekeepers in Kayonza District, Rwanda. Adv Agric. 2019;2019.
46. Meo SA, Al-Asiri SA, Mahesar AL. Role of honey in modern medicine. Saudi J Biol Sci. 2017;24(5):975–8.
47. Nnadi PC. Determination and comparative evaluation of mineral content of honey obtained from Rivers State University, Forestry Arboretum and Port Harcourt Local Market. J Appl Sci Environ Manag. 2020;24(9):1593–7.
48. Anklam E. A review of the analytical methods to determine the geographical and botanical origin of honey. Food Chem. 1998;63:549–62.
49. Machado De-Melo AA, Almeida-Muradian LB de, Sancho MT, Pascual-Maté A. Composición y propiedades de la miel de Apis mellifera: una revisión. J Apic Res. 2018;57(1):5–37.
50. Downey G, Fouratier V, Kelly JD. A review of the analytical methods to determine the geographical and botanical origin of honey. J Agric Food Chem. 2003;51(2):347–54.
51. Karabagias IK, Karabournioti S, Karabagias VK, Badeka AV. Palynological, physico-chemical and bioactivity parameters determination of a less common Greek honeydew honey: "dryomelo." Food Control [Internet]. 2020;109:106940. Available from: https://www.sciencedirect.com/science/article/pii/S0956713519305298
52. Bogdanov S, D'Arcy BR, Mossel B, Marcazzan GL. Honey quality and international regulatory standards: review by the International Honey International honey standards are specified in a European Honey. Bee World. 1999;80(2):61–9.

53. Tan KW, Lim HP, Lee CY. Physicochemical properties of honey from stingless bees in Malaysia. J Apic Res. 2021;60(1):45–54.

54. Couvillon MJ, Wenseleers T, Imperatriz-Fonseca VL, Nogueira-Neto P, Ratnieks FLW. A comparative study in stingless bees (Meliponini) demonstrates that nest entrance size predicts traffic and defensivity. J Evol Biol. 2008;21(1):194–201.

55. Zulkhairi Amin FA, Sabri S, Mohammad SM, Ismail M, Chan KW, Ismail N, et al.Therapeutic properties of stingless bee honey in comparison with European bee honey. Adv Pharmacol Sci. 2018;2018.

56. Kiprono SJ, Mengich G, Kosgei J, Mutai C, Kimoloi S. Ethnomedicinal uses of stingless bee honey among native communities of Baringo County, Kenya. Sci African [Internet]. 2022;17:e01297. Available from: https://doi.org/10.1016/j.sciaf.2022.e01297

57. Sabella JK, Gladys M, Bartholomew NO, Charles M, Sammy K. Therapeutic uses of stingless bee honey by traditional medicine practitioners in Baringo County, Kenya. J Pharmacogn Phyther. 2022;14(3):27–36.

58. Bogdanov S, Vit P, Kilchenmann V. Sugar profiles and conductivity of stingless bee honeys from Venezuela. Apidologie. 1996;27(6):445–50.

1. ~~FAO. The State of Food and Agriculture. Soil Sci [Internet]. 2009;95(4):11. Available from: https://www.fao.org/3/i0100e/i0100e.pdf~~

2. ~~Cantarelli MA, Pellerano R, Marchevsky E, Camiña J. Quality of honey from Argentina: Study of chemical composition and trace elements. J Argentine Chem Soc. 2008 Dec;96:33–41.~~

3. ~~Gela A, Hora ZA, Kebebe D, Gebresilassie A. Physico-chemical characteristics of honey produced by stingless bees (Meliponula beccarii) from West Showa zone of Oromia~~

Region, Ethiopia. Heliyon. 2021;7(1).

4. Shugaba A. Analysis of Biochemical Composition of Honey Samples from North-East Nigeria. Biochem Anal Biochem. 2012;2(3).

5. Kwapong P, Aidoo K, Combey R, and Karikari A. Stingless Bees: Importance, Management and Utilization. A Training Manual for Stingless Beekeeping. Uni Max Macmillan Ltd. Ghana: Accra North. 72. 2010;

6. Nordin A, Sainik NQAV, Chowdhury SR, Saim A Bin, Idrus RBH. Physicochemical properties of stingless bee honey from around the globe: A comprehensive review. J Food Compos Anal [Internet]. 2018;73:91–102. Available from: https://www.sciencedirect.com/science/article/pii/S0889157518302953

7. Sadia, H., Anjum, A. A., Yasmeen, R., Javed, F., Zafar, M. A., Khan, I. U., & Inam-ul-Haq M. Comparative analysis of antioxidant potential and antimicrobial activity of different honey sample. Vet World,. 2022;15(4):1034–41.

8. Kahraman, T., Buyukunal, S.K., Vural, A. and Altunatmaz SS. Physico-chemical properties in honey from different regions of Turkey. Food Chem. 2010;123: 41-44.

9. L.C. Marchini, A.C.d.C.C. Moreti, I.P. Otsuk G d. SS. Physicochemical composition of Apis mellifera honey samples from São Paulo state, Brazil. 30(7):1653–7.

10. Alvarez-Suarez JM, Tulipani S, Díaz D, Estevez Y, Romandini S, Giampieri F, et al. Antioxidant and antimicrobial capacity of several monofloral Cuban honeys and their correlation with color, polyphenol content and other chemical compounds. Food Chem Toxicol [Internet]. 2010;48(8):2490–9. Available from: https://www.sciencedirect.com/science/article/pii/S0278691510003959

11. Maia-Silva C, Hrncir M, Imperatriz-Fonseca VL, Schorkopf DLP. Stingless bees

(Melipona subnitida) adjust brood production rather than foraging activity in response to changes in pollen stores. J Comp Physiol A Neuroethol Sensory, Neural, Behav Physiol. 2016;202(9–10):723–32.

12. Norowi, M., Sajap, H., Rosliza, A. S. J., Mohd Fahimie, A. J. & Suri R. Conservation and sustainable utilization of stingless bees for pollination services in agricultural ecosystems in Malaysia. In: Proceeding of International Seminar of Enhancement of Functional Biodiversity Relevant to Sustainable Production in ASPAC – In Association With MARCO. 2010.

13. Vit P, Roubik DW, Pedro SRM. Pot-Honey: A legacy of stingless bees. Pot-Honey: A Legacy of Stingless Bees. 2012. 1–654 p.

14. Mogessie A. The in vitro antibacterial activity of 'Tazma mar' honey produced by sting less bee. Ethiop J Heal Dev. 1994;8(2):109–17.

15. Wondmeneh Jemberie, Worku Negash, Kassahun Alemu, Alemu Tarekegn MB& NR. Stingless bee Meliponula Cockerell (Hymenoptera: Apidae: Meliponini) ground nest architecture and traditional knowledge on the use of honey in the Amhara Region, Northwest Ethiopia. Isr J Entomol. 2020;50(March 2021):14–7.

16. Getachew, T., Tesfaye, G., & Tadesse A. The ecology of stingless bees in Ethiopia. J Apic Res. 2021;60(4):345-358.

17. Tesfaye, G., & Tadesse A. Distribution and conservation of Meliponula beccarii in Ethiopia. Ethiop J Biol Sci. 2022;18(2):123-134.

18. Damto T, Kebebe D, Gemeda M. Physicochemical properties and microbial qualities of honey produced by a stingless bee (Meliponula beccarii L.) in the Oromia region, Ethiopia. Cogent Food Agric [Internet]. 2023;9(1). Available from:

https://doi.org/10.1080/23311932.2023.2258784

19. FAO/WHO Codex Alimentarius. Procedural manual, twenty-seventh edition. 2019. 254 p.

20. (AOAC. Analytical Methods. Toxicol Ind Health. 2000;15(8):715–7.

21. Bogdanov S. Harmonised Methods of the International IHC. Bee Prod Sci. 2009;(5):1–62.

22. Cavalcante, I. G., Santos, L. S., & Freitas BM. Physicochemical properties of stingless bee honey from Brazil. J Apic Res. 2020;59(2):182-190.

23. Silveira, F. A., Oliveira, J. A., & Ferreira JR. Comparative analysis of stingless bee honey from Venezuela and Brazil. Ethiop J Biol Sci. 2021;18(1):67–75.

24. Tey, S. L., Tan, K. W., & Lee CY. Quality assessment of stingless bee honey from Southeast Asia. Food Chem. 2022;370.:131045.

25. Stramm KM. Composition and quality of honey bee Jandaira (Melipona subnitida), storage effects and comparison with Apis mellifera honey . 2011.

26. Živkov Baloš M, Popov N, Vidaković S, Ljubojević Pelić D, Pelić M, Mihaljev Ž, et al. Electrical conductivity and acidity of honey. Arch Vet Med. 2018;11(1):91–101.

27. Souza, Roubik, Barth Et Al. Composition of Stingless bee honey: setting quality standards, Interciencia. 2006;31(12).

28. Al-Tikrity, W. S., Benton, A. W., Hillmann, R. C., & Clarke WW. The relationship between the amount of HMF in honey and its fermentation. Am Bee J. 1989;129(7):478–81.

29. Almasaudi, S. B., El-Shitany, N. A., Abbas, A. T., Abdel-dayem, U. A., Ali, S. S., Al Jaouni, S. K., & Harakeh S. Antioxidant, anti-inflammatory, and antiulcer potential of manuka honey against gastric ulcer in rats. Oxid Med Cell Longev. 2017;

30. Majtan, J., Bohova, J., Horniackova, M., Klaudiny, J., & Majtan V. Anti-biofilm effects of

honey against wound pathogens Proteus mirabilis and Enterobacter cloacae. Phyther Res. 2014;28(1):69–75.

31. Kilty, S. J., Duval, M., Chan, F. T., Ferris, W., & Slinger R. Methylglyoxal: (active agent of manuka honey) in vitro activity against bacterial biofilms. Int Forum Allergy Rhinol. 2011;1(5):348–50.

32. Teferi Damto DK, Meseret. Physico-chemical and microbiological characteristics of honey produced by stingless bees. 2022;

33. Vit, Patricia & Oddo, Livia & Marano, Maria & Mejias E. Venezuelan stingless bee honeys characterized by multivariate analysis of physicochemical properties. Apidologie. 1998;(29):377–89.

34. F.C. Biluca, F. Della Betta, G.P. de Oliveira, L.M. Pereira, L.V. Gonzaga, A.C.O. Costa RF. 5-HMF and carbohydrates content in stingless bee (Meliponinae spp.) honey by CE before and after thermal treatment. Food Chem. 2014;159:244–9.

35. Fabíola Carina Biluca, Francieli Braghini, Luciano Valdemiro Gonzaga, Ana Carolina Oliveira Costa, Roseane Fett. Physicochemical profiles, minerals and bioactive compounds of stingless bee honey (Meliponinae), Journal of Food Composition and Analysis. J Food Compos Anal. 2016;50(0889–1575):61–9.

36. Vit P, Roubik DW, Pedro SRM. Pot-Honey: A legacy of stingless bees. Pot-Honey: A Legacy of Stingless Bees. 2012. 1–654 p.

37. J.A. Nweze, J. Okafor, E.I. Nweze JEN. Evaluation of physicochemical and antioxidant properties of two stingless bee honeys: a comparison with Apis mellifera honey from Nsukka, Nigeria. BMC Res Notes. 2017;10(566).

38. Quality and Standards Authority of Ethiopia (QSAE). ET 1202: Honey - Specification

[Internet]. 2005. Available from:
https://archive.org/search?query=subject%3A%22public.resource.org%22

39. Quality and Standards Authority of Ethiopia (QSAE). Quality and Standards Authority of Ethiopia (QSAE) Honey Specification: Ethiopian Standard, ES 1202. 2005;

40. Souza ECA, Menezes C, Flach A. Stingless bee honey (Hymenoptera, Apidae, Meliponini): a review of quality control, chemical profile, and biological potential. Apidologie. 2021;52(1):113–32.

41. Bekele Tesfaye, Deaslegn Begna ME. Evaluation of Physicochemical Properties of Honey Produced In Bale Natural Forest, Southeastern Ethiopia. Int J Agric Sci Food Technol. 2016;1(2).

42. Bogdanov S, Jurendic T, Sieber R, Gallmann P. Honey for Nutrition and Health: A Review. J Am Coll Nutr. 2009 Jan 1;27:677–89.

43. Silva JMC, Barbosa LCF, Leal IR TM. The Caatinga: understanding the challenges. In: Silva JMC, Leal IR, Tabarelli M (eds) Caatinga: the largest tropical dry forest region in South America. Springer, Cham. 2017;3–19.

44. Alves DA, Imperatriz-Fonseca VL, Francoy TM, Santos-Filho PS, Bil-len J WT. Successful maintenance of a stinglessbee population despite a severe genetic bottleneck. Conserv Genet. 2011;2:2:647–658.

45. Mushonga B, Hategekimana L, Habarugira G, Kandiwa E, Samkange A, Ernest Segwagwe BV. Characterization of the Beekeeping Value Chain: Challenges, Perceptions, Limitations, and Opportunities for Beekeepers in Kayonza District, Rwanda. Adv Agric. 2019;2019.

46. S.A. Meo, S.A. Al-Asiri, A.L. Mahesar MJA. Role of honey in modern medicine. Saudi J

Biol Sci. 2017;24(5):975–8.

47. Nnadi PC. Determination and Comparative Evaluation of Mineral Content of Honey obtain from Rivers State University, Forestry Arboretum and Port Harcourt Local Market. J Appl Sci Environ Manag. 2020;24(9):1593–7.

48. Anklam E. A Review of the Analytical Methods to Determine the Geographical and Botanical Origin of Honey. Food Chem. 1998;63:549–62.

49. Machado De-Melo AA, Almeida-Muradian LB de, Sancho MT, Pascual-Maté A. Composición y propiedades de la miel de Apis mellifera: una revisión. J Apic Res. 2018;57(1):5–37.

50. Downey, G., Fouratier, V., & Kelly JD. A Review of the Analytical Methods to Determine the Geographical and Botanical Origin of Honey. J Agric Food Chem. 2003;51(2):347–54.

51. Karabagias IK, Karabournioti S, Karabagias VK, Badeka A V. Palynological, physico-chemical and bioactivity parameters determination, of a less common Greek honeydew honey: "dryomelo." Food Control [Internet]. 2020;109:106940. Available from: https://www.sciencedirect.com/science/article/pii/S0956713519305298

52. Bogdanov S, D'Arcy BR, Mossel B, Marcazzan GL. Honey quality and international regulatory standards : review by the International Honey International honey standards are specified in a European Honey. Bee world. 1999;80(2):61–9.

53. Tan, K. W., Lim, H. P., & Lee CY. Physicochemical properties of honey from stingless bees in Malaysia. J Apic Res. 2021;60(1):45-54.

54. Couvillon MJ, Wenseleers T, Imperatriz-Fonseca VL, Nogueira-Neto P, Ratnieks FLW. Comparative study in stingless bees (Meliponini) demonstrates that nest entrance size

predicts traffic and defensivity. J Evol Biol. 2008;21(1):194–201.

55. Zulkhairi Amin FA, Sabri S, Mohammad SM, Ismail M, Chan KW, Ismail N, et al. Therapeutic properties of stingless bee honey in comparison with european bee honey. Adv Pharmacol Sci. 2018;2018.

56. Kiprono SJ, Mengich G, Kosgei J, Mutai C, Kimoloi S. Ethnomedicinal uses of stingless bee honey among native communities of Baringo County, Kenya. Sci African [Internet]. 2022;17:e01297. Available from: https://doi.org/10.1016/j.sciaf.2022.e01297

57. Sabella JK, Gladys M, Bartholomew NO, Charles M, Sammy K. Therapeutic uses of stingless bee honey by traditional medicine practitioners in Baringo County, Kenya. J Pharmacogn Phyther. 2022;14(3):27–36.

58. Bogdanov S, Vit P, Kilchenmann V. Sugar profiles and conductivity of stingless bee honeys from Venezuela. Apidologie. 1996;27(6):445–50.

Response to Reviewers

PONE-D-24-28380R1

Physicochemical properties of stingless bees (*meliponula beccarii*) honey in Dandi and Meta Robi districts of West Shewa zone, Ethiopia

PLOS ONE

---

6. Review Comments to the Author

Please use the space provided to explain your answers to the questions above. You may also include additional comments for the author, including concerns about dual publication, research ethics, or publication ethics. (Please upload your review as an attachment if it exceeds 20,000 characters)

Reviewer #1: MINOR CORRECTIONS: Please highlight these changes in RED/BLUE in your corrected manuscript

a) Introduction Section:
Please include a brief mention of Meliponula beccarii as an important stingless bee species in Ethiopia, with appropriate references. Highlight that this species is commonly found in the Dandi and Meta Robi districts of the West Shewa zone, Ethiopia.

Meliponula beccarii, a species of stingless bee, is commonly found in Ethiopia and is well known for its honey production as well as its contributions to local biodiversity and pollination. It is particularly prevalent in the Dandi and Meta Robi districts of the West Shewa zone, where it plays a vital role in the ecosystem.

b) Results and Discussion Section:

(i) Figure 2: The current image of the water bottle is not ideal. We need to clearly see the color of the honey (green/yellow/dark). Please describe the color more clearly and replace the image with a better one.

The image of the water bottle removed

(ii) Please be cautious with the phrase “to indicate medicinal value of honey.” It would be more appropriate to say: “indicate the medicinal and nutritional value of honey.” Kindly check and revise this. Corrected accordingly (on page 10 of the revised manuscript)

(iii) The discussion on the sugar profile needs further elaboration and additional citations (Please compare with other findings the data from Stingless Bee as well). The

comparison with the Codex Alimentarius standard is very appropriate maintain as it is, BUT should also include comparisons with stingless bee honey from other regions like Southeast Asia (Malaysia, Indonesia, Thailand) and South America- Brazil/Venezuela etc..
I recommend comparing your results with the standards for stingless bee honey from other countries. Please refer to existing standards for stingless bee honey in these regions (IF ANY).

Others are OK!

Thank you for your constructive comments on our manuscript. We greatly appreciate your input, as it has helped us enhance the quality of our work.

In addition to addressing your suggestions, we have included comparisons for each physicochemical parameter with stingless bee honey from other countries in Southeast Asia and South America in the table and body of the revised manuscript. We believe this additional context strengthens our findings.

Reviewer #2:

Abstract
1. Check maltose content result because it is extremely high .compare with other findings

Previous studies has reported maltose levels in stingless bee honey generally range from lower values, typically around 1% to 5% (22,23). However, the high maltose content in our stingless bee honey may be due to several factors like specific floral sources can significantly influence sugar composition, with certain plants containing higher maltose concentrations (22), fermentation processes within the nest may also increase maltose levels if honey is stored for extended periods (23), the foraging behavior of stingless bees that could lead to the preferential collection of nectar from maltose-rich plants and environmental factors, such as soil composition and climate, that may further impact floral diversity and nectar quality, resulting in elevated maltose levels (53), processing and storage methods might alter sugar profiles, with prolonged storage or heat affecting composition (24), variability among stingless bee species (23).

2.Again I inform the authors that high acidity (Free acidity) is not related with quality and medicinal properties .High acidity of stingless bee related to fermentation rate of this honey because stingless bee honey contain high moisture and alcohol is easily formed in it .that is way free acidity is high.

High quality and medicinal properties of stingless bee is related to strong pH and high antioxidant properties.

Higher free acidity in honey can enhance flavor complexity, inhibit microbial growth to extend shelf life (Tan et al., 2021), and provide potential health benefits, including antibacterial properties from organic acids (Tey et al., 2022).

3. The result of turanose was not presented in abstract:

Included in the abstract of the revised manuscript

Materials and methods: In the M&M, the analysis of turanose was similar as fructose, glucose, and sucrose and already stated as "A standard curve was used to determine sugar content (fructose, glucose, sucrose, turanose, and maltose) and the values were calculated as gram/100gram of honey sample"

Results and Discussion

4. Please free acidity more acceptable than acidity on sub heading (corrected accordingly)

5. The authors stated that free acidity was extremely high in this study and also mentioned this is because of different species and agro ecologies. But since different study was conducted in West Shoa as the author stated in introduction, why you don't compare you're finding with those study conducted in same area and same country. The study lack literature discussion in Ethiopia.

Or high acidity because of your sample impurity? Please discuss carefully on the finding. I didn't see improvement.

Reports from Ethiopia has been included and discussed well with plausible justifications added for why free acidity was high in our honey sample in the manuscript

6. The line and paragraph spacing and font color is not uniform in all paragraph (corrected accordingly)

7. Correct sub heading of moisture content (corrected accordingly)

8. Font color and size of sub heading should be improved ((corrected accordingly)

9. In abstract the authors stated elevated pH value but in results and discussion parts, the finding of the study is similar with other finding conducted in Ethiopia. Revise abstract or discussion parts. The abstract part revised.

10. The arrangement of the paragraph is week

11. For me discussion of sugar profile is not enough? Needs to be revised again by comparing the finding of the study with other similar findings. Well discussed this time in the revised manuscript

12. Under HMF paragraph please correct the paragraph (corrected)

13. Why fresh collected and marketed ones was not compared in finding. It is better if both types of samples was compared parallel. Both types of samples were compared parallel at every parameter

Conclusion

14.“This study evaluated This study evaluated “. Please remove redundant sentence (Removed accordingly)

15.” This study evaluated the physicochemical properties of honey produced by the ground-nesting stingless bee (Meliponula beccarii) in Ethiopia” , by collecting sample from two woreda of Oromia region we can say this study represent Ethiopia. Please revise this one again. (Revised)

165 .revise the conclusion based on your fining especially on fresh and marketed honey sample variability (revised)

176. lack of consistent formatting of reference: We utilized the Mendeley desktop reference manager to format our references according to the Vancouver style outlined in the PLOS author guidelines. We believe this will ensure consistent formatting throughout the reference section. Additionally, we have made adjustments to enhance the presentation and engage the reviewer more effectively.
