# Supplementary material for: Physicochemical properties of stingless bees (Meliponula beccarii) honey in Dandi and Meta Robi districts of West Shewa zone, Ethiopia
Source: PLoS One. 2024 Dec 12;19(12):e0311725. doi: 10.1371/journal.pone.0311725 (PMC11637301; doi:10.1371/journal.pone.0311725)
Supplement: Attachment — Submitted filename: comment to author.docx [file pone.0311725.s003.docx]

**Comment to author**

Title

1. Title should be modified? It say properties of stingless bee, what properties? There are a lot of properties. Please specify that properties

2. It is not necessary to write **Title: Properties of stingless bees (Meliponula beccarii) honey in Dandi and Meta Robi districts of West Shewa zone, Ethiopia. Starting by name of the article title is possible**

3. I strongly recommend the authors if follow the journal formatting guidelines starting from Title formatting to Reference writing since the manuscript didn’t prepared based on the journal guidelines

4 .correct affiliation and corresponding author according to journal formatting guidelines

Abstact

5. Be careful for terminology of the parameter. For example free acidity not acidity , electrical

Conductivity not electric conductivity

6.put correct standard unit for each parameter in abstract .example free acidity (meq/ Kg) not mill./kg, mS/cm not	ms/cm

7. put SI for moisture content

8. I recommend ash content not mineral .

9. “The distinctive low HMF, high acidity and low pH values of the stingless bee honey of this study may represent freshness and good quality of the honey and indicative medicinal potential” . I didn ’t think high acidity indicate fresheness of honey, in this conntarst high acidity indicate fermentation rate of honey . the free acidity of obtained in this extremely high as compared to previous study conducted in west shoa and other parts of the country ,this need strong justification .may be during sample collecton orr harvesting mixing sample with soil,pollen ,and other dead and imputirtes cause honey to be fermented and high acidity

10.” This study evaluated physicho-chemical properties of honey produced by ground nesting stingless

bee (Meliponula beccarii) in two districts of West Shewa zone,” this is redundant since the author mentioned in firstyt line of abstract .please remove and re –edit	again

11.what cause your study unique since different study was reported in west shoa.

***Introduction***

11.please folloew journal juideline starting from heading ,

12. reference citation is in number form for this journal.

13 .The name of *Meliponula beccarii* must be in italized form

14. if damma daamu local name it must be in italic form

15. Previous work and limitation /gap on characterization of stingless bee honey in that area as well as in Ethiopia must be clearly indicated

16. Paragraph arrangement of the introduction is not good

**Materials and method**

17. I recommend if there is sub heading under materials and methods section, unless this arrangement confuse the reader

18. Figure 2, especially on the bottle containing sample is not visible, remove or replace with good picture

**Result and discussion**

19. on the result obtained on the free acidity , I strongly doubt on the result obtained (338.79 ± 72.46) . what is reason behind extremely high free acidity , is soil and other impurities is mixed in to honey during honey collection or harvesting .the author must discuss and compare with other data in Ethiopia.

20. I recommend the author also to compare the result with standard stingless bee honey in other countries (like brazil,venzuala and etc.) ,please refer there is standard of stingless bee honey in other country.)

21. Under acidity section , the author mentioned that sample was purchased .However they didn’t mentioned in methodology part . Sample collection should clearly indicated under methodology part otherwise it confuse the reader

22. CORRECT 3.1 Acidity to Free acidity

23. *The overall mean moisture content of the 24 stingless bee honey samples was 23.45-36.75% is not good sentence .rewrite again*

24.Table 1 should be come after paragraph of free acidity .

25.The paragraph arrangement is not good .it must be justified .

26. **This finding is consistent with previous studies. Gela et al. (2021) reported an overall pH range of 3.4-3.9 (mean 3.7±0.15) for stingless bee honey from four districts in Ethiopia**. Remove full stop between studies and Gela.

27 . The standard pH value for Apis mellifera honey is 3.42–6.10 (Tesfaye et al., 2016*).* Are you sure with sentence or statement. In my opinion, 6.10 is not standard value of pH . Be sure also the citation .revise again.

28 . what your sample size . under materials and method s the author mentioned that 24 sample was collected .But on the table 1 the author stated that the sample size(N)=27. Which one is correct? It need revision

29 . Put acronomy for all abbreviation found in the table.

30. The way of data presentation is not good for this article. I recommend the authors to use standard table or journal guidelines. Additionally, I recommend to put the result by mean ± std with mean separation letters .I observe level of significance in body of manuscript .but there is no in a table .How?. Please rewrite again.

30. 3.3 *Mineral (ash) contents* correct to Ash content only remove mineral

31. Under 3.3. I recommend the authors to start the paragraph by presenting the result not discussion with literature .Remove first sentence

32. *M.beccarii* should be in italic form in all manuscript

33.Be carefull when reporting the facts .for example “The higher mineral content of the stingless bee honey from the Dandi and Meta Robi districts indicates that it may be a better choice for both medicinal and nutritional purposes, provided that the honey is properly standardize” . I don’t think so that higher mineral content indicate medicinal value of honey .Rather there is parameters that indicate medicinal and nutritional value of honey. Please revise and cite the above sentence.

34. under 3.4 Sugar profile need more discussion and citation . Not enough now. Compare current result with standards of stingless bee honey in other country.

35. under 3.5 plesae start the paragragh by mentioning the obtained result .discussion come after presenting the finding.

36. In this study, 0.06 and 4.2.0 values were recorded as minimum and maximum. *4.2.0 correct this one.*

37 . Please use style uniform typing for et al . in some areas it is in italic form and in some areas it is non- italic

**Conclusion**

**38 . how high free acidity related to potentiality of medicinal value .**

**39. In conclusion I need a finding related both source of sample collected results**
