# Supplementary material for: Physicochemical properties of stingless bees (Meliponula beccarii) honey in Dandi and Meta Robi districts of West Shewa zone, Ethiopia
Source: PLoS One. 2024 Dec 12;19(12):e0311725. doi: 10.1371/journal.pone.0311725 (PMC11637301; doi:10.1371/journal.pone.0311725)
Supplement: Attachment — Submitted filename: Response1 to the Reviewers.docx [file pone.0311725.s004.docx]

PONE-D-24-28380

Properties of stingless bees (Meliponula beccarii) honey in Dandi and Meta Robi districts of West Shewa zone, Ethiopia

PLOS ONE

Dear Dr. Begna,

Thank you for submitting your manuscript to PLOS ONE. After careful consideration, we feel that it has merit but does not fully meet PLOS ONE’s publication criteria as it currently stands. Therefore, we invite you to submit a revised version of the manuscript that addresses the points raised during the review process.

Please submit your revised manuscript by Sep 09 2024 11:59PM. If you will need more time than this to complete your revisions, please reply to this message or contact the journal office at [plosone@plos.org](mailto:plosone@plos.org). When you're ready to submit your revision, log on to <https://www.editorialmanager.com/pone/> and select the 'Submissions Needing Revision' folder to locate your manuscript file.

Please include the following items when submitting your revised manuscript:

- A rebuttal letter that responds to each point raised by the academic editor and reviewer(s). You should upload this letter as a separate file labeled 'Response to Reviewers'.
- A marked-up copy of your manuscript that highlights changes made to the original version. You should upload this as a separate file labeled 'Revised Manuscript with Track Changes'.
- An unmarked version of your revised paper without tracked changes. You should upload this as a separate file labeled 'Manuscript'.

If you would like to make changes to your financial disclosure, please include your updated statement in your cover letter. Guidelines for resubmitting your figure files are available below the reviewer comments at the end of this letter.

If applicable, we recommend that you deposit your laboratory protocols in [protocols.io](http://protocols.io/) to enhance the reproducibility of your results. Protocols.io assigns your protocol its own identifier (DOI) so that it can be cited independently in the future. For instructions see: <https://journals.plos.org/plosone/s/submission-guidelines#loc-laboratory-protocols>. Additionally, PLOS ONE offers an option for publishing peer-reviewed Lab Protocol articles, which describe protocols hosted on [protocols.io](http://protocols.io/). Read more information on sharing protocols at <https://plos.org/protocols?utm_medium=editorial-email&utm_source=authorletters&utm_campaign=protocols>.

We look forward to receiving your revised manuscript.

Kind regards,

Tzen-Yuh Chiang
Academic Editor
PLOS ONE

Journal Requirements:

When submitting your revision, we need you to address these additional requirements.

Table in response to editor’s comments

| **No** | **Comments** | **Provided Responses** |
| --- | --- | --- |
| 1 | ensure that your manuscript meets PLOS ONE's style requirements | Already done as it meets PLOS ONE's style requirements |
| 2 | In your Methods section, please provide additional information regarding the permits you obtained for the work | no permits were required, and a brief statement explaining why is presented in the manuscript |
| 3 | The corresponding author must be affiliated with the chosen institute for billing options | remove this option |
| 4 | Figure 1 in the submission contains map images that may be copyrighted | The country’s map is public and is not copyrighted. The study sites/districts are created by the study team themselves based on their coordinates and have no copyright issues. The fig.1 can be removed |
| 5 | copy of Table 2 | Unnecessary included in pare 21 of the pdf and removed |
| 6 |  |  |
| 7 |  |  |
| 8 |  |  |
| 9 |  |  |

1. Please. The PLOS ONE style templates can be found at

<https://journals.plos.org/plosone/s/file?id=wjVg/PLOSOne_formatting_sample_main_body.pdf> and 
<https://journals.plos.org/plosone/s/file?id=ba62/PLOSOne_formatting_sample_title_authors_affiliations.pdf>

2. In your Methods section, please provide additional information regarding the permits you obtained for the work. Please ensure you have included the full name of the authority that approved the field site access and, if no permits were required, a brief statement explaining why.

3. Please note that in order to use the direct billing option the corresponding author must be affiliated with the chosen institute. Please either amend your manuscript to change the affiliation or corresponding author, or email us at [plosone@plos.org](mailto:plosone@plos.org) with a request to remove this option.

4. We note that Figure 1 in your submission contains map images that may be copyrighted. All PLOS content is published under the Creative Commons Attribution License (CC BY 4.0), which means that the manuscript, images, and Supporting Information files will be freely available online, and any third party is permitted to access, download, copy, distribute, and use these materials in any way, even commercially, with proper attribution. For these reasons, we cannot publish previously copyrighted maps or satellite images created using proprietary data, such as Google software (Google Maps, Street View, and Earth). For more information, see our copyright guidelines: <http://journals.plos.org/plosone/s/licenses-and-copyright>.
 
We require you to either (1) present written permission from the copyright holder to publish these figures specifically under the CC BY 4.0 license, or (2) remove the figures from your submission:
 
1) You may seek permission from the original copyright holder of Figure 1 to publish the content specifically under the CC BY 4.0 license.  
 
We recommend that you contact the original copyright holder with the Content Permission Form (<http://journals.plos.org/plosone/s/file?id=7c09/content-permission-form.pdf>) and the following text:
“I request permission for the open-access journal PLOS ONE to publish XXX under the Creative Commons Attribution License (CCAL) CC BY 4.0 (<http://creativecommons.org/licenses/by/4.0/>). Please be aware that this license allows unrestricted use and distribution, even commercially, by third parties. Please reply and provide explicit written permission to publish XXX under a CC BY license and complete the attached form.”
 
Please upload the completed Content Permission Form or other proof of granted permissions as an ""Other"" file with your submission.
 
In the figure caption of the copyrighted figure, please include the following text: “Reprinted from [ref] under a CC BY license, with permission from [name of publisher], original copyright [original copyright year].”
 
2) If you are unable to obtain permission from the original copyright holder to publish these figures under the CC BY 4.0 license or if the copyright holder’s requirements are incompatible with the CC BY 4.0 license, please either i) remove the figure or ii) supply a replacement figure that complies with the CC BY 4.0 license. Please check copyright information on all replacement figures and update the figure caption with source information. If applicable, please specify in the figure caption text when a figure is similar but not identical to the original image and is therefore for illustrative purposes only.

The following resources for replacing copyrighted map figures may be helpful:
 
USGS National Map Viewer (public domain): <http://viewer.nationalmap.gov/viewer/>
The Gateway to Astronaut Photography of Earth (public domain): <http://eol.jsc.nasa.gov/sseop/clickmap/>
Maps at the CIA (public domain): <https://www.cia.gov/library/publications/the-world-factbook/index.html> and <https://www.cia.gov/library/publications/cia-maps-publications/index.html>
NASA Earth Observatory (public domain): <http://earthobservatory.nasa.gov/>
Landsat: <http://landsat.visibleearth.nasa.gov/>
USGS EROS (Earth Resources Observatory and Science (EROS) Center) (public domain): [http://eros.usgs.gov/#](http://eros.usgs.gov/)
Natural Earth (public domain): <http://www.naturalearthdata.com/>

5. Please include a copy of Table 2 which you refer to in your text on page 21 (in PDF format).


[Note: HTML markup is below. Please do not edit.]

Reviewers' comments:

Reviewer's Responses to Questions

**Comments to the Author**

1. Is the manuscript technically sound, and do the data support the conclusions?

The manuscript must describe a technically sound piece of scientific research with data that supports the conclusions. Experiments must have been conducted rigorously, with appropriate controls, replication, and sample sizes. The conclusions must be drawn appropriately based on the data presented.

Reviewer #1: Yes

Reviewer #2: Yes

Reviewer #3: No

2. Has the statistical analysis been performed appropriately and rigorously?

Reviewer #1: Yes

Reviewer #2: N/A

Reviewer #3: Yes

3. Have the authors made all data underlying the findings in their manuscript fully available?

The [PLOS Data policy](http://www.plosone.org/static/policies.action#sharing) requires authors to make all data underlying the findings described in their manuscript fully available without restriction, with rare exception (please refer to the Data Availability Statement in the manuscript PDF file). The data should be provided as part of the manuscript or its supporting information, or deposited to a public repository. For example, in addition to summary statistics, the data points behind means, medians and variance measures should be available. If there are restrictions on publicly sharing data—e.g. participant privacy or use of data from a third party—those must be specified.

Reviewer #1: Yes

Reviewer #2: No

Reviewer #3: Yes

4. Is the manuscript presented in an intelligible fashion and written in standard English?

PLOS ONE does not copyedit accepted manuscripts, so the language in submitted articles must be clear, correct, and unambiguous. Any typographical or grammatical errors should be corrected at revision, so please note any specific errors here.

Reviewer #1: Yes

Reviewer #2: Yes

Reviewer #3: No

5. Review Comments to the Author

Please use the space provided to explain your answers to the questions above. You may also include additional comments for the author, including concerns about dual publication, research ethics, or publication ethics. (Please upload your review as an attachment if it exceeds 20,000 characters)

Table-2response to reviwer-1 comments

| **No** | **Comments** | **Provided Responses** |
| --- | --- | --- |
|  | General |  |
| 1 | the research highlights significant findings such as low hydroxymethylfurfural (HMF) levels, high acidity, and low pH, which indicate the honey's freshness and potential medicinal benefits but not specifically mention its medicinal properties | Stingless bee honey exhibits superior antimicrobial, antioxidant, anti-inflammatory, and wound healing properties compared to European bee honey, due to its greater acidity, lower carbohydrates, and higher antioxidant activity, treats diabetes, bronchitis, mycosis, throat aches, and sexual impotence, as well as an antidote against snakebites or rabid dog bites  (Further included in the manuscript body on page 14) |
|  | Methods |  |
| 2 | Why do you choose these two location? any reason? And please mark clearly on the MAP, showing specific coordinate. Is the sample collected from wild or any private company running the Collection business? | The Dandi and Meta Robi districts were selected for this study because other districts in the zone have been previously studied, while these two districts lack information on the Physicochemical properties of stingless bee honey, despite their potential for stingless honey production and marketing. The specific coordinates of the sampling locations in these two districts are presented. All the honey samples were collected from feral stingless bee nests through nest excavations, except the samples purchased for comparisons |
|  | Results |  |
| 3 | Make sure name of genus and species in italics | Done according to the comments |
| 4 | Table 1 and sections 3.1 to 3.6 must be properly Tabulated. Make sure unit numbers standard | Corrected accordingly that are reflected in the text body |
| 5 | standards according to the Codex Alimentarius Standard for Honey (CODEX STAN 12-1981, Rev.2-2001) | Provided in the manuscript |
| 6 | Make sure name of genus and species in italics | Corrections done where they are |
| 7 | Table 1 and sections 3.1 to 3.6 must be properly Tabulated. Make sure unit numbers standard | standards according to the Codex Alimentarius Standard for Honey (CODEX STAN 12-1981, Rev.2-2001) were presented accordingly that are reflected in the text body |
| 8 | Please discuss the unique findings of data found in your two honeys comparing to others | Presented in the discussion section as “The unique findings from the data are that the stingless bee (*Meliponula beccarii*) honey from the Dandi and Meta Robi districts of Ethiopia exhibits high acidity, low pH, low HMF, and a distinctive sugar profile, differentiating it from other places studied stingless bee honey (4) indicating potential medicinal properties”. |
| 9 | improve conclusion section taking consideration of improvement from the Results/Discussion | Improved as per the suggestion (reflected in the manuscript) |

Reviewer #1: General about the paper:
This study investigates the physicochemical properties of honey produced by the underground nesting stingless bee (Meliponula beccarii) in the Dandi and Meta Robi districts of West Shewa, Ethiopia. By analyzing 24 honey samples, the research highlights significant findings such as low hydroxymethylfurfural (HMF) levels, high acidity, and low pH, which indicate the honey's freshness and potential medicinal benefits but not specifically mention its medicinal properties. Main differences with its in glucose, acidity, moisture content, and pH between the two districts suggesting the honey's unique qualities between 2 places, Honey produced by M. beccarii is valuable for the traditional medicine BUT no data presented in this study, and warranting further investigation.

- Stingless bee honey exhibits superior antimicrobial, antioxidant, anti-inflammatory, and wound healing properties compared to European bee honey, due to its greater acidity, lower carbohydrates, and higher antioxidant activity (1), treats diabetes, bronchitis, mycosis, throat aches, and sexual impotence, as well as an antidote against snakebites or rabid dog bites (2,3).
- The authors recommend further research to explore the specific medicinal properties and potential health benefits of honey produced in the study areas as well as in the country in general, investigate the factors contributing to differences in physicochemical properties between the stingless honey produced in different localities, and establish a more comprehensive understanding of the medicinal applications of this honey based on its unique characteristics.
- Methods:
  Why do you choose these two location? any reason? And please mark clearly on the MAP, showing specific coordinate. Is the sample collected from wild or any private company running the Collection business?
  The Dandi and Meta Robi districts were selected for this study because other districts in the zone have been previously studied, while these two districts lack information on the Physicochemical properties of stingless bee honey, despite their potential for stingless honey production and marketing. The specific coordinates of the sampling locations in these two districts are presented. All the honey samples were collected from feral stingless bee nests through nest excavations, except the samples purchased for comparisons.

RESULTS
a) Make sure name of genus and species in italics. Corrections done where they are
b) Table 1 and sections 3.1 to 3.6 must be properly Tabulated. Make sure unit numbers standard. It will be easier to compare to the paper published by Nordin et al., (2018) about Stingless bee data (Codex alimnetarius standard for normal sting Bee; we do not have standard for Stingless Bee,). standards according to the Codex Alimentarius Standard for Honey (CODEX STAN 12-1981, Rev.2-2001) were presented accordingly that are reflected in the text body

c) Please discuss the unique findings of data found in your two honeys comparing to others. This not properly highlight in this section.The unique findings from the data are that the stingless bee (*Meliponula beccarii*) honey from the Dandi and Meta Robi districts of Ethiopia exhibits high acidity, low pH, low HMF, and a distinctive sugar profile, differentiating it from other places studied stingless bee honey (4) indicating potential medicinal properties.
Conclusion:
Please improve this section taking consideration of improvement from the Resulst/Discussion.

Based on the results of this study, the stingless bee (Meliponula beccarii) honey from the Dandi and Meta Robi districts of West Shewa, Ethiopia exhibits several distinctive physicochemical properties compared to typical Apis honey in other places and species studied stingless bee honey. The honey had an overall mean acidity of 306.64 ± 87.95 meq./kg, which is considered quite high, as well as a relatively high moisture content with an average of 28.05 ± 3.52%. The honey also had a low pH, with a mean of 3.29 ± 0.16, and a very low hydroxymethylfurfural (HMF) content of 0.89 ± 1.14 mg/kg, indicating its freshness. Additionally, the mineral content, as measured by ash, was low at 0.63 ± 0.24g, and the sugar profile was unique, with higher levels of fructose and maltose compared to glucose and sucrose. These distinctive physicochemical characteristics suggest that the stingless bee honey may have potential medicinal and quality attributes that differentiate it from commercially available Apis honey. The authors recommend further detailed studies on the medicinal properties and values of this stingless bee honey, as well as other ground-nesting and stingless bee species, to provide scientific evidence for its traditional use and uncover its potential benefits and contributions to traditional medicine and apiculture.

Reviewer #2: Title

Table-2 response to reviwer-2 comments

| **No** | **Comments** | **Provided Responses** |
| --- | --- | --- |
|  | General |  |
| 1 | Modify the Title | Modified as *“Physicochemical properties of stingless bees (meliponula beccarii) honey in Dandi and Meta Robi districts of West Shewa zone, Ethiopia”* |
| 2 | Remove Title | Removed |
| 3 | follow the journal formatting guidelines | Followed in the revised manuscript |
|  | Abstract |  |
| 5 | care for terminology of the parameter. For example free acidity not acidity , electrical Conductivity not electric conductivity | Corrected in the revised manuscript |
| 6 | put correct standard unit for each parameter in abstract .example free acidity (meq/ Kg) not mill./kg, mS/cm not ms/cm | (Corrected in the revised manuscript) |
| 7 | put SI for moisture content: | The SI (International System of Units) unit for moisture content is percent (%) by weight (w/w). corrected in the revised manuscript |
| 8 | I recommend ash content not mineral | correction accepted and corrected accordingly |
| 9 | The distinctive low HMF, high acidity and low pH values of the stingless bee honey of this study may represent freshness and good quality of the honey and indicative medicinal potential” . I didn ’t think high acidity indicate fresheness of honey, in this conntarst high acidity indicate fermentation rate of honey . the free acidity of obtained in this extremely high as compared to previous study conducted in west shoa and other parts of the country ,this need strong justification .may be during sample collecton orr harvesting mixing sample with soil,pollen ,and other dead and imputirtes cause honey to be fermented and high acidity | The acidity levels in stingless bee honeys can vary significantly based on factors like bee species, geographical origin, and other variables. The acidity values were reported 26.5 to 66.4 meq/kg for *Melipona scutellaris* honey (Souza et al., 2006), 29.7 ± 4.4 meq/kg for *Plebeia saiqui* honey, and 42.7 ± 4.2 meq/kg for *Tetragonisca angustula* honey (Vit et al., 1998), and 22.4 to 34.6 mEq/kg for *Melipona beecheii* honey (Ruiz-Argueso and Rodriguez-Navarro, 1973). The high acidity and low pH observed in the *Meliponula beccarii* honey studied appear to be unusually high. This suggests the high acidity may be due to increased fermentation due to differences in bee species and geographical origin (Souza et al., 2006), improper handling and processing (Al-Tikrity et al., 1989), and high moisture content (Al-Tikrity et al., 1989).  Corrected in the revised manuscript |
| 10 | This study evaluated physicho-chemical properties of honey produced by ground nesting stinglessbee (Meliponula beccarii) in two districts of West Shewa zone,” this is redundant since the author mentioned in firstyt line of abstract .please remove and re –edit again | Corrected by removing the redundant sentence |
| 11 | what cause your study unique since different study was reported in west shoa | The unique findings from the data are that the stingless bee (*Meliponula beccarii*) honey from the two districts exhibits high acidity, low pH, low HMF, and a distinctive sugar profile, differentiating it from other places studied stingless bee honey indicating potential medicinal properties” |
|  | Introduction |  |
| 12 | follow journal guidelines | Suggestions accepted and abide |
| 13 | reference citation is in number form for this journal | Suggestions accepted and abide |
| 14 | The name of Meliponula beccarii must be in italized form | Suggestions accepted and abide |
| 15 | Previous work and limitation /gap on characterization of stingless bee honey in that area as well as in Ethiopia must be clearly indicated | Suggestions accepted and corrections done in the revised manuscript |
| 16 | Paragraph arrangement of the introduction is not good | Suggestions accepted and corrections done in the revised manuscript |
|  | Metherials and methods (M&M) |  |
| 17 | I recommend if there is sub heading under materials and methods section, unless this arrangement confuse the reader | Suggestions were accepted and corrections on the su-heading were done in the M&M parts of the revised manuscript |
| 18 | Figure 2, especially on the bottle containing sample is not visible, remove or replace with good picture | The invisible bottles bottles removed and replaced with the visible one |
|  | Result and discussion |  |
| 19 | on the result obtained on the free acidity, I strongly doubt on the result obtained (338.79 ± 72.46) . what is reason behind extremely high free acidity , is soil and other impurities is mixed in to honey during honey collection or harvesting .the author must discuss and compare with other data in Ethiopia | The high acidity and low pH observed in the *Meliponula beccarii* honey studied appear to be unusually high. This suggests the high acidity may be due to increased fermentation due to differences in bee species and geographical origin (Souza et al., 2006), improper handling and processing (Al-Tikrity et al., 1989), and high moisture content (Al-Tikrity et al., 1989). |
| 20 | I recommend the author also to compare the result with standard stingless bee honey in other countries (like brazil,venzuala and etc.) ,please refer there is standard of stingless bee honey in other country.) | this is already discussed in the revised manuscript of free acidity section as “The *Meliponula beccarii* honey studied appears to have unusually high acidity and low pH compared to stingless bee honeys from Brazil (Melipona scutellaris), Venezuela (Plebeia saiqui, Tetragonisca angustula), and Mexico (Melipona beecheii), which typically range from 22.4 to 66.4 meq/kg. This suggests the high acidity may be due to increased fermentation, potentially stemming from factors like differences in bee species, geographical origin, improper handling, or high moisture content, which requires further investigation.” |
| 21 | Under acidity section , the author mentioned that sample was purchased .However they didn’t mentioned in methodology part . Sample collection should clearly indicated under methodology part otherwise it confuse the reader | corrected by mentioning the three purchased honey samples in the M&M section in the revised manuscript |
| 22 | CORRECT 3.1 Acidity to Free acidity | Corrected to “Free acid” in the revised manuscript |
| 23 | The overall mean moisture content of the 24 stingless bee honey samples was 23.45-36.75% is not good sentence .rewrite again | The overall moisture content of the honey samples was reported to be between 23.45% and 36.75%. |
| 24 | Table 1 should be come after paragraph of free acidity | Table 1 relocated as suggested |
| 25 | The paragraph arrangement is not good .it must be justified | Justified according to the provided suggestion |
| 26 | This finding is consistent with previous studies. Gela et al. (2021) reported an overall pH range of 3.4-3.9 (mean 3.7±0.15) for stingless bee honey from four districts in Ethiopia. Remove full stop between studies and Gela | Dot removed as per the suggestion |
| 27 | The standard pH value for Apis mellifera honey is 3.42–6.10 (Tesfaye et al., 2016). Are you sure with sentence or statement. In my opinion, 6.10 is not standard value of pH . Be sure also the citation .revise again. | Yes, I am sure. the standard pH range for Apis mellifera (European honeybee) honey is typically between 3.4 to 6.1, with an average around 3.9.  Addition to Tesfaye et al 2016, a review by Bogdanov et al. (2008) reported the pH of Apis mellifera honey to generally range from 3.4 to 6.1. |
| 28 | what your sample size . under materials and method s the author mentioned that 24 sample was collected .But on the table 1 the author stated that the sample size(N)=27. Which one is correct? It need revision | Corrected in the M& M section of the revised manuscript |
| 29 | Put acronomy for all abbreviation found in the table | all acronyms for all abbreviation in the table were presented under Table 1 in the revised document |
| 30 | The way of data presentation is not good for this article. I recommend the authors to use standard table or journal guidelines. Additionally, I recommend to put the result by mean ± std with mean separation letters . I observe level of significance in body of manuscript .but there is no in a table .How?. Please rewrite again | Rewritten. the result was put by mean ± std. In the table the p-value indicated showing level of significance, the standard table or journal guidelines used and all the comments were corrected accordingly |
| 31 | 3.3 Mineral (ash) contents correct to Ash content only remove mineral | corrected |
| 32 | Under 3.3. I recommend the authors to start the paragraph by presenting the result not discussion with literature .Remove first sentence | first sentence was removed and corrected |
| 33 | M.beccarii should be in italic form in all manuscript | Corrected |
| 34 | Be carefull when reporting the facts .for example “The higher mineral content of the stingless bee honey from the Dandi and Meta Robi districts indicates that it may be a better choice for both medicinal and nutritional purposes, provided that the honey is properly standardize” . I don’t think so that higher mineral content indicate medicinal value of honey .Rather there is parameters that indicate medicinal and nutritional value of honey. Please revise and cite the above sentence. | Revise well in the revised manuscript |
| 35 | under 3.4 Sugar profile need more discussion and citation . Not enough now. Compare current result with standards of stingless bee honey in other country. | Extensively edited and citations included |
| 36 | under 3.5 plesae start the paragragh by mentioning the obtained result .discussion come after presenting the finding. | Rearranged according to the comment |
| 37 | In this study, 0.06 and 4.2.0 values were recorded as minimum and maximum. 4.2.0 correct this one | Corrected to 4.20 |
|  | Please use style uniform typing for et al . in some areas it is in italic form and in some areas it is non- italic- | The issue was automatically solved when reference style changed |
| 38 | how high free acidity related to potentiality of medicinal value | Extensively addressed in the revised manuscript by supporting with various literature outputs |
| 39 | In conclusion I need a finding related both source of sample collected results | Revised well in the manuscript |

1. Title should be modified? It say properties of stingless bee, what properties? There are a lot of properties. Please specify that properties

Modified as “***Physicochemical*** ***properties of stingless bees (meliponula beccarii) honey in Dandi and Meta Robi districts of West Shewa zone, Ethiopia”***
2. It is not necessary to write Title: Properties of stingless bees (Meliponula beccarii) honey in Dandi and Meta Robi districts of West Shewa zone, Ethiopia. Starting by name of the article title is possible: corrected
3. I strongly recommend the authors if follow the journal formatting guidelines starting from Title formatting to Reference writing since the manuscript didn’t prepared based on the journal guidelines: Followed in the revised manuscript
4 .correct affiliation and corresponding author according to journal formatting guidelines: corrected in the revised manuscript
Abstact
5. Be careful for terminology of the parameter. For example free acidity not acidity , electrical
Conductivity not electric conductivity: Corrected in the revised manuscript
6.put correct standard unit for each parameter in abstract .example free acidity (meq/ Kg) not mill./kg, mS/cm not ms/cm (Corrected in the revised manuscript)
7. put SI for moisture content: The SI (International System of Units) unit for moisture content is percent (%) by weight (w/w).
8. I recommend ash content not mineral correction accepted and corrected accordingly
9. “The distinctive low HMF, high acidity and low pH values of the stingless bee honey of this study may represent freshness and good quality of the honey and indicative medicinal potential” . I didn ’t think high acidity indicate fresheness of honey, in this conntarst high acidity indicate fermentation rate of honey . the free acidity of obtained in this extremely high as compared to previous study conducted in west shoa and other parts of the country ,this need strong justification .may be during sample collecton orr harvesting mixing sample with soil,pollen ,and other dead and imputirtes cause honey to be fermented and high acidity

Corrected in the revised document

10.” This study evaluated physicho-chemical properties of honey produced by ground nesting stingless: bee (Meliponula beccarii) in two districts of West Shewa zone,” this is redundant since the author mentioned in firstyt line of abstract .please remove and re –edit again
11.what cause your study unique since different study was reported in west shoa.

The unique findings from the data are that the stingless bee (*Meliponula beccarii*) honey from the two districts exhibits high acidity, low pH, low HMF, and a distinctive sugar profile, differentiating it from other places studied stingless bee honey indicating potential medicinal properties”

Introduction
11.please folloew journal juideline starting from heading : Suggestions accepted and abide

12. reference citation is in number form for this journal.: Suggestions accepted and corretions done in the revised manuscript
13 .The name of Meliponula beccarii must be in italized form: Suggestions accepted and corretions done in the revised manuscript
14. if damma daamu local name it must be in italic form: Suggestions accepted and corretions done in the revised manuscript
15. Previous work and limitation /gap on characterization of stingless bee honey in that area as well as in Ethiopia must be clearly indicated: Suggestions accepted and corrections done in the revised manuscript
16. Paragraph arrangement of the introduction is not good: Suggestions accepted and corrections done in the revised manuscript
Materials and method
17. I recommend if there is sub heading under materials and methods section, unless this arrangement confuse the reader: Suggestions were accepted and corrections on the su-heading were done in the M&M parts of the revised manuscript
18. Figure 2, especially on the bottle containing sample is not visible, remove or replace with good picture: The invisible bottles bottles removed and replaced with the visible one

Result and discussion
19. on the result obtained on the free acidity, I strongly doubt on the result obtained (338.79 ± 72.46) . what is reason behind extremely high free acidity , is soil and other impurities is mixed in to honey during honey collection or harvesting .the author must discuss and compare with other data in Ethiopia. The high acidity and low pH observed in the *Meliponula beccarii* honey studied appear to be unusually high. This suggests the high acidity may be due to increased fermentation due to differences in bee species and geographical origin (Souza et al., 2006), improper handling and processing (Al-Tikrity et al., 1989), and high moisture content (Al-Tikrity et al., 1989).

20. I recommend the author also to compare the result with standard stingless bee honey in other countries (like brazil,venzuala and etc.) ,please refer there is standard of stingless bee honey in other country.): this is already discussed in the revised manuscript under the **free acidy section** as “The *Meliponula beccarii* honey studied appears to have unusually high free acidity and low pH compared to stingless bee honeys from Brazil (Melipona scutellaris), Venezuela (Plebeia saiqui, Tetragonisca angustula), and Mexico (Melipona beecheii), which typically range from 22.4 to 66.4 meq/kg. This suggests the high acidity may be due to increased fermentation, potentially stemming from factors like differences in bee species, geographical origin, improper handling, or high moisture content, which requires further investigation.”
21. Under acidity section , the author mentioned that sample was purchased .However they didn’t mentioned in methodology part . Sample collection should clearly indicated under methodology part otherwise it confuse the reader: corrected by mentioning the purchased honey samples in the M&M section in the revised manuscript
22. CORRECT 3.1 Acidity to Free acidity: Corrected to “Free acid” in the revised manuscript
23. The overall mean moisture content of the 24 stingless bee honey samples was 23.45-36.75% is not good sentence .rewrite again: rewritten as “The overall moisture content of the honey samples was reported to be between 23.45% and 36.75%.”
24.Table 1 should be come after paragraph of free acidity: Table 1 relocated as suggested
25.The paragraph arrangement is not good .it must be justified .: Justified according to the provided suggestion
26. This finding is consistent with previous studies. Gela et al. (2021) reported an overall pH range of 3.4-3.9 (mean 3.7±0.15) for stingless bee honey from four districts in Ethiopia. Remove full stop between studies and Gela. Dot removed
27 . The standard pH value for Apis mellifera honey is 3.42–6.10 (Tesfaye et al., 2016). Are you sure with sentence or statement. In my opinion, 6.10 is not standard value of pH . Be sure also the citation .revise again. Yes, I am sure. the standard pH range for Apis mellifera (European honeybee) honey is typically between 3.4 to 6.1 (it is not 6.1 as reviwer mentioned), with an average around 3.9. Addition to Tesfaye et al 2016, a review by Bogdanov et al. (2008) reported the pH of Apis mellifera honey to generally range from 3.4 to 6.1.
28 . what your sample size . under materials and method s the author mentioned that 24 sample was collected .But on the table 1 the author stated that the sample size(N)=27. Which one is correct? It need revision: Corrected in the M& M section of the revised manuscript
29 . Put acronomy for all abbreviation found in the table. all acronyms for all abbreviation in the table were presented under Table 1 in the revised document
30. The way of data presentation is not good for this article. I recommend the authors to use standard table or journal guidelines. Additionally, I recommend to put the result by mean ± std with mean separation letters . I observe level of significance in body of manuscript .but there is no in a table .How?. Please rewrite again. Rewritten. the result was put by mean ± std. In the table the p-value indicated showing level of significance
30. 3.3 Mineral (ash) contents correct to Ash content only remove mineral: Corrected
31. Under 3.3. I recommend the authors to start the paragraph by presenting the result not discussion with literature .Remove first sentence: first sentence removed and corrected
32. M.beccarii should be in italic form in all manuscript: Corrected
33.Be carefull when reporting the facts .for example “The higher mineral content of the stingless bee honey from the Dandi and Meta Robi districts indicates that it may be a better choice for both medicinal and nutritional purposes, provided that the honey is properly standardize” . I don’t think so that higher mineral content indicate medicinal value of honey .Rather there is parameters that indicate medicinal and nutritional value of honey. Please revise and cite the above sentence. Revise well in the revised manuscript
34. under 3.4 Sugar profile need more discussion and citation . Not enough now. Compare current result with standards of stingless bee honey in other country. Extensively edited and citations included
35. under 3.5 plesae start the paragragh by mentioning the obtained result .discussion come after presenting the finding. Rearranged according to the comment
36. In this study, 0.06 and 4.2.0 values were recorded as minimum and maximum. 4.2.0 correct this one. Corrected to 4.20
37 . Please use style uniform typing for et al . in some areas it is in italic form and in some areas it is non- italic- The issue was automatically solved when reference style changed

Conclusion
38 . how high free acidity related to potentiality of medicinal value . Extensively addressed in the revised manuscript by supporting with various literature outputs
39. In conclusion I need a finding related both source of sample collected results

Reviewer #3: Reviewer report of the Manuscript ID PONE-D-24-28380

Table-3 response to reviwer-3 comments

| **No** | **Comments** | **Provided Responses** |
| --- | --- | --- |
|  | GENERAL |  |
| **1** | all sections need to be organized, especially the Abstract, Material and methods, and Conclusion sections | All reorganized (reflected in the revised manuscript) |
| **2** | the scientific names are incorrectly put in non-italic form | Corrected |
| **3** | Figures needed to be further elaborated. | Done (fig.1 removed) and blurred honey bottle changed) |
|  | ABSTRACT |  |
| **4** | Please rectify “The differences between the two districts honey samples are significant (p> 0.05) in glucose value”. The p-value is not significant | Apology for the mistake. The significant point was not for glucose, but, for fructose. It is corrected this time |
| **5** | Please put all names of “A.mellifera” due to being first mentioned | Corrected to Apis mellifera |
|  | INTRODUCTION |  |
| **6** | Please rectify “(Apis)” by putting all scientific microbial names in italics. Also, check the remaining text for this error | All checked and corrected |
|  | MATERIALS AND METHODS |  |
| **7** | Please correct Figure 1, it is a print screen still with red underlines | Figure 1 removed |
| **8** | Please clarify: “From each nest, fresh honey samples ranging from 0.25 to 0.5 liters were collected” but in the abstract, the authors stated “A total of 24 honey samples with each 0.58–0.69-liter were collected”. | Corrected to the value in the revised abstract |
| **9** | The Material and methods section shows no clear division of the techniques applied. Please reorganize this section with proper subsections and cite all procedures applied. | Clear divisions have been added in the revised materials and method section |
|  | RESULTS AND DISCUSSION |  |
| **10** | Table 1- Please clarify: “Mean physicochemical properties of stingless bee honey samples (N = 27)” but the authors previously stated 24 honey samples or 24 stingless bee nests in the Abstract and Material and methods sections. | Nicely corrected |
| **11** | Purchased honey samples must be added in the Abstract section, I already saw that these purchased honey samples were described in the Material and methods section, but improvements are needed in both sections. | Improved and the below statement was added to the abstract part “The honey samples purchased from the market showed similar physicochemical properties to the honey from the feral nests, with a mean of 314.33±88.72 meq./kg free acidity, 27.73±2.52% moisture content, 1.43±0.41 mS/cm electrical conductivity, 3.26±0.13 pH, 0.95±1.23 mg/kg HMF, 0.59±0.19% mineral (ash), 10.11±4.11% glucose, 0.25±0.02 g/100g sucrose, 11.23±4.52 g/100g maltose, and 16.33±2.41 g/100g fructose”. |
|  | CONCLUSION |  |
| **12** | Please add the shortcomings of the present study | The study's shortcomings include its limited scope, lack of seasonal variations, absence of bioactive compound profiling, and lack of comparative analysis |
|  | REFERENCES | Automatically solved when the reference style changes |

Congratulations on the present study! It is an interesting work with relevant data, but it is a preliminary analysis and I do not know if it is more suitable for a short report or research note than a full-length study. However, it is an Editor's decision and several improvements must be realized by the authors in the revised version of the manuscript.
General comments
The manuscript is well-written, and, in my opinion, no major English Editing is needed. However, all sections need to be organized, especially the Abstract, Material and methods, and Conclusion sections. All reorganized (reflected in the revised manuscript)
Also, the scientific names are incorrectly put in non-italic form. Corrected
Figures needed to be further elaborated. Please see my minor comments below. Done (fig.1 removed) and blurred honey bottle changed)
Minor comments
Abstract
Please rectify “The differences between the two districts honey samples are significant (p> 0.05) in glucose value”. The p-value is not significant. Apology for the mistake. The significant point was not for glucose, but, for fructose. It is corrected this time
Please put all names of “A.mellifera” due to being first mentioned. All checked and corrected
Introduction
Please rectify “(Apis)” by putting all scientific microbial names in italics. Also, check the remaining text for this error. All checked and corrected according to the comment
Material and methods
Please correct Figure 1, it is a print screen still with red underlines. Figure 1 removed and correction accepted
Please clarify: “From each nest, fresh honey samples ranging from 0.25 to 0.5 liters were collected” but in the abstract, the authors stated “A total of 24 honey samples with each 0.58–0.69-liter were collected”. Corrected to the value in the revised abstract
The Material and methods section shows no clear division of the techniques applied. Please reorganize this section with proper subsections and cite all procedures applied. Clear divisions have been added in the revised materials and method section
Results and discussion
Table 1- Please clarify: “Mean physicochemical properties of stingless bee honey samples (N = 27)” but the authors previously stated 24 honey samples or 24 stingless bee nests in the Abstract and Material and methods sections. Nicely corrected in the revised manuscript
Purchased honey samples must be added in the Abstract section, I already saw that these purchased honey samples were described in the Material and methods section, but improvements are needed in both sections. Improved and the below statement was added to the abstract part “The honey samples purchased from the market showed similar physicochemical properties to the honey from the feral nests, with a mean of 314.33±88.72 meq./kg free acidity, 27.73±2.52% moisture content, 1.43±0.41 mS/cm electrical conductivity, 3.26±0.13 pH, 0.95±1.23 mg/kg HMF, 0.59±0.19% mineral (ash), 10.11±4.11% glucose, 0.25±0.02 g/100g sucrose, 11.23±4.52 g/100g maltose, and 16.33±2.41 g/100g fructose”.
Conclusion
Please add the shortcomings of the present study. comment accepted and corrected as “The study's shortcomings include its limited scope, lack of seasonal variations, absence of bioactive compound profiling, and lack of comparative analysis”
References: Automatically solved when the reference style changes
Please revise all citations. For example:
11. hilmi, M., & Martin hilmi. (2005). The Marketing of bee products.
12. Kahraman. (2010). Physico-chemical properties in honey from different regions of Turkey.
.." FOOD CHEMISTRY, 123(1), 41–44
24. Quality and Standards Authority of Ethiopia (QSAE). (2005). Honey Specification:
Ethiopian Standard, ES 1202. Addis Ababa, Ethiopia.
28. Stramm, K. M. (2011). Composition and Quality of Honey Bee Jandaira (Melipona
Subnitida), Storage Effects and Comparison with Apis mellifera Honey.
29. Teferi Damto, D. K. and M. (2022). Physico-chemical and microbiological characteristics of honey produced by stingless bees.
Among others.

Again, congratulations on the present work! Best regards

6. PLOS authors have the option to publish the peer review history of their article ([what does this mean?](https://journals.plos.org/plosone/s/editorial-and-peer-review-process#loc-peer-review-history)). If published, this will include your full peer review and any attached files.


If you choose “no”, your identity will remain anonymous but your review may still be made public.


**Do you want your identity to be public for this peer review?** For information about this choice, including consent withdrawal, please see our [Privacy Policy](https://www.plos.org/privacy-policy).

Reviewer #1: **Yes:** Fahrul Huyop

Reviewer #2: No

Reviewer #3: No

[NOTE: If reviewer comments were submitted as an attachment file, they will be attached to this email and accessible via the submission site. Please log into your account, locate the manuscript record, and check for the action link "View Attachments". If this link does not appear, there are no attachment files.]

While revising your submission, please upload your figure files to the Preflight Analysis and Conversion Engine (PACE) digital diagnostic tool, <https://pacev2.apexcovantage.com/>. PACE helps ensure that figures meet PLOS requirements. To use PACE, you must first register as a user. Registration is free. Then, login and navigate to the UPLOAD tab, where you will find detailed instructions on how to use the tool. If you encounter any issues or have any questions when using PACE, please email PLOS at [figures@plos.org](mailto:figures@plos.org). Please note that Supporting Information files do not need this step.

*In compliance with data protection regulations, you may request that we remove your personal registration details at any time.* [*(Remove my information/details)*](https://www.editorialmanager.com/pone/login.asp?a=r)*. Please contact the publication office if you have any questions.*

**2 attachments** • Scanned by Gmail
