# Supplementary material for: Physicochemical properties of stingless bees (Meliponula beccarii) honey in Dandi and Meta Robi districts of West Shewa zone, Ethiopia
Source: PLoS One. 2024 Dec 12;19(12):e0311725. doi: 10.1371/journal.pone.0311725 (PMC11637301; doi:10.1371/journal.pone.0311725)
Supplement: Attachment — Submitted filename: Response to Reviewers.docx [file pone.0311725.s005.docx]

Response to Reviewers

PONE-D-24-28380R1

Physicochemical properties of stingless bees (*meliponula beccarii*) honey in Dandi and Meta Robi districts of West Shewa zone, Ethiopia

PLOS ONE

6. Review Comments to the Author

Please use the space provided to explain your answers to the questions above. You may also include additional comments for the author, including concerns about dual publication, research ethics, or publication ethics. (Please upload your review as an attachment if it exceeds 20,000 characters)

**Reviewer #1:** MINOR CORRECTIONS: Please highlight these changes in RED/BLUE in your corrected manuscript

a) Introduction Section:
Please include a brief mention of Meliponula beccarii as an important stingless bee species in Ethiopia, with appropriate references. Highlight that this species is commonly found in the Dandi and Meta Robi districts of the West Shewa zone, Ethiopia.

*Meliponula beccarii*, a species of stingless bee, is commonly found in Ethiopia and is well known for its honey production as well as its contributions to local biodiversity and pollination. It is particularly prevalent in the Dandi and Meta Robi districts of the West Shewa zone, where it plays a vital role in the ecosystem.

b) Results and Discussion Section:

(i) Figure 2: The current image of the water bottle is not ideal. We need to clearly see the color of the honey (green/yellow/dark). Please describe the color more clearly and replace the image with a better one.

The image of the water bottle removed

(ii) Please be cautious with the phrase “to indicate medicinal value of honey.” It would be more appropriate to say: “indicate the medicinal and nutritional value of honey.” Kindly check and revise this. Corrected accordingly (on page 10 of the revised manuscript)

(iii) The discussion on the sugar profile needs further elaboration and additional citations (Please compare with other findings the data from Stingless Bee as well). The comparison with the Codex Alimentarius standard is very appropriate maintain as it is, BUT should also include comparisons with stingless bee honey from other regions like Southeast Asia (Malaysia, Indonesia, Thailand) and South America- Brazil/Venezuela etc..
I recommend comparing your results with the standards for stingless bee honey from other countries. Please refer to existing standards for stingless bee honey in these regions (IF ANY).

Others are OK!

Thank you for your constructive comments on our manuscript. We greatly appreciate your input, as it has helped us enhance the quality of our work.

In addition to addressing your suggestions, we have included comparisons for each physicochemical parameter with stingless bee honey from other countries in Southeast Asia and South America in the table and body of the revised manuscript. We believe this additional context strengthens our findings.

**Reviewer #2**:

Abstract
1. Check maltose content result because it is extremely high .compare with other findings

Previous studies has reported maltose levels in stingless bee honey generally range from lower values, typically around 1% to 5% (22,23). However, the high maltose content in our stingless bee honey may be due to several factors like specific floral sources can significantly influence sugar composition, with certain plants containing higher maltose concentrations (22), fermentation processes within the nest may also increase maltose levels if honey is stored for extended periods (23), the foraging behavior of stingless bees that could lead to the preferential collection of nectar from maltose-rich plants and environmental factors, such as soil composition and climate, that may further impact floral diversity and nectar quality, resulting in elevated maltose levels (53), processing and storage methods might alter sugar profiles, with prolonged storage or heat affecting composition (24), variability among stingless bee species (23).

2.Again I inform the authors that high acidity (Free acidity) is not related with quality and medicinal properties .High acidity of stingless bee related to fermentation rate of this honey because stingless bee honey contain high moisture and alcohol is easily formed in it that is way free acidity is high.
High quality and medicinal properties of stingless bee is related to strong pH and high antioxidant properties.

Higher free acidity in honey can enhance flavor complexity, inhibit microbial growth to extend shelf life (Tan et al., 2021), and provide potential health benefits, including antibacterial properties from organic acids (Tey et al., 2022).

3. The result of turanose was not presented in abstract:

Included in the abstract of the revised manuscript
Materials and methods: In the M&M, the analysis of turanose was similar as fructose, glucose, and sucrose and already stated as “A standard curve was used to determine sugar content (fructose, glucose, sucrose, turanose, and maltose) and the values were calculated as gram/100gram of honey sample”

**Results and Discussion**

4. Please free acidity more acceptable than acidity on sub heading (corrected accordingly)
5. The authors stated that free acidity was extremely high in this study and also mentioned this is because of different species and agro ecologies. But since different study was conducted in West Shoa as the author stated in introduction, why you don’t compare you’re finding with those study conducted in same area and same country. The study lack literature discussion in Ethiopia.
Or high acidity because of your sample impurity? Please discuss carefully on the finding. I didn’t see improvement.

Reports from Ethiopia has been included and discussed well with plausible justifications added for why free acidity was high in our honey sample in the manuscript
6. The line and paragraph spacing and font color is not uniform in all paragraph (corrected accordingly)
7. Correct sub heading of moisture content (corrected accordingly)
8. Font color and size of sub heading should be improved ((corrected accordingly)
9. In abstract the authors stated elevated pH value but in results and discussion parts, the finding of the study is similar with other finding conducted in Ethiopia. Revise abstract or discussion parts. The abstract part revised.

10. The arrangement of the paragraph is week
11. For me discussion of sugar profile is not enough? Needs to be revised again by comparing the finding of the study with other similar findings. Well discussed this time in the revised manuscript

12. Under HMF paragraph please correct the paragraph (corrected)
13. Why fresh collected and marketed ones was not compared in finding. It is better if both types of samples was compared parallel. Both types of samples were compared parallel at every parameter

**Conclusion**
14.“This study evaluated This study evaluated “. Please remove redundant sentence (Removed accordingly)

15.” This study evaluated the physicochemical properties of honey produced by the ground-nesting stingless bee (Meliponula beccarii) in Ethiopia” , by collecting sample from two woreda of Oromia region we can say this study represent Ethiopia. Please revise this one again. (Revised)

16 .revise the conclusion based on your fining especially on fresh and marketed honey sample variability (revised)

17. lack of consistent formatting of reference: We utilized the Mendeley desktop reference manager to format our references according to the Vancouver style outlined in the PLOS author guidelines. We believe this will ensure consistent formatting throughout the reference section. Additionally, we have made adjustments to enhance the presentation and engage the reviewer more effectively.
